# Targeted phage hunting to specific *Klebsiella pneumoniae* clinical isolates is an efficient antibiotic resistance and infection control strategy

**DOI:** 10.1128/spectrum.00254-24

**Published:** 2024-08-28

**Authors:** Celia Ferriol-González, Robby Concha-Eloko, Mireia Bernabéu-Gimeno, Felipe Fernández-Cuenca, Javier E. Cañada-García, Silvia García-Cobos, Rafael Sanjuán, Pilar Domingo-Calap

**Affiliations:** 1 Instituto de Biología Integrativa de Sistemas, Universitat de València-CSIC, Paterna, Spain; 2 Unidad Clínica de Enfermedades Infecciosas y Microbiología, Hospital Universitario Virgen Macarena, Sevilla, Spain; 3 Instituto de Biomedicina de Sevilla, Hospital Universitario Virgen Macarena-CSIC-Universidad de Sevilla, Sevilla, Spain; 4 CIBER de Enfermedades Infecciosas (CIBERINFEC), Instituto de Salud Carlos III, Madrid, Spain; 5 Laboratorio de Referencia e Investigación en Resistencia a Antibióticos e Infecciones Relacionadas con la Asistencia Sanitaria, Centro Nacional de Microbiología, Instituto de Salud Carlos III, Madrid, Spain; University of Pittsburgh School of Medicine, Pittsburgh, Pennsylvania, USA

**Keywords:** *Klebsiella pneumoniae*, phage hunting, phage therapy, phage cocktail, host range, bacterial capsule, receptor-binding protein, depolymerase

## Abstract

*Klebsiella pneumoniae* is one of the most threatening multi-drug-resistant pathogens today, with phage therapy being a promising alternative for personalized treatments. However, the intrinsic capsule diversity in *Klebsiella* spp. poses a substantial barrier to the phage host range, complicating the development of broad-spectrum phage-based treatments. Here, we have isolated and genomically characterized phages capable of infecting each of the acquired 77 reference serotypes of *Klebsiella* spp.*,* including capsular types widespread among high-risk *K. pneumoniae* clones causing nosocomial infections. We demonstrated the possibility of isolating phages for all capsular types in the collection, revealing high capsular specificity among taxonomically related phages, in contrast to a few phages that exhibited broad-spectrum infection capabilities. To decipher the determinants of the specificity of these phages, we focused on their receptor-binding proteins, with particular attention to depolymerases. We also explored the possibility of designing a broad-spectrum phage cocktail based on phages isolated in reference capsular-type strains and determining the ability to lyse relevant clinical isolates. A combination of 12 phages capable of infecting 55% of the reference *Klebsiella* spp. serotypes was tested on a panel of carbapenem-resistant *K. pneumoniae* clinical isolates. Thirty-one percent of isolates were susceptible to the phage cocktail. However, our results suggest that in a highly variable encapsulated bacterial host, phage hunting must be directed to the specific *Klebsiella* isolates. This work is a step forward in the understanding of the complexity of phage–host interactions and highlights the importance of implementing precise and phage-specific strategies to treat *K. pneumoniae* infections worldwide.

**IMPORTANCE:**

The emergence of resistant bacteria is a serious global health problem. In the absence of effective treatments, phages are a personalized and effective therapeutic alternative. However, little is still known about phage–host interactions, which are key to implementing effective strategies. Here, we focus on the study of *Klebsiella pneumoniae,* a highly pathogenic encapsulated bacterium. The complexity and variability of the capsule, where in most cases phage receptors are found, make it difficult for phage-based treatments. Here, we isolated a large collection of *Klebsiella* phages against all the reference strains and in a cohort of clinical isolates. Our results suggest that clinical isolates represent a challenge, especially high-risk clones. Thus, we propose targeted phage hunting as an effective strategy to implement phage-derived therapies. Our results are a step forward for new phage-based strategies to control *K. pneumoniae* infections, highlighting the importance of understanding phage–host interactions to design personalized treatments against *Klebsiella* spp.

## INTRODUCTION

*Klebsiella pneumoniae,* an opportunistic Gram-negative encapsulated bacterium, belongs to the ESKAPE group that comprises some of the most critical multi-drug-resistant (MDR) bacterial pathogens (1, 2). Worldwide, more than 69,000 deaths per year are associated with MDR *K. pneumoniae*, thus being considered a major threat (3). Indeed, *K. pneumoniae* is considered the fastest-growing cause of bacterial infections in the European region currently, with a high prevalence in hospital-acquired infections (4, 5). Although it typically colonizes the human gastrointestinal tract asymptomatically as commensals, in immunocompromised and critically ill patients, *K. pneumoniae* can lead to severe diseases such as pneumonia, urinary tract infections, bloodstream infections, and wound or surgical site infections (6). The main virulence factor of *Klebsiella* spp. is its capsule, which protects bacteria from the immune system and also from adverse environmental conditions and possible predators (7, 8). *Klebsiella* is a highly variable bacterium, with 77 capsule serotypes (9–11), identified as the reference capsular types. However, high-throughput sequencing and bioinformatics tools have led to more than 180 capsular locus types (KL types) described (12, 13). Traditionally, seven-gene multi-locus sequence typing (ST) nomenclature has been widely used to describe *K. pneumoniae* high-risk clones. Indeed, these STs can be associated with specific KL types (12). In Spain, the most abundant circulating in 2019 were ST307, ST11, ST512, ST15, ST147, and ST392 (14). Furthermore, many of these STs are distributed worldwide producing nosocomial outbreaks (5) and have accumulated different carbapenemases, with the most prevalent being *bla*_OXA-48_, *bla*_KPC-3_, *bla*_VIM-1_, and *bla*_NDM-1_ (14, 15).

The lack of effective treatments for MDR bacteria forces the implementation of alternative strategies, among which bacteriophage (phage) therapy emerges as a promising option (16). Phages are viruses that infect bacteria. They have been used for treating bacterial infections since their early discovery (17–19). However, for therapy, only strictly lytic phages are useful, avoiding phages encoding lysogenic genes and preventing gene transfer. A key feature of phages is their specificity. Most phages are capable of infecting a small subset of strains within a bacterial species (20, 21), while other phages exhibit broader host ranges, being able to infect even different bacterial species (22). Phages with a narrow host range may be considered advantageous for personalized phage therapy, as it allows the phage to target pathogenic bacteria without affecting the microbiota, reducing the risk of side effects (23). However, broad-range phages also present some advantages, since they can lyse a wide range of bacterial strains, making them particularly useful when multiple bacteria are responsible for an infection, or as preventive tools (24, 25).

Bacteria, as evolving entities, can develop phage resistance mechanisms. Indeed, it is known that *Klebsiella* spp. can emerge phage resistance in a few hours (26). Interestingly, phages can overcome this resistance, since they co-evolve with bacteria as an arms race between the two components (27). In this sense, both narrow- and broad-range phages are susceptible to inducing bacterial phage resistance, and phage cocktails may overcome or delay this emergence. Developing phage cocktails able to control bacteria as preventive tools is a challenge, since there are many limitations in the phage–host interactions. However, understanding phage diversity could provide new insights into developing targeted strategies for phage hunting and implementing new research lines. It is known that phage diversity is enormous, but we are far away from isolating and being able to grow and culture the vast majority of phages.

Targeted hunting to specific hosts could be an interesting approach to creating a large collection of phages with many applications. In this sense, *K. pneumoniae* is a major challenge due to its capsular-type diversity, which has been proposed as the main restrictive determinant of the phage host range. Understanding the mechanisms underlying the host range is crucial for developing new treatments, as host recognition serves as the first barrier and a major determinant (28). Phage receptor-binding proteins (RBPs) mediate the initial phage–host recognition, joining the receptors on the surface of the host (29). Bacterial capsules can mask cell receptors, protecting them from phage infection (30), although phages can also exploit them for their attachment (31, 32). To overcome the barrier that the capsule poses for the infection, some phages encode specific enzymes called depolymerases (Dpos), capable of recognizing, binding, and digesting specifically oligosaccharide bonds. Interestingly, phage Dpos are versatile domains exhibiting diverse structural conformations. These domains have been associated with polysaccharide degradation activity and can reside in either structural proteins (like tail spikes) or exist as soluble proteins (33, 34). In addition to this first-step barrier, post-adsorptive defense mechanisms have a role in phage infectivity, such as restriction modification mechanisms (35), abortive infection systems (36), regularly interspaced short palindromic repeats (CRISPRs) (37, 38), and many other defenses that have been discovered recently (39, 40).

A deeper understanding of the host range diversity in *Klebsiella* phages is needed to implement effective phage-based control strategies. This would enable the conception of phage cocktails, tailored to the epidemiological profile of a specific region. These “à la carte” cocktails would comprise phages with complementary host ranges, resulting in a broader spectrum of activity for the whole preparation. Additionally, they may exhibit synergistic effects, further enhancing their efficiency. Here, we have isolated and characterized a myriad of phages, suggesting the feasibility of developing precise and personalized treatments based on environmental phages. The evaluation of the cross-infectivity of the phages allowed us to unravel the underlying factors determining the host range of *Klebsiella* phages, showing that they can be associated in viral clusters. Deep analyses of the Dpos and RBPs reported unraveled diversity and provided the basis for understanding phage–host interactions. With this information, we designed a broad-spectrum phage cocktail against high-risk clones of *K. pneumoniae* circulating in Spain. Our results showed that the high specificity of *Klebsiella* phages and intrinsic bacterial defense mechanisms impair phage infectivity. Thus, phage hunting directed to the clinical strain could be a promising solution to combat *K. pneumoniae* infections. Indeed, we suggest that phage cocktails might be limited to prevention strategies or ready-to-use treatments, specifically designed for requirements in each region based on the epidemiology.

## RESULTS

### Phage diversity and distribution in viral clusters

**Fig 1:**
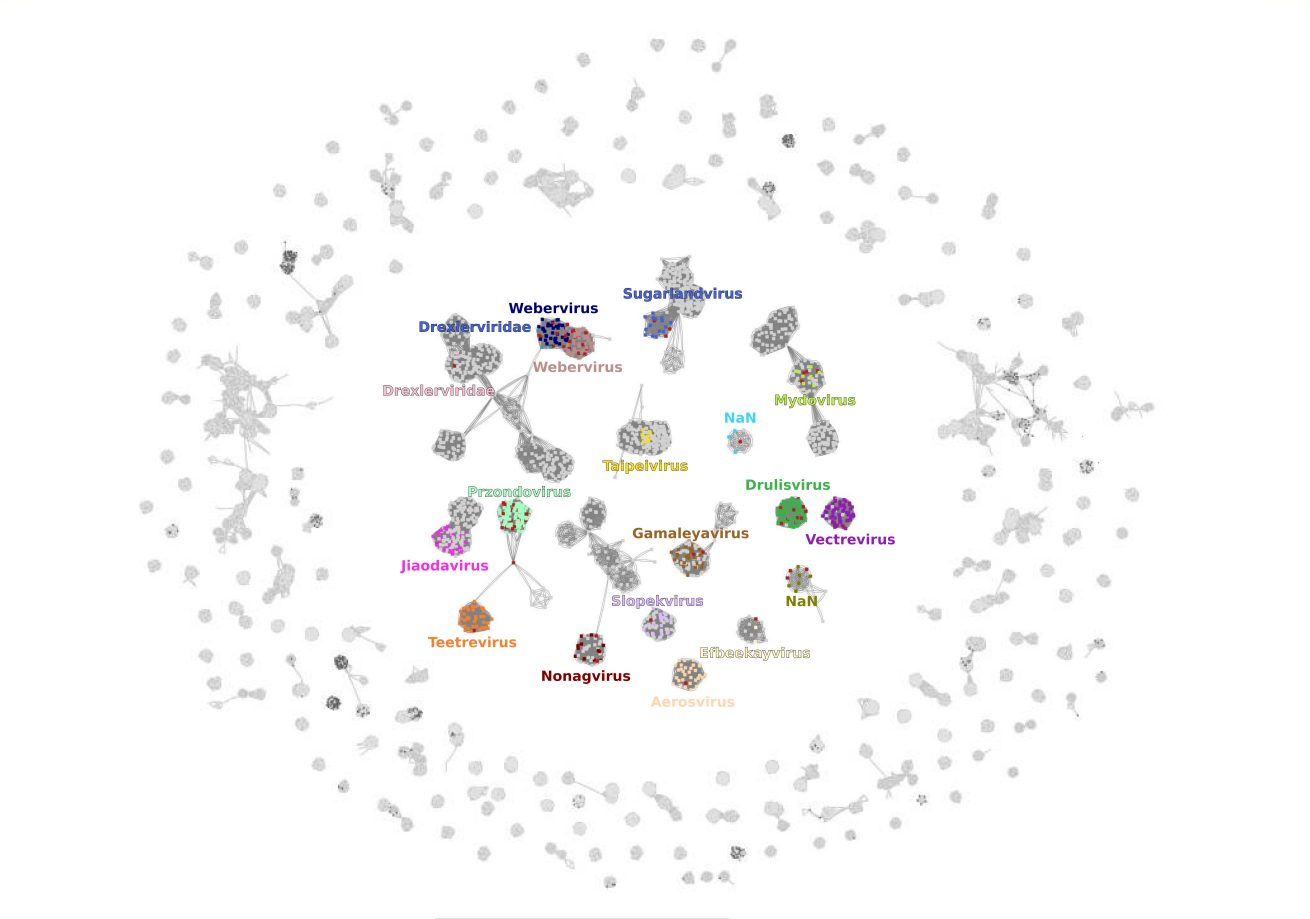
Viral cluster network including 76 isolated and characterized *Klebsiella* phages. The network analysis has been performed on protein profiles encoded by phages using the vConTACT2 software. Each of the *Klebsiella* phages isolated here is represented as red nodes. Previous phages belonging to the same VC are represented with specific color nodes. The gray nodes correspond to other phages that were used as references for our analysis. The graph was visualized using a force-directed layout through Cytoscape software (42). NaN, non-available name.

Extensive phage hunting from environmental samples in Valencia (Spain) was done using the 77 *Klebsiella* spp. reference strains as primary hosts. A total of 83 phages were isolated, and among them, 71 were successfully sequenced and characterized. The rest of them were not sequenced due to technical issues, probably due to their instability. Each of the strains was infected by at least one of the phages, suggesting the possibility of isolating *Klebsiella* spp. phages from the environment capable of infecting a large collection of *K. pneumoniae* isolates with different capsular types. Three phages of the genus *Sugarlandvirus* previously isolated in our group were included in the phage collection (41). The genome length of the phages varied significantly, ranging from 38,774 to 176,687 base pairs (bp) (Table S1). The isolated phages were distributed across 19 distinct viral clusters (VC) corresponding to several genera, with a confidence level higher than 0.9 (Fig. 1). These included *Webervirus* (18 phages), *Przondovirus* (15 phages), *Drulisvirus* (10 phages), *Vectreviru*s (5 phages), *Mydovirus* (4 phages), *Sugarlandvirus* (3 phages), *Gamaleyavirus* (2 phages), VC-818-0 (3 phages), VC-473-0 (1 phage), VC-590-0 (2 phages), VC-589-2 (1 phage), and *Efbeekayvirus*, *Teetrevirus*, *Slopekvirus*, *Aerosvirus*, *Taipeivirus*, *Jiaodavirus*, and *Nonagvirus* (each with a single phage). Four remaining phages were categorized as outliers.

### Phage genomic characterization and annotation

**Fig 2:**
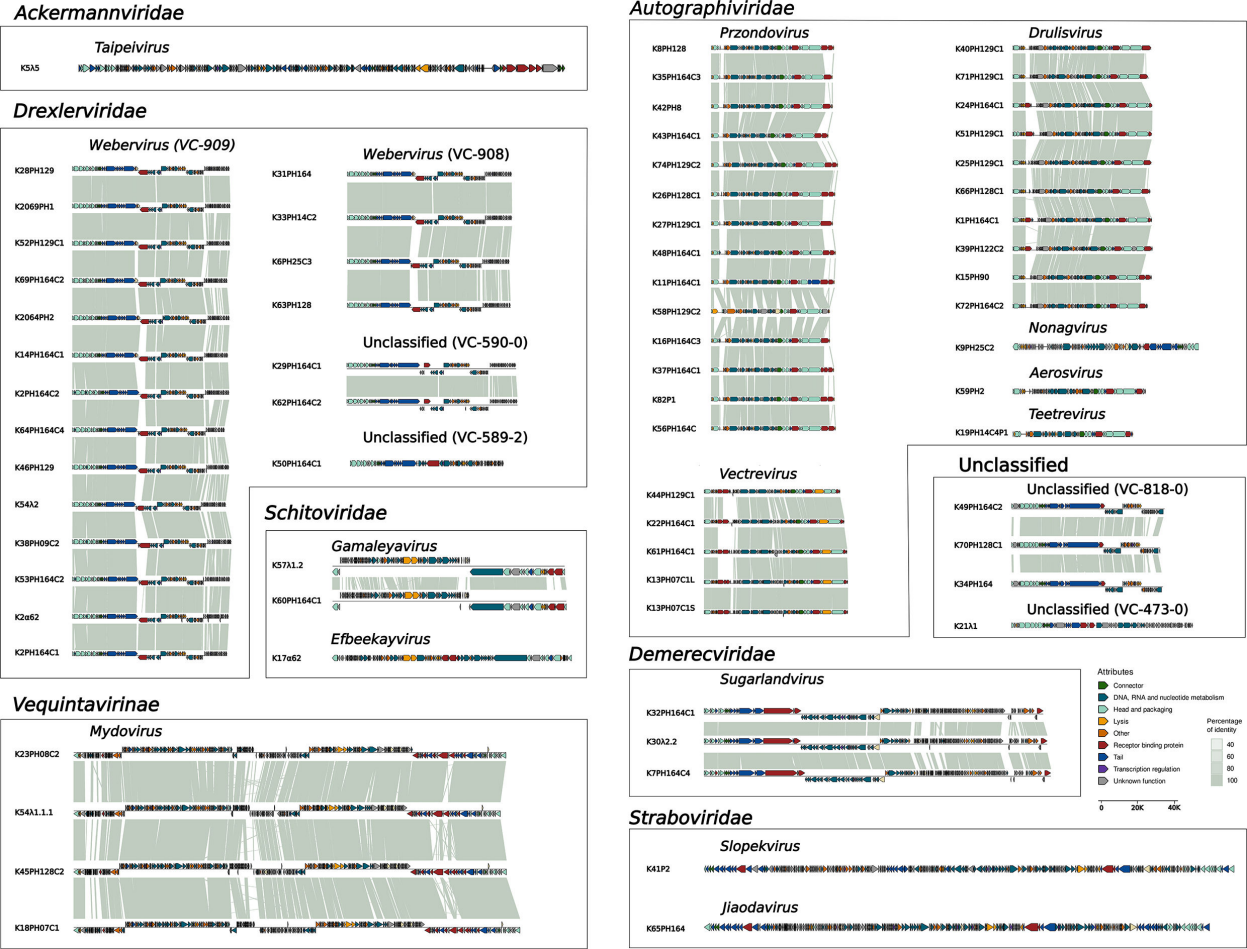
Intergenomic similarity between the isolated and characterized *Klebsiella* phages taxonomically classified. The viral clusters obtained after the vContact2 process were organized into family or subfamily and genus. The intergenomic similarities were computed using blastn (46). The arrows, oriented along the coding strand, represent the identified opening reading frames. Each color is related to a functional group, as defined in the PHROG database (43). Correspondence between the name of the phages and the alias is available in supplementary material.

The lifestyle and the virulence of the phages were predicted obtaining a probability value ranging from 0 to 1, which indicates its probability to exhibit a virulent phenotype. All isolated phages were predicted as lytic life cycles, with the probability ranging from 0.72 to 1 for over half of the phages, with the mean lytic life predicted probability for an isolated phage of 0.96 ± 0.05. Although genetic annotations were carried out using the remote homology search tool, HMMer, in conjunction with the PHROG database (43–45), a large fraction of genes remained with an unknown function (Table S2). Core genes across the phage genomes were categorized into defined functional groups. These included the large and small terminase subunits and the major head protein in the “head and packaging” group; HNH endonuclease and exonuclease in the “DNA, RNA, and nucleotide metabolism” category; the spanin, holin, and endolysin in the “lysis” category; and the prototypical tail proteins and tail length tape measure protein in “tail proteins,” along with tail fiber and Dpo proteins in the RBP category (Fig. 2). Alongside these conventional genes, our analysis also highlighted less conventional genes, like transcription regulation genes, and others associated with overcoming bacterial defense systems.

### Phage host recognition genes

**Fig 3:**
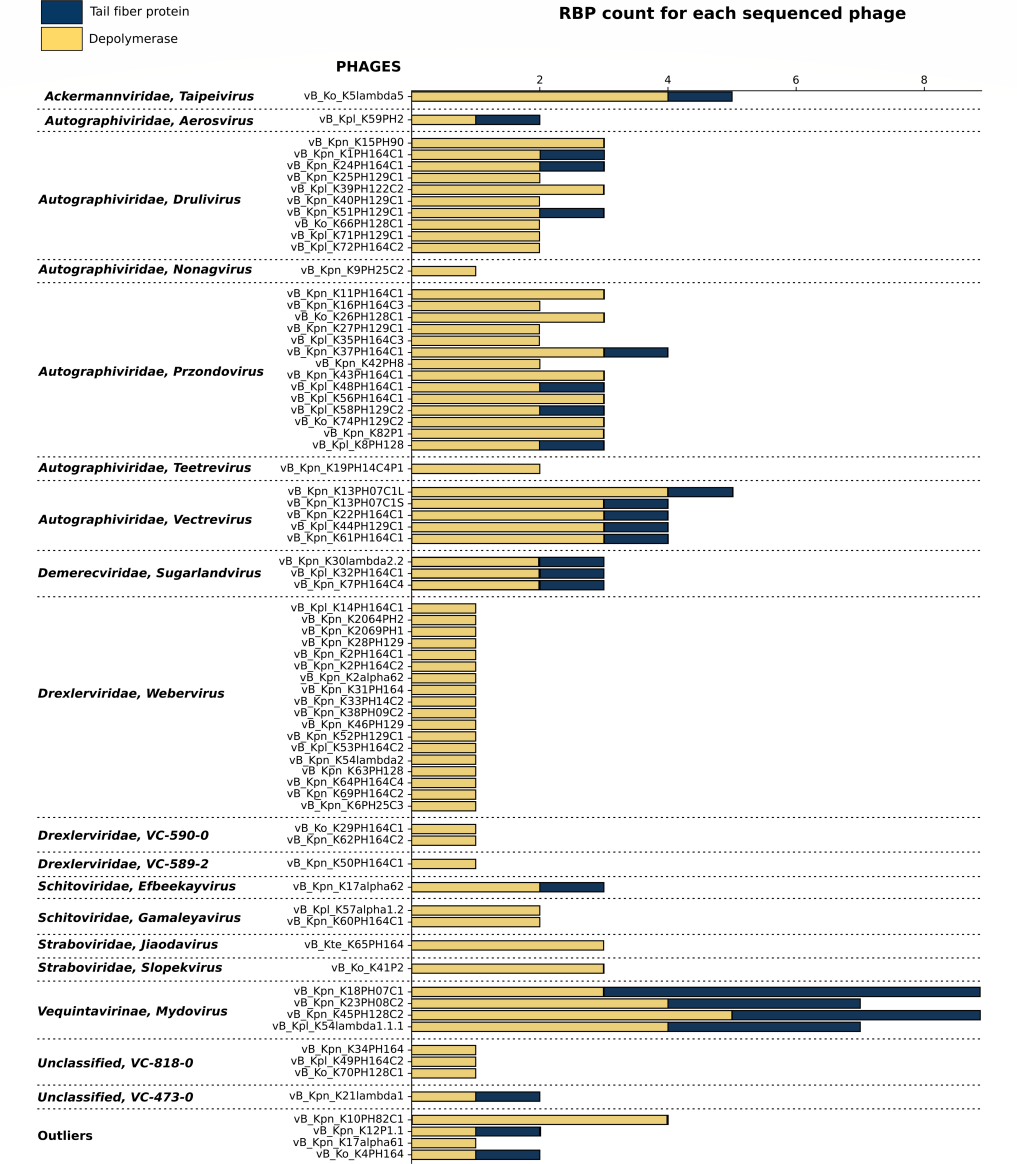
Count of the receptor-binding proteins (RBP) in the isolated and characterized *Klebsiella* phages arranged by viral cluster. The chart plot represents in the vertical axis each of the phages classified in their viral clusters. The horizontal axis indicates the total RBP count. The count of tail fibers is represented in blue and the count of depolymerases in gold.

In order to characterize phage host recognition genes, a total of 183 RBPs were identified, which included 147 Dpos (Fig. 3; Tables S3 and S4). The diversity and distribution of these RBPs varied significantly across different VCs. Surprisingly, at least one Dpo was detected in all the isolated phages. Among all phages, vB_Kpn_K45PH128C2 contained the highest count of Dpos, with five distinct Dpos identified in this single phage. The variation in the structural organization in the identified Dpo domains was remarkable (Fig. S1). The right-handed β-helix was the most prevalent fold, representing 74 of the identified Dpo domains, followed by the six-bladed β-propeller with 41 domains, 31 triple-helixes, and a single α/α toroid. The structural architecture of the Dpos was also diverse and organized into one or more domains. The simplest form consisted of a single domain Dpo, with either a right-handed β-helix or a six-bladed β-propeller. However, most Dpos were arranged in two domains, together with either the N-terminal domain or the jelly roll. Triple-helixes were most often found associated with a C-terminal intramolecular chaperone domain. In some instances, both the N-terminal domain and the jelly roll were found with a Dpo, forming a three-domain protein. Another configuration was observed in some Dpos with the six-bladed β-propeller associated with domains with unknown functions. Finally, more intricate structures like multiple-domain organization or baseplate assemblies with a six-bladed β-propeller were identified. A particularly intriguing example was found in phage vB_Ko_K41P2 (ORF 226), where the Dpo domain potentially forms an intramolecular trimer as hints of the presence of interspersed triple-helix domains. Furthermore, our analyses suggested a structural continuity between some tail fiber proteins and tail spike proteins. In fact, instances of tail fiber proteins that comprised the phage T7 N-terminal domain, an α-helix, and the jelly roll motif were observed. Both domains demonstrated structural similarity when aligned with Dpos (Fig. S2).

### Determination of phage tropism and infectivity

**Fig 4:**
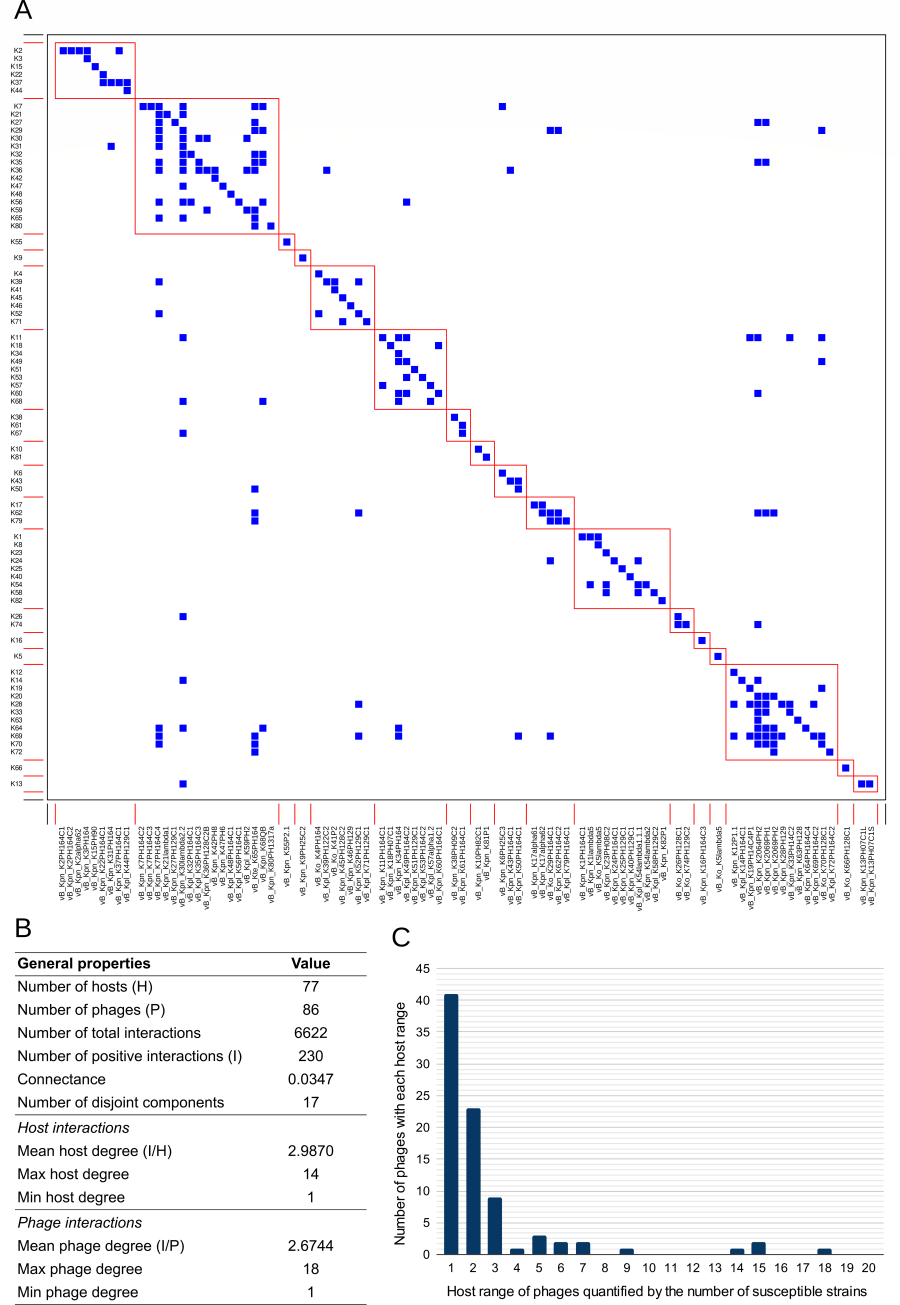
Host range of the 86 isolated *Klebsiella* phages against 77 *Klebsiella* reference serotypes. (**A**) Modular distribution of the cross-infection matrix representing the host range of the 86 isolated *Klebsiella* phages in each column over the 77 *Klebsiella* spp. reference strains in each row. Positive interactions are represented in blue, and negative interactions are represented in white. The 17 modules detected are squared in red [modularity value *Q* = 0.604 (*P*-value = 2.2 × 10^−16^)]. (**B**) General properties of cross-infection matrix, including host interactions and phage interactions. (**C**) Graphic representation of the number of phages among the 86 isolated with different host ranges. The horizontal axis represents the phage host range (number of susceptible reference strains), while the vertical axis represents the amount of *Klebsiella* phages for each group.

The host range of the 86 isolated phages was determined experimentally by a cross-infection matrix including the 77 *Klebsiella* spp. reference serotypes strain collection, corresponding to different capsular types (Fig. 4). The obtained cross-infection matrix consisted of 6,622 interactions, with only 3.47% of them (230/6,622) being positive. In general, most phages were highly specific, being able to infect a small amount of hosts (average of 2.67). In addition, hosts tend to be more resistant than permissive, being susceptible to few phages (average of 2.99). Analyzing the modularity (*Q*) of the matrix, the overall modularity (*Q* = 0.604) was slightly higher than the average modularity observed in 600 realizations of the null model (*Q* = 0.572) and similar to the highest of them (*Q* = 0.604). Most positive interactions were found within the modules (180/230, 78.26%). The whole matrix was composed of a total of 17 disjoint elements, sets of phages, and hosts with cross-infections within them but not with other elements (Fig. S3). The elements were not related to viral families or other taxonomic relationships.

Focusing on the diversity of phage host ranges in our phage collection infecting the reference strains, 73 of the 86 isolated phages (84.88%) were able to infect between one and three strains. Nine phages (10.47%) were able to infect between four and nine strains. Only four phages (4.85%) exhibited a broad host range, being able to infect more than 10 strains. In most VC, the host range was consistent across the phages with a standard deviation (std) lower than 1 besides the VC-184-0 (std = 6.94) and the VC-909-0 (std = 3.77).

### Phage cocktail design

Phage cocktails can be an interesting approach to implementing ready-to-use preparations for the prevention or treatment of acute infections. Under this scenario, we designed a cocktail encompassing a combination of 12 phages, as suggested by the Regulatory Medicine and Sanitary Products Agency of Spain as the maximum recommended number of phages for a single cocktail. The cocktail may include lytic phages with different host ranges and distinct types and numbers of RBPs. The overall host range should be broad, targeting more than 50% of the capsular types. Based on these criteria, we decided to include vB_Ko_K5lambda5, vB_Kpn_K7PH164C4, vB_Kpl_K8PH128, vB_Kpn_K50PH164C1, vB_Kpn_K30lambda2.2, vB_Kpn_K34PH164, vB_Kpn_K45PH128C2, vB_Kpl_K54lambda1.1.1, vB_Kpl_K44PH129C1, vB_Kpn_K60PH164C1, vB_Kte_K65PH164, and vB_Ko_K74PH129C2. Firstly, the phage cocktail was tested *in vitro* by serial dilutions of the cocktail using the spot test method in semi-solid media over each of the 77 *Klebsiella* spp. reference strains. Forty-two of them showed susceptibility to the cocktail (54.55%), as shown by the single plaques observed in the dilutions tested. The expected host range of the cocktail, considering the sum of the single phage host range, was 45 strains (58.44%) (Fig. S4). Discrepancies between the expected and observed results were observed for five of the strains. Indeed, *Klebsiella* spp. reference strains of the capsular serotypes 14, 21, 26, and 47 (K14, K21, K26, and K47) were not able to be infected, although it was expected to observe plaques even at low efficiency of plating. In contrast, the strain with capsular serotype 41 (K41) was not expected to be infected, while single plaques were observed during the experiments.

### Carbapenem-resistant clinical isolates representing *K. pneumoniae* high-risk clones

**Fig 5:**
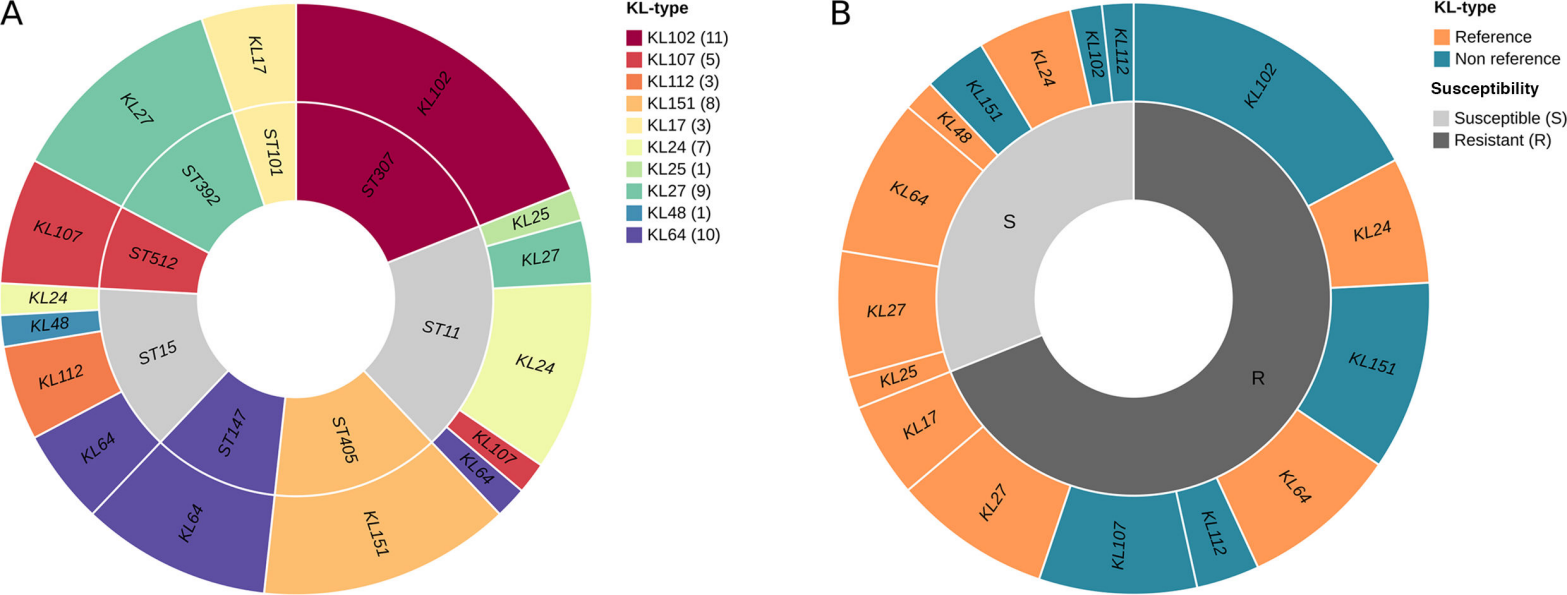
Association between KL, ST, and susceptibility to the phage cocktail in a panel of 58 carbapenem-resistant *K. pneumoniae* clinical isolates. (**A**) Association between KL and ST. The inner circle represented the different STs of the isolates proportionally to the number of isolates with each ST. The outer circle represented the different KLs of the isolates with each ST, also proportionally to the number of isolates with each KL. Colors represent the different KLs. STs colored in gray are not associated with a single KL. The number of strains with each KL type is indicated between parentheses in the legend. (**B**) Association between KL and susceptibility to the phage cocktail. The inner circle represents the proportion of susceptible and resistant isolates, represented in light gray and dark gray, respectively. The outer circle represented the different KLs of the strains susceptible and resistant to the cocktail. Some KLs are present in both categories because there are both resistant and susceptible isolates with these particular KLs. Portions are proportional to the number of isolates resistant or susceptible to each particular KL. Reference KL types are represented in orange and non-reference in blue. 77.8% of susceptible isolates have a reference KL type, while only 22.2% have a non-reference KL type. In contrast, 42.5% of resistant isolates had a reference KL type, while 57.5% had a non-reference KL type.

A collection of 58 carbapenem-resistant *K. pneumoniae* high-risk clones circulating in Spain was created. The genomic analysis showed that the clinical isolates belonged to 10 KL types, including six reference KLs (KL25, KL17, KL24, KL27, KL64, and KL48) and four non-reference KLs (KL102, KL107, KL112, and KL151), and thus are not included as primary hosts during our phage hunting approach. The panel represented eight STs, five O-locus, and four carbapenemase types (OXA-48-like, KPC, VIM, and NDM) (Table S5). The KL type was significantly associated with the ST of each strain (χ^2^ = 315.19; *P* < 0.0001), as previously known due to the clonal expansion of *K. pneumoniae* isolates. Indeed, six of the eight STs represented in our panel were associated with a single KL type. Only ST15 and ST11 were associated with four and five KL types, respectively (Fig. 5A). In addition, a significant association between those parameters and the O-locus (OL) was detected (ST-OL: χ^2^ = 114.81, *P* < 0.0001; KL-OL: χ^2^ = 171.55, *P* < 0.0001). All isolates with ST101-KL17, ST147-KL64, ST11-KL64, ST11-KL24, and ST15 in our panel had OL-type O1/O2v1. Also, all isolates with ST307-KL102 and ST512-KL107 had OL-type O1/O2v2, and all isolates with ST392-KL27 and ST405-KL151 had OL-type O4. The only isolate with OL-type O5 was ST11-KL25, and the only one with OL102 was ST11-KL107. Regarding carbapenemase-encoding genes, *bla*_OXA_ and *bla*_KPC_ were significantly associated with ST, KL, and OL (*bla*_OXA_-ST: Fisher’s exact test *P* < 0.0001; *bla*_OXA_-KL: Fisher’s exact test *P* = 0.0005; *bla*_OXA_-OL: Fisher’s exact test *P* < 0.0001; *bla*_KPC_-ST: Fisher’s exact test *P* < 0.0001; *bla*_KPC_-KL: Fisher’s exact test *P* < 0.0001; *bla*_KPC_-OL: Fisher’s exact test *P* = 0.0072), but it did not occur for *bla*_NDM_ and *bla*_VIM-1_ (*bla*_NDM_-ST: Fisher’s exact test *P* = 0.6257; *bla*_NDM_-KL: Fisher’s exact test *P* = 0.2537; *bla*_NDM_-OL: Fisher’s exact test = 0.1047; *bla*_VIM-1_-ST: Fisher’s exact test *P* = 0.3646; *bla*_VIM-1_-KL: Fisher’s exact test *P* = 0.5690; *bla*_VIM-1_-OL: Fisher’s exact test *P* = 0.1708). All isolates from our panel with ST101-KL17-O1/O2v1 were positive for *bla*_KPC-2_, and all with KL107 for *bla*_KPC-3_ except KL107-ST512-O1/O2v2, which was positive for *bla*_KPC-23_. In contrast, any isolates with ST15, ST405-KL151-O4, or ST11-KL25-O5 were positive for any *bla*_KPC_ gene. In addition, all isolates with ST405-KL151-O4 and ST392-KL27-O4 were positive for *bla*_OXA-48_, and any with ST101-KL107-O1/O2v1, KL17, or ST11-KL25-O5 were positive for any *bla*_OXA_ gene. In addition, anti-phage defense system profiles for each clinical isolate were also evaluated, finding 49 different subtypes. Each isolate presented between 7 and 21 different subtypes of defense systems. CRISPRs were detected in 27 isolates, but spacers did not align with any of the phages present in the cocktail (Table S6).

### Phage cocktail validation in high-risk clones

To study the implications of a phage cocktail in the biocontrol of *K. pneumoniae*, the designed cocktail was assessed *in vitro* against the panel of 58 carbapenem-resistant clinical isolates. The results showed that the cocktail was able to infect 18 of them (31%) (Table S5). To further understand these results, we evaluated the KL types. Only 31 of the 58 had a KL type included in the 77 *Klebsiella* spp. reference serotype collection (reference KL type), and 27 had a KL type not included. Going further, only 14 of the 31 isolates with reference KL types were infected by the cocktail (45.16%), as well as 4 of the 27 isolates with non-reference KL types (14.81%) (Fig. 5B). The results showed that the relationship between having a reference KL type and being infected by the cocktail was significant (χ^2^ = 7.99; *P* = 0.0127). Considering exclusively the 31 isolates with reference KL types, it is worth mentioning that KPN04 (KL25) and KPN19 (KL48) were susceptible to the cocktail, despite reference strains with KL types KL25 and KL48 being resistant to the cocktail. The three KL17 isolates were also resistant to the cocktail, as well as the KL17 reference strain. In addition, 3/7 KL24 (42.9%), 4/9 KL27 (44.4%), and 5/10 KL64 isolates (50%) were susceptible, as were the KL24, KL27, and KL64 reference strains. Regarding non-reference KL types, all the KL107 clinical isolates were resistant to the cocktail, and only 1/11 KL102 (9.1%), 1/3 KL112 (33.3%), and 2/8 KL151 (25%) were susceptible (Table S7). Despite having a KL type included in the reference strains collection that was associated with susceptibility to the cocktail, the KL itself did not seem to affect significantly (Fisher’s exact test *P* = 0.1181), as well as the ST (*P* = 0.1028). OL was neither significantly related to susceptibility (Fisher’s exact test *P* = 0.2390), as well as encoding *bla*_OXA-48-like_, *bla*_NDM_, *bla*_KPC_, or *bla*_VIM_ carbapenemase genes (Fisher’s exact test *P* = 0.7027; Fisher’s exact test *P* = 1.0; Fisher’s exact test *P* = 0.3473; Fisher’s exact test *P* = 0.0550).

The role of anti-phage defense systems in resistance to the cocktail was thus explored. Some of those defense systems may be related to increased resistance to the phage cocktail. However, data were not conclusive enough to establish a significant correlation between resistance to the cocktail and any of the detected defense systems (Fig. S5).

### Targeted phage hunting in clinical isolates and phage characterization

**Fig 6:**
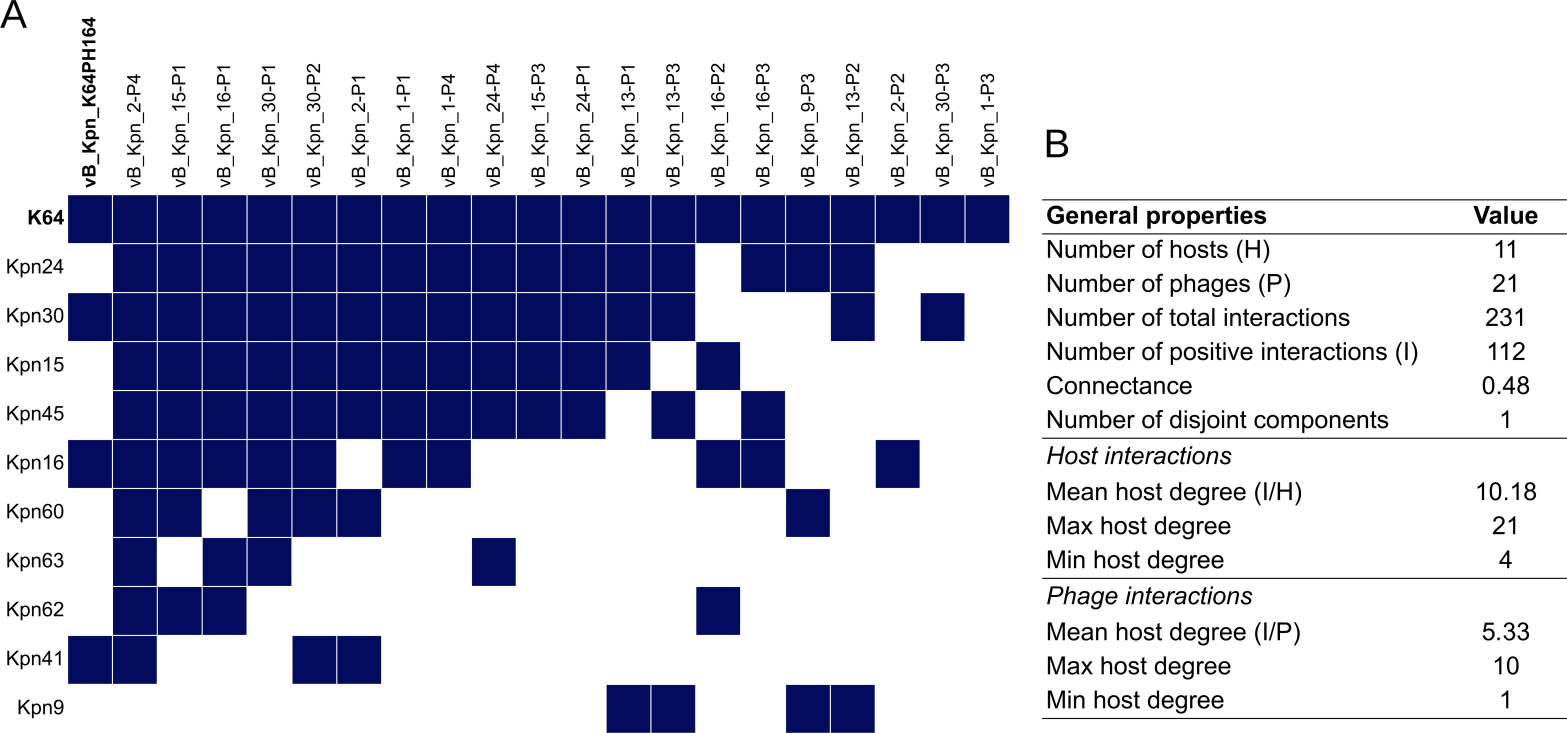
Host range of 21 *Klebsiella* phages and 11 isolates with KL-type 64. (**A**) Cross-infection matrix between 21 *Klebsiella* phages and 10 KL-type 64 clinical isolates and the K64 reference strain (represented in bold). Twenty of the phages were directly isolated in clinical isolates and one in the K64 reference strain (represented in bold). (**B**) General properties of the phage–bacteria cross-infection matrix host interactions and phage interactions.

Given the poor infectivity of our designed phage cocktail against clinical isolates, we decided to explore if phage hunting directed to specific clinical isolates would be a better approach to combat *K. pneumoniae* nosocomial infections. To achieve this goal, we selected KL64 clinical isolates, since this KL type was highly represented in our panel of clinical isolates (10 isolates), but only half of them were susceptible to our phage cocktail. Our bioprospecting allowed us to isolate 20 new phages able to infect KL64 isolates. Seventeen of them were successfully sequenced, phylogenetically classified, and functionally annotated. The genome length of the isolated phages ranged between 39,782 and 114,371 bp. They belonged to five different genera: *Przondovirus* (nine phages), *Sugarlandvirus* (five phages), *Ahphunavirus* (one phage), *Enquatrovirus* (one phage), and *Nonagvirus* (one phage) (Tables S8 and S9). A cross-infection matrix was performed by the spot test including these new 20 phages, phage vB_Kpn_K64PH164 (isolated in the K64 reference strain), and the 11 KL64 bacterial hosts (Fig. 6). A total of 231 interactions were tested in triplicate, with 48% of them being positive (112/231), which is remarkable given the 3.48% of positive interactions in the previous matrix. Phages were able to infect between 1/11 and 10/11 (average of 5.33) different isolates. Hosts were susceptible to an average of 10.18 phages. The most resistant isolate was susceptible to 4/21 phages, and the most permissive was the KL64 reference strain, susceptible to all phages (21/21).

## DISCUSSION

In this study, we have implemented a systematic phage hunt that allowed us to successfully isolate a large collection of new *Klebsiella* phages, showing an invaluable diversity. Previous research has indeed documented the construction of *Klebsiella* phage banks and examined the host range of phages across bacterial collections (28, 47). However, our collection includes well-characterized phages with the potential to infect each of the 77 *Klebsiella* spp. reference strains, thus encompassing all known *K. pneumoniae* capsular serotypes. Our findings revealed an extensive genomic diversity among the isolated phages, as they were categorized into more than 19 distinct VCs, in addition to four outlier phages representing unknown variability that may be representatives of potential new genera. This heightened diversity could be attributed not only to the high number of isolated phages but also to the isolation strategy. By employing a panel of bacteria highly diverse in capsular serotypes, a crucial determinant of phage infection, a more expansive diversity in the isolated phages was unveiled. Our *Klebsiella* phage bank is one of the widest and most diverse collections of genomically characterized phages with known host ranges tested in a capsule diverse strain collection. Thus, it is a valuable resource for personalized phage therapy and a useful data set for the implementation of prediction tools for phage–host interactions using machine learning approaches.

Phage recognition of its host is a crucial step in the infection process, mediated by RBPs, including tail fibers and Dpo (21, 48). By utilizing *Klebsiella* spp. reference strains with diverse capsular types for phage isolation, we captured significant diversity in Dpos. Notably, all phages in our study expressed at least one Dpo. This observation underscores the importance of the bacterial capsule as the primary point of contact in the phage infection process, a finding consistent with previous studies (28). In contrast to previously reported, this phenomenon was also observed among the *Sugarlandvirus*, which exhibited the broadest host range described in *Klebsiella* phages (41). Our results indicate that structural investigations and improved annotation methods can overcome the challenges posed by high diversity in amino acid sequences of Dpos for their identification. While the host range of the isolated phages was diverse, we observed conservation within viral clusters. Phages within the same cluster consistently displayed either broad or narrow host ranges, with the exception of our isolated phages from the genera *Webervirus* and *Sugarlandvirus*. This pattern suggests a potential relationship between phylogenetic grouping and host range specificity.

The high specificity of most *Klebsiella* phages challenges the implementation of phage-based broad-range preparations to fight and prevent infections caused by MDR *K. pneumoniae*. In this work, we explored the possibility of designing a broad-range cocktail able to infect a wide range of *Klebsiella* KL types. The number of phages included in a phage cocktail should be determined by the desired host range of the preparation and also by possible phage–phage interactions (49). The limited number of broad-range phages in our collection forced us to design a phage cocktail to cover the *Klebsiella* spp. reference strain collection. To evaluate the biomedical implications of our phage cocktail, we tested it in a collection of clinical isolates of carbapenem-resistant *K. pneumoniae* circulating in Spanish hospitals. Our panel included representatives for some of the most problematic high-risk clones (ST307, ST11, ST512, ST15, ST147, and ST392), some of them reported worldwide (13). The strong correlation observed of most ST with a single KL type has been also reported in a recent study about the European distribution of carbapenemase-producing *K. pneumoniae* (12). Indeed, ST512 was associated with KL107, and ST101 with KL17, as observed in our panel. This correlation between KL and ST may be due to the impact of local clonal expansions of this pathogen (12). Here, only 31% of clinical isolates were susceptible to the cocktail, and the variable associated with susceptibility was sharing or not sharing a reference KL type, with 5.2% of infections having exclusively reference KL type isolates. Indeed, phages from the cocktail were isolated using *Klebsiella* spp. reference strains, so this association may correlate with previous works, which consider *Klebsiella* KL type as the main determinant for phage tropism (28). However, our results also revealed that even if a reference capsular type is infected by the cocktail, not every single isolate sharing the KL type will be infected. This suggests that, although the KL type would be the primary determinant of host range by conditioning the host recognition step, post-entry mechanisms may contribute to the resistance to the phage cocktail, thereby also limiting the host range (28, 39, 40, 50). In addition, our results evidence the limitations of elaborating broad-range cocktails based on phages isolated from reference strains. Clinical isolates seem to be much more restrictive to phage infection than the reference strains, probably due to their acquisition of more complex and diverse defense systems that avoid the infection of a high proportion of those phages able to target its KL type. In this sense, we explored the possibility of isolating phages directly through the clinical isolates. We suggest that phage hunting directed to specific *K. pneumoniae* clinical isolates is an efficient antimicrobial control strategy and may be the best approach to implement personalized anti-*K. pneumoniae* treatments.

We are aware that our study presents some drawbacks. Preparation of ready-to-use phage cocktails might be an interesting strategy to reduce the bacterial burden, as a preventive tool. However, reference strains have been suggested to be used as primary hosts for phage hunting and phage production, due to the lower levels of toxins and their level of pathogenicity, which facilitates their manipulation. A major limitation in *Klebsiella* spp. is that the reference capsular type collection is based on serotyping, which is limited to a few KL types, excluding a large amount of variability and reducing the possibilities to isolate therapeutic phages. In addition, clinical isolates have acquired post-entry defense systems that can prevent infectivity. However, we can solve this issue by isolating phage directly through the clinical bacterium, as suggested here using our systematic approach. Interestingly, and thanks to the permissiveness of the reference strains, we can use them to amplify and produce the phages for therapeutic purposes. As for our collection of clinical isolates, it includes a reduced number of isolates, which may bias our descriptive statistical analyses, but it includes high-risk clones that have been identified as representing the most problematic ST and KL in *K. pneumoni*ae clinical infections (12, 13). Another relevant limitation is the evaluation of our experimental results by spot tests. These assays present qualitative data, which sometimes have discrepancies compared with other techniques like liquid infections (28). Despite the constraints mentioned, this work represents the larger collection of fully characterized *Klebsiella* phages and the first evaluation of a broad-range phage cocktail designed based on the phage–host interactions encompassing all the reference KL types.

### Conclusions

*K. pneumoniae* is a highly variable encapsulated bacterium, representing a major challenge for designing phage-based products to prevent or treat pathogenic infections. Our results demonstrate the potential to isolate phages for any high-risk clone, suggesting the interest in developing phage hunting directed to specific *Klebsiella* spp. isolates as a faster and more efficient way to implement phage therapeutics as personalized tools. This would allow the design of adapted phage cocktails based on the epidemiology of the region, with a few phages targeting the clones that cause the majority of infections. In addition, phage production and scalability could be achieved using reference strains, which are more permissive and easier to manipulate. Our results are a step forward for new phage-based strategies to control *K. pneumoniae* infections, highlighting the importance of understanding phage–host interactions to design rational cocktails as personalized treatments against *Klebsiella* spp.

## MATERIALS AND METHODS

### Bacterial strains and sequencing

The 77 *Klebsiella* spp. reference strains collection, corresponding to the *Klebsiella* spp. reference serotypes, was purchased from the Statens Serum Institut (Copenhagen, Denmark). In addition, 58 carbapenem-resistant *K. pneumoniae* clinical isolates corresponding to the high-risk clones circulating in Spain between 2019 and 2021 were selected for this study (Table S5). They were all collected from clinical samples (urine, blood, surgical devices, and diverse swabs and exudates) in Spanish hospitals and stored by the *Programa Integral de Prevención y Control de las Infecciones Relacionadas con la Asistencia Sanitaria y Uso Apropiado de los Antimicrobianos*, at Instituto de Biomedicina de Sevilla (Sevilla, Spain), and CARB-ES-19 project (13), and *Programa de Vigilancia del Laboratorio de Referencia e Investigación en Resistencia a Antibióticos e Infecciones Relacionadas con la Asistencia Sanitaria* at Centro Nacional de Microbiología (Instituto de Salud Carlos III, Spain). Sequencing data for 33 isolates were previously available at the European Nucleotide Archive (ENA) PRJEB53686, PRJEB52486, and PRJEB50822 and National Center for Biotechnology Information PRJNA1040118. The other 25 were sequenced for this study (ENA: PRJEB68301 and PRJEB70314; Table S5). Genomic DNA libraries were prepared using the Nextera DNA Flex Library Preparation Kit (Illumina Inc., San Diego, CA, United States), and whole-genome sequencing was performed using Illumina NextSeq 550 according to the manufacturer’s instructions, generating paired-end (2 × 150) reads. The quality of the reads was assessed using FASTQC (v0.11.9) (51). After *de nov*o genome assembly using Unicycler (v0.4.8) (52), quality was assessed with QUAST (v5.2.0) (53), and ST, K-locus (KL), O-Antigen (O-Ag), and carbapenemase-encoding genes were identified using Kleborate (v2.3.2) (12) using the CARD database for the detection of anti-microbial resistance genes (54).

### Phage isolation, purification, and DNA extraction

A high-throughput screening of environmental samples was performed for targeted phage hunting against the 77 reference capsular-type strains. Phages were isolated from environmental samples collected from wastewater treatment plants and surrounding areas in Valencia (Spain). Once plaques were observed in a strain, they were purified by several plaque assays, as has been described before (26). The isolation of phages in the *K. pneumoniae* reference strain with capsule serotype 20 (K20) was performed differently due to technical issues. In this case, prior enrichment of the environmental samples was needed, incubating the samples with the host overnight (37°C, 200 rpm). After 24 hours, lysates were centrifuged twice (3,900 rpm, 5 min) to remove bacteria. For phage concentration, all phages were amplified for 3 hours in a final volume of 5 mL in Luria–Bertani with CaCl_2_ 3.78 mM (37°C, 200 rpm) and centrifuged twice after the amplification (3,900 rpm, 5 min) to remove bacteria. Lysates were centrifuged in a high-speed centrifuge (80,000 rpm, 3 hours), and pellets were resuspended in 200 µL of SM buffer [50  mM Tris-HCl pH 7.5, 8 mM MgSO_4_, 100 mM NaCl, and 0.01% gelatin (wt/vol)]. Removal of host DNA and digestion of phage capsids were done in two consecutive steps of incubation with DNAse and benzonase, as described before (28). Extraction and purification of DNA were performed using the DNA Clean & Concentrator 5-Kit (Zymo) or Maxwell PureFood GMO and Authentication Kit (Promega) with the Maxwell RSC Instrument (Promega).

### Phage sequencing and assembly

Sequencing libraries were prepared using the Illumina Nextera XT DNA kit (paired-end reads 2 × 250 bp or 2 × 150 bp), and reads were generated in the Illumina MiSeq platform with iSeq Reagent Kit v2. For potential contaminant analysis, the Kraken 2 tool was used (55), while sequencing read quality was assessed using FastQC software (version 0.11.9, Babraham Bioinformatics) (50). *De novo* genome assembly was carried out using either Unicycler (v0.4.8) or SPAdes (version 3.13.0) (56). SPAdes includes a step for read error correction and quality-trimming of Illumina reads, adding an extra layer of quality control. Various kmer lengths including 21, 33, 55, 77, and 127 were explored along with the “careful’ mode during SPAdes assembly to ensure high-quality assembly. The final genome assemblies were subjected to quality verification through QUAST (53) software to ensure reliable and accurate assembly. This tool integrates confidence scores to enhance the accuracy of the classification. Genomes of sequenced phage are available in ENA PRJEB56576 and GeneBank under codes PP848836-PP848838, PP848840-PP848847, and PP848850-PP848855 (Table S1).

### Phage clustering and taxonomic analysis

Full phage genomes were clustered using vConTACT2 (56), capable of replicating the genus-level viral taxonomy assignments from the International Committee on Taxonomy of Viruses with 96% accuracy. Blastn (46) was employed to investigate intergenomic similarities within each VC, further delineating relationships between the isolated phages. Phylogenetic analysis was conducted for each VC, constructing neighbor-joining trees based on intergenomic similarity matrices to illustrate the genetic relationships between the phages.

### Phage characterization and genomic organization

The lifestyle of the phages was predicted using the tool BACPHILIP (57), searching for the presence of protein domains associated with the temperate lifestyle in the phage genome. The process of gene calling was carried out through the consensus call of multiple programs, namely, Phanotate, Prodigal, Glimmer, and Genemarks, via the integration tool multiPhATE (58). The tRNA genes were specifically identified using tRNAscan (59). Default parameters for gene calling allowed three potential start codons (ATG, GTG, and TTG) and three termination codons (TAA, TAG, and TGA). A minimum open reading frame (ORF) length was set to 90 nucleotides. The identified genes were annotated with the PHROG (43) and PDB (44) databases using Hidden Markov Model (HMM) profiles.

### Comparative genomics of the *Klebsiella* phages

The genome plot comparison was constructed with the R package gggenomes (60). The pairwise comparisons of all the nucleotide sequences were conducted using the Genome-BLAST Distance Phylogeny method (61) under settings recommended for prokaryotic viruses. The resulting intergenomic distances were used to infer a balanced minimum evolution tree via FastME (62) including subtree pruning and regrafting post-processing. Branch support was inferred from 100 pseudo-bootstrap (SPB) replicates each. Trees were rooted at the midpoint and visualized with FigTree (63). Taxon boundaries at the species, genus, and family levels were estimated with the OPTSIL program (64), the recommended clustering thresholds, and an *F*-value (fraction of links required for cluster fusion) of 0.5. The phylogenetic tree of each VC was computed from the similarity matrix using the neighbor-joining algorithm. The resulting trees were represented using iTOL (65). Multiple sequence alignments were constructed using Clustal Omega (66).

### Genetic analysis of phage host recognition

Special attention was dedicated to the genes involved in host recognition. Protein sequences with a size inferior to 200 amino acids were discarded. Initially, the tool PhageRBPdetect (67) was employed to identify RBPs (method: ProtTransBert + HMMs + XGBoost), which integrates embedding representations computed through the ProtTrans language model with the identification of the presence of domains associated with RBPs. Next, Dpo domains were further investigated in the genomes. Despite their conserved structural conformation, namely, right-handed β-helix, 6-bladed β-propeller, and triple-helix, their amino acid sequence is highly diverse. This prompted us to employ approaches based on structural features (33, 68). Two distinct methods were employed. Firstly, proteins whose annotation fell into either RBP, structural, or unknown were scanned against a local database of HMM profiles derived from Interproscan 5 (69) entries related to the Enzyme Commission (EC) numbers EC4.2.2. and EC3.2.1, indicative of carbohydrate lysis activity. Proteins that presented a significant match over a minimum of 50 amino acids were selected, and their 3D structural conformation was predicted using the AI model EsmFold (70). The presence of the Dpo domain was subsequently confirmed by scanning the predicted 3D structure of the protein against a local Dpo database using Foldseek (71). The precise sequence of the Dpo domain was inferred after predicting the domain boundaries using SWORD2 (72). Secondly, DepoScope (73), a deep learning tool tailored to the identification and domain delineation of the Dpo, was used. In some cases, additional insights were gained by analyzing the genomic organization of phages. The conserved synteny among phages from the same family facilitated the identification of potential Dpo candidates that were missed by either method. This comparative genomic approach complemented our other analytical methods and helped refine our predictions. To validate the annotations from each method, we predicted the 3D structures of the identified sequences using EsmFold and manually verified the presence of Dpo domains. Finally, to characterize functional domains and their organization within the identified Dpos proteins, the FoldSeek webserver (https://search.foldseek.com/search) was employed to scan their predicted 3D structures against hosted databases.

### Phage host tropism and infectivity

The *Klebsiella* phages were assayed by the spot test against the *Klebsiella* spp. strains to evaluate their host range in triplicate. Drops of 1 µL of each phage with a titer after its conservation at −70°C > 10^5^ PFU/mL were added to bacterial lawns using the soft agar technique (74). Plaques were incubated at 37°C overnight. Strains were considered susceptible to a phage if they showed a clear or turbid spot for at least two of the three replicates. In addition, to evaluate infectivity and phage production, spot tests using serial dilutions were performed, using 1:10 and 1:10^3^ dilutions of each phage. In this experiment, single plaques in at least one of the dilutions were considered positive.

### Analysis of the cross-infection matrix

The obtained cross-infection matrix was bipartite, because it contains two types of elements (phages and bacterial hosts) that interact. It can be decomposed into disjoint elements, composed of phages and bacterial hosts that have cross-infections between them but not with other elements (75). Disjoint components of the cross-infection matrix were identified and plotted using the R package “igraph” (76). The modularity of the matrix was analyzed, and the modules were plotted with the R package “bipartite” (77). The statistical significance of the modularity was tested utilizing null models obtained with “bipartite.” Six hundred random matrices were used to calculate the significance of our obtained modularity.

### Phage cocktail design and *in vitro* validation

A phage cocktail was created by combining phages with a diverse host range covering the KL type of at least 45 reference strains, including KLs of some high-risk clones frequently identified in Spanish hospitals causing infections (13). A combination of 12 phages of 10^7^ PFU/mL each was used as a phage cocktail. After its conservation at −70°C, the cocktail was tested *in vitro* by spot tests against the *Klebsiella* spp. reference strains as previously described and against a panel of 58 carbapenem-resistant *K. pneumoniae* clinical isolates. In addition, analyses of the association between susceptibility to the phage cocktail and other qualitative variables in clinical isolates were performed. The correlation between different qualitative variables of the clinical isolates (susceptibility to the cocktail, KL, ST, O-Ag, and carbapenemase-producing genes) was studied with Pearson’s χ^2^ analysis or Fisher’s exact test depending on expected frequencies using the R commander package (78).

### Anti-phage defense analysis of clinical isolates

Although phage recognition and phage adsorption are key for infectivity, post-entry mechanisms are also important for successful phage replication and bacterial lysis. To evaluate the possible correlation of anti-phage defense systems with resistance to the phage cocktail, anti-phage defense mechanisms were identified using defense-finder (39) from the sequences of the clinical isolates used in this study. CRISPR-Cas systems were detected using CRISPRCasTyper (79). Spacers were compared with phages from the cocktail using blastn (80). Differential probability of resistance (dPR) was calculated as the difference between the number of isolates with a defense mechanism (DF) that are resistant to the cocktail (R) and the number of isolates with this DF that are susceptible to the cocktail (S): dPR = P(R|DF) − P(S|DF). To avoid the effect of the association of some defense systems with a particular KL, this was calculated separately for clinical isolates with potentially susceptible KL, in which at least one clinical isolate with this particular KL was susceptible to the cocktail but others were resistant. Values close to 1 were considered as possibly related to resistance to the cocktail.

## Data Availability

The short-read sequence data of bacterial isolates sequenced for this study are available in the European Nucleotide Archive under ENA PRJEB68301 and PRJEB70314. The sequences of the isolated phages are available in The European Nucleotide Archive under ENA PRJEB56576 (phages isolated in the 77 *Klebsiella* reference strains) and in GeneBank under codes PP848836-PP848838, PP848840-PP848847, and PP848850-PP848855 (phages isolated in *K. pneumoniae* KL64 clinical isolates).

## SUPPLEMENTAL MATERIAL

Supplemental figures and table spectrum.00254-24-s0001.docx Fig. S1-S5; Table S7.

Tables S8 and S9 spectrum.00254-24-s0002.xlsx Table S8: *Klebsiella* phages isolated in *K. pneumoniae* clinical isolates. Table S9: Functional annotation of *Klebsiella* phages.

Supplemental tables spectrum.00254-24-s0003.xlsx Tables S1, S3, S5, and S6.

Table S2 spectrum.00254-24-s0004.xlsx Table S2: Functional annotation of *Klebsiella* phages.

Table S4 spectrum.00254-24-s0005.docx Table S4: Amino acid sequences of detected depolymerase proteins.

ASM does not own the copyrights to Supplemental Material that may be linked to, or accessed through, an article. The authors have granted ASM a non-exclusive, world-wide license to publish the Supplemental Material files. Please contact the corresponding author directly for reuse.
