# Supplementary material for: Targeted phage hunting to specific Klebsiella pneumoniae clinical isolates is an efficient antibiotic resistance and infection control strategy
Source: Microbiol Spectr. 2024 Aug 28;12(10):e00254-24. doi: 10.1128/spectrum.00254-24 (PMC11448410; doi:10.1128/spectrum.00254-24)
Supplement: Table S4 — Amino acid sequences of detected depolymerase proteins. [file spectrum.00254-24-s0005.docx]

**Table S4. Amino acid sequences of detected depolymerase proteins. All of them carry a polysaccharides-degrading domain.**

>K10PH82C1 cds 45, tail depolymerase

MAIASPLVYCLDNHKEDHMALVSQSIKNLKGGVSQQPDILRYPEQGAKQVNGWSSETEGLQKRPPVVLKKVLGDAGYLGSDPMVHLINRDENEQYYVVFTGTSIRVFALDGTERTVRGDFSYVTTGNPQKDLRMITVADYTFVVNTTKTVEVNEGSLSNGGNFQDNVDGLINVRGGQYGRKLVVTINGTTVEHQLPSGSNATEDPPKVDAQAIAEALAVLLRAALPSWTFDVGTGYIHVQAPSGTTINEFITKDGYADQLISPVTHYAQSFSKLPLNAPDGYLVKIVGDTSKTADQYYVKYDKTQKVWKETIGWNVTTLLHFHTMPWALVRAADGQFDFNWHNWEPRKAGDDMTSPHPSLVGSQITDVFFWRNRLGFLSGENVVLSRTGRYFDLYPPSVANLSDDDPIDVAISHNRVSILKYAVPFAEELLLWSDEAQFVMSASGVLSAKSVELNLTTQFDVQDGARPYGIGRNIYFASKRATYSSVYRYYAVADVSEVKNAEDITTHIPQYIPNGIFSINGSGTENFCSVLTKGAPNKIFTYKFLYIDEQIRQQAWSHWEFPEDVQVQAADSIGSTMFLLMKNHTHSWLGQVDFTKDTTDLPNEPYRLYLDLKKEYTIPSAAYNWDTNETTVTLSSVFGMTLGGNSKLSMVEEDGKVTTWDTYGSTVRMIGDYSGKHVFFGVHIPFTYEFSKFLIKKTDEYGSTSTEDSGRLQLRRAWVNYEDSGQFTVSVQNLSRTFKYDMGGNRIGSDKLVLGELNLGTGQYRFPVVGNARVNTVTIYSEAPTPLNIIGCGWEGNYTRRTSGI

>K10PH82C1 cds 49, lytic tail fiber protein

MAKYDKSIPSEYDALFQKAADSHGVSYDLLRKLAFNESSFNPAAKSPTGPLGIMQFTKGTGAGMGLKITGGPDDERLNPALAIDAGARHLSDLVRKYNGDELKAALAYNQGEGPNGAPQIQAYDKGDWASISEEGRNYMRKLLDVAKSPQSGALEAFGGITPKGKGIPSDSAFAGIGTKSKVGDALPESTGFNVKGTAQPEPAVPYAKSFWEATGTTIDEFESRSTFFGFKDAAAAELQNSTLGVAVRAGRADNGFDVFKDTITPTRWNSHSWSPEELERIRNEVKNPNYINVVTGGSPENLDALIKLANENYEADQKASGAGLGAKLSAGVIGAGVDPLSYVPLVGVAGKGLKVVNKAFVVGAQAGALNVVSEALRTSIAGGEAHYADAALGGMLFGSGMSVLTDAVSRGLRKAGGTEIPNEFAGPALRLEARETALNTGGADMTLMPTEGRVFDKEHAGVPYADHPVESGAVILNNGAILSDTNPLNPRTLQEFSEINPERAAPGIKLGGFTEIGLKTLRSESPEIRSLASDLVRSPTGMESGTSGKFGATASDIHERLHSTDQRTYNQLYDAVREAMRDPEFSTGDMKLSREGIRQEIYKRAALAIERPELQASLTKGERKVMDIMKAHFDTKRELMENPGMFGRMDAQSIFPGSRHKGTYVPNVYDRSAKLAMVNKLGNDGLQEAIALSWLTSYRSRPEVKARVDEYLMELNGLKTVQEVTPEMVRKHAMDKAYGISHSDQFTQSSVIDDNITGLVGIENNNFLEARNMFDSDMPITLPDGSTFSVNDLRDFDMFRVMPAYDRRINGDIAIMGGTGKTTKELKDAIMALDKKSEGKGTMKGEVEALKDTVKILTGRARRNQDTVFETALRSLNDLAFFAKNFYMGPQNLTEISGMLAKGNVSAMLHGIPFMNDLATRTAPLKGSELKELHGIIFGKELDQLIRPGREDIAQRLREASDTGVMMSEAVGTLKYATQELAARSPWTRMLNGTANYLMDAARQGVLGDVAGAALGGKSSKFGKENFLKSASVSADQWKGIQQLFKDYAVRNEDGSFTIKDKKAFSQDPRTMDLWRLADKVADETMLRPHKVSQQNSKAYGAGMNMAMQFKNFTIKSLNAKFIRSFYEGYKNNRAIDMALTHILSLGIAGSYYVAQAHVKASALQDHQQKDYLKRALNPTMIGYAALSRSSHLGAPLGIATMLGGALGFQDMQMLRSTILPRAEEQKGRGDKAANSRSVAADLAGNIALQVPALGYVGSLGSTAINAYGVLTAPNKPTERDFMTGLMNSTRELVPNDPLTQQLILKIYEANGVRIKEPAKPN

>K10PH82C1 cds 50, tail spike protein

MAITVRTVTTFPLDDAQRDFNINFDYLARKFVQVAVIDSTLSKPRKELVLGTDFRFTTKTSLNTTIAWGPNDGYDRIEIRRVTSTTERVVDFADGSILRATDLNASQVQSIHIAEEARDAALLAMPQDDNGNLDARNRRIVRLAPGIDGADAINKNQLDQTLGEAGGILSDMDALAKLVQEYIANFINDPASVRNVNFVYNGGAAMGGEQSFKIIRPEPTLGVPYIYINGDRQDVGYQYSFDQPTQTVTLVKPLVAGDFVVAVTAEGSIPFLDLIAGATGAASIGKAGGGTVQDGLDMSLHGTDVHKDTVKAGTVDQILAINQVNNGRDLRGKEFVMPHGTFYASRTLRTRGLSAAEPKYTTFSWKGQGSTFTKLIHPATGGPGAGDIAYMDYLKDVEMSGFSMDNTPLGAGTSSTNTKNGQFWIRHSEDSRFDDLRFAGGDALTFCLDHCKNIISTDLKVDYQLRYPVGTGKSPLIVGDYSEQCMFIGGYVKTVSPDGSIMYSGDLADNDQANDTKWAFINLYGLTFEQKPNSNACMWQEGEGAPSNAHFIGMNYVNNGIGHGVSEKAVGTDLGSTFRQAQVRAIWNRAEYISIGGHFLDNDAKYPAGTGGAGAAATGAVHNDNAKFTSLVGDYFRGNAADYTDYTGATARNPENSAHITNAKLTSLIRTAASSNSQHLAIVNSQLTDPARISGGGNGRLHISLIGSHCIGPLGNFGHGQSETQLDAIGSTFTAAGSTETLITQSGVGNISFSKCVIREYVSLTAGNSGRVSFDNVTFDSVTFTEADLSARYINCKFINCVNSPDTNGNHFMADSTNRPSSARTTVTLGAGGTYQFPAWVTQDRGVYQVSVGGNGANLPAAMGIIGRASATGTGVWTPQFESTQGSVVVSWAPGGRITVTVTAAGNYTIGIN

>K10PH82C1 cds 51, tail spike protein

MLTNLNQPKGSTIGVLRDGRTIQEAFDSIDSSILNLMYSVVLYPTGDIVKDTQAKDAAIAKCEDSGKDLIIAAGSYKWSSGIEWNPRRYSIHGMGDVIVDVSGIPAGSYAVTLKTVHNGTKLWNNISKVMERIVFVGPATVNGIKIDSVADNSKVMNKFSNLYFNGFNIGVYLGNNQWLNRFEHCGWRNTTNKIYINAIEDSGENIQFVGCDFFGQNATLDDVCINHAAGNIEITFDGCSFDFMTSTLYAKWDQGRGIMRFSKCHFESRGQQDFEVDSANSWYHLILDGCDWTWDSSTMPTRIGRINSTNVYRPACLTVTNCNFNSGKAFDLGGRELFTVLGQSVIRQSGNNVISEKGNRGVIISRANRRTPFMDMASYTAGTIYNTGSGTVSESAEFPTSNTLFGNALAVNGPKSIWTKTPVVGGTRICFAGNVKRGNPSVGVQLRFYDTADNVVSVASTQTDGTTGDWQKFCQQYVVPYNAVGVRMQLDCSMQAVGEVTTIHSWMVESHS

>K11PH164C1 cds 39, tail depolymerase

MALVSQSIKNLKGGISQQPEILRYPEQGSLQVNGWSSETEGLQKRPPMVFIKSLGPRGYLGEDPYIHLINRDEYEQYYAVFTGNDVRVFDLSGYEYQVRGDRSYISVANPKDNLRMITVADYTFIVNRTRQVRENQNVTNGGTFRDNVDGIVNVRGGQYGRKLEVNINGVWVSHQLPPGDNAKEDPPKVDAQAIAAALADLLRVAHPTWTFNVGTGYIHCIAPAGVTLDEFQTRDGYADQLINPVTHYVQSFSKLPLNAPDGYTVKIVGDTSKTADQYYVKYDASQKVWKETVGWNISVGLEYHTMPWTLVRAADGNFDLGYHEWRDRRAGDDDTNPQPSFVNSTITDVFFFRNRLGFISGENIVLSRTSKYFEFYPPSVANYTDDDPLDVAVSHNRVSVLKYAVSFAEELLLWSDEAQFVLSANGVLSAKTAQLDLTTQFDVSDRARPYGIGRNIYYASPRSSFTSIMRYYAVQDVSSVKNAEDMTAHVPNYIPNGVYSINGSGTENFACVLTKGAPSKVFIYKFLYMDENIRQQSWSHWDFGDGVEVMAANCINSTMYLLMRNAYNVWIAAVDFKKESTDFPFEPYRFHVDAKRSYHISETAYDIETNQTVVNVKDIYGASFSKGTVAICESDGKITEYEPMGDSWDSTPDIRISGDISGKDIVIGFLYDFRYVFSRFLIKQEQNDGTTSTEDSGRLQLRRAWVNYQDTGAFTVSVENGNREFNYLVNARVGSTGLRLGQKATTTGQYRFPVTGNALYQKVSLSSFNASPVSIIGCGWEGNYSRRANGI

>K11PH164C1 cds 45, tail spike protein

MDQDIKTVIQYPVGATEFDIPFDYLSRKFVRVSLVADDNRRLLSNITEYRYVSKTRVKLLVETTGFDRVEIRRFTSASERVVDFSDGSVLRAADLNVSQLQSAHIAEEARDSALLAMPQDDAGNLDARNRRIVRLAPGVEGTDAINKNQLDTTLGEAGGILSEIKQTEKDIQDYIENFADDTTSLKGINWVYNNGSANGGETSILITREGPVFAVPTIYINGDRQSVGYHYSYDSGDKTIHLVKPLEAGDFVECVTSEGVLPLSNLLSTPDGASQIGTKSGLTVQDYLNGVKSATILRNIEPVIDGQRIVLSEISPTLGPKSGGTLVYDQSDTSSVDDGYTVFVTAGGKRWKREESYIDVAWFGPNFGLALQTAVNLVDNYVRTVGFYSRKTIYVAAGNYTTDRQIDIPSYVSVVAIGNVNINGASLPVNSYVIRITNKVTGISTTQHSGWNLGSVGGTLRLVGNGNANQVDGLFVGNTTAMSDVRNVSLYAVATSGVRYGLTFGSTNTYLFTATKCHFETSLVNLYFPYTTSANSGEKMVFNDTVFGGATRNHVEMSTPGMDLTFNNCSFDFTGGSIIYGTETWGYSKVGLNSCHFEGFDNLWVKVDAPQGGFIGSNRAITIANATVLPRRRSNTTGTNSPSRMHIDAKSTPVYISGLDLRHEVVPYTEEIFMTSPETTLMLQGYLKDPYFQIPNKSYIQNRGWDITDETTGTLVNSSATMDALTRFTCIERNALSAEVVEGGTSGKLLAMTGAGGYFTLVTKGFIPVSTFQRIGGAMSIQAAASTGNIQCTIGVQWFDYDGNLIGTDQAFAINMREVFNNSSLPNFSEGNNRFISTSTRTFRAPAGAAKCKPLWRISGHTGVVNISRLASFVL

>K11PH164C1 cds 46, tail spike protein

MLNDFNQPKGSTIGVLKDGRTIQQAFDEIPVISVLRFGAKGDGVADDYPAFQRAALEAQRIGGAVIDVPTPPVEYKIGFPVFLFDRTWFRGTGINCRVNFTDPLYARKSRSGFIIGSGYEQNRDKAIQCLNDGTWATTGSVVNSAFTELPRGQYVRDNPSQVQSRLCRVSDMYLVATYPNGTTLKGGYAVSGANAVDSDVFNIWGEGWTEIINFGSDVPPATPSCHNMHAYDITCVEPNHYETYYSAGFMANSTNCTIGRFRQLKPIADGSPHGSGGSMNYTEFCSFYDIDIPSLGRTASSEGILVNNSKGAVTRNIRIGNAKTCVAEYYTTGAGIFYDRDHPNVFDGIHANNCDNAAALRSKFSVWKNVTQTNCTYHVYFGTTNAQSCVVKFVPDSIGFGSGVDGLARLRDNRVNGYIERSVYVRPINFLLEDKTVLQSWDTNRNMKAKPDVGFRVLYPIPVNMRAIVSVNQFFTFEVGAGSKGSNVDIKVRRMASFGGNASEQPIIEFSNSKTATVDTVQDTNVQANAPALVLTATPDMPNSMDVLITVSNPTINMNLKEFRLVYLGD

>K12P1_1 cds 43, tail depolymerase

MALISQSTKNLKGGMSQQPDILRFPEQGALQLNGWSSETEGLQKRPPMLFNRTVGEAHSMGTAPFIHLINRDEFEQYYVVFTGADVRVFNMEGGEYQVRGDRSYVFTNNPRNDLRMVTVADYTFIVNRNVVVEEHPDMTNGGTFNENRDFLINVRGGQYGRTLQVGFNGGIFATYKIPDGDQKEHVQNTDAQWLAEELARQMRANLPAWTFNVGQGYIHCVADPGNSAWGIWTKDGYADQLINPVTHYAQSFAKLPPNAPDGYMVKIVGDTSKTADQYYVRYDNNRKVWAETVGWNQKFQMVDTTMPWTLIRASDGNFDLKPASWGKRTAGDLDTSPWPSFVGSKLNDVFFFRNRLGFLSGENVVLSRTSKYFNFFAPSVSNFSDDDPIDVAVSHNRVSTLKYAVPFTEELLLWSDEAQFVLSASGILSGKSVELNLTTQFDVQDKARPFGIGRNIYFASPRATFTSINRYYAVQDVSSVKNAEDITAHVPSLIPNGVFSISGSSAENFAAVLTSGAKGRVYIYKFLYLDEELRQQSWSYWDFGDNVEVLAANTIGSRMYLLLRNQFHAWSCEINFTKESIDFENEPYRIHLDMKRQYVIPANAFNEDTFQTALNIYDMYGSNFQKGDITIVELDGKITTVSPPPGGWNTGHWFYLDGNLAGKLVFCGFNFEFRYEFSKFLIKKAADDGSTATEDIGRLQLRRAWINYEDSGAFTIRVENLSRVFEYDMAGGRLGSDNLRVGRLNLGTGQYRFPVTGNAKYNKVSLLSDNTTPLNVIGCGWEGNYIRRSNGI

>K13PH07C1L cds 10, tail spike protein

LSVTRLTSSLVRWKSKLANAIQRSLSSKMEESLSVLDFGAVADYNPTTKTGTDNTQAFRNAVAAAIAQNIRNVYAPGGPSAYMTTGEINLGGEGFTGGEGSRDVWRGITQGVHFFGDGPYSTIIAFNPPNTDAPCFSARGGWGTHSPRALSKLAIEPVNWADYNATSSGTGVLLQGCCFVPVTDVHIGRFHRGIHFWNKLQGTDDPTNTFTKGDFTEFNRITRVRVFNCDIDIDYQVSLGNNSFHGNSFTDCMCQINSYGGIGMRMWDDGSRNAIRPSSLPYEYIANVYNNKHEINWFGSDARTCYLMHIDKAQGHGCNGDMTVEAAVTLRAIGQYWYQSFGSLHSISAINTVVDGDTDTATRPVAFMWMNSAYPQVNFDGTDPLLTSGLTPRQYDLNNSGNTGMELLNIRGANTGAIWSIQNGAALGWILGRRAQADSRKGTRSVWQFSYNGEVIKSVSAANVGLQNSTGAGFGMLGDTLLRPYAASTISLGSPTYPFTRLRTTDWTVDTNGIVPVQDGIKNIGSSSLRVGTVFAATGTINTSDARLKTEVRPFTSEEILAAKALSEEIGMYQWVASIESKGDNAREHCGLTVQRAIEVMQQHNLDPFNYGFICHDVWDEVVEVSDETGDVISSTPAGDRYSFRFDQLSLFISRGIEARMSELESRM

>K13PH07C1L cds 11, tail spike protein

MPIIKGGYMALVKATFVKDVDGQPWRFSSVAKMKAFNYSCYLGSSVFLESWHEGAGLGSGLFKVSKGTTEVGDDGSVIVADDGTRLIRVFDGPIFADMWGALPSSTYNSLPAIKSAYLYASSKLQQLFLGGGSYKVTGSSGIDIDPSLAGISALSRARVDATEFTGDYLFTITSSYSYTPAPYYNNLSVALEGLYVFGNKTEGRSGLLTGRRTTDGVKSYNGQTEIRNCTFDKFDYNIRMGHNSWRIVFYKVNSLNALNANGILYVPSGLDDSGEILTFYHCQFFDMAHCILQGKQPECTERQRDTLRP

>K13PH07C1L cds 12, tail spike protein

MIVVRFLLSITVSSLTWRIVFYKVNSLNALNANGILYVPSGLDDSGEILTFYHCQFFDGAGSNIRISCSSFAMTFVSCSFLNITFTIDAGSSVSVTAMGCNFENPGSQNTRRYIEITAGHTNIFNVVGGSIVTNANAGQTQALIHVSANNQINLSNLTIPYGAHYQQEADSGYHAFCSGQGYVSTSNCSLQLLNGAGCCPIHPSLSVFTNWNLSYANLNAWTVNKGSAPTSVAEYLSAQGPKGEGVLHVAPTTQGVNISQVATVSKQAGSMSMSVMVNIISASANAGQISLAYLDAFDNNLGGVSANLGTTTGWKVIGKNTLRGRLPIGTAKVRLNIQTVAGADVQYTNILCNII

>K13PH07C1L cds 54, tail depolymerase

MEVQGSLGRQIQGISQQPASVRLPGQCTDAINCSMDVVEGTKSRPGTVHIARLGDLGLIQDNTNIHHYRRGDDVEEYWMITNPLGIPDIFDKQGRKCTVTETEGAASYFNSNNPRVDYKFFTVGDTTFVVNRTKIVRARADKTPAVGGTALVFSAYGQYGTNYQIIINGVKAAEYKTASGGSASDVETIRTEVIAEQLYTNLLTWAGASDYSISRMGTTIVISSLSGASFTVDTEDGSKGKDLVAIQYKVTSTDLLPSKAPVGYLVQVWPTGSKPESRYWLKAETADGNLVTWQETLGADEVLGFDGTTMPYIIERTNIVGGIAQFTIKQGYWDDRAVGDELTNPMPSFIDQSLSDIFMVQNRLCLAAGESCIMSRTSYFFQFFRQTVLSAVDTDPIDVFADASEVYALKHAKVLDGDAVLFSDNAQFILPGDKPLTKATALLRPTTTFEVDTNVAPVVTGEAVMFATKDGAYSNIREFYTDSYSDTKKAQPVTSHVNKLIRGGIYHMASSTNFNRLFALSEDNRSRVFVYDWLWQGTDKVQSAWHKWEFYGATIGGLYYSGETLYLIIKRNDGVFLEAMYMGDPLLSGSDQVRMDRTVTVSLTWDEATLSWKSSPLPWVPTQVEMLEAVLTNGDPAYVGGAFLFEYNPSTRVISTKYGLGDTSQIWAAKVGQMYKVEFVPTDVIIRDSQDRVSYQDVPVIGLVHLNLDRYPDFTVEITNRKSGAVRVAKASNRVGGARNNAVGYVKPTSGTFSFPLRALSTDVEYRIISISPHTFQLRDIEWSGSYNPTRKRV

>K13PH07C1S cds 10, tail spike protein

LSVTRLTSSLVRWKSKLANAIQRSLSSKMEESLSVLDFGAVADYNPTTKTGTDNTQAFRNAVAAAIAQNIRNVYAPGGPSAYMTTGEINLGGEGFTGGEGSRDVWRGITQGVHFFGDGPYSTIIAFNPPNTDAPCFSARGGWGTHSPRALSKLAIEPVNWADYNATSSGTGVLLQGCCFVPVTDVHIGRFHRGIHFWNKLQGTDDPTNTFTKGDFTEFNRITRVRVFNCDIDIDYQVSLGNNSFHGNSFTDCMCQINSYGGIGMRMWDDGSRNAIRPSSLPYEYIANVYNNKHEINWFGSDARTCYLMHIDKAQGHGCNGDMTVEAAVTLRAIGQYWYQSFGSLHSISAINTVVDGDTDTATRPVAFMWMNSAYPQVNFDGTDPLLTSGLTPRQYDLNNSGNTGMELLNIRGANTGAIWSIQNGAALGWILGRRAQADSRKGTRSVWQFSYNGEVIKSVSAANVGLQNSTGAGFGMLGDTLLRPYAASTISLGSPTYPFTRLRTTDWTVDTNGIVPVQDGIKNIGSSSLRVGTVFAATGTINTSDARLKTEVRPFTSEEILAAKALSEEIGMYQWVASIESKGDNAREHCGLTVQRAIEVMQQHNLDPFNYGFICHDVWDEVVEVSDETGDVISSTPAGDRYSFRFDQLSLFISRGIEARMSELESRM

>K13PH07C1S cds 11, tail spike protein

MPIIKGGYMALVKATFVKDVDGQPWRFSSVAKMKAFNYSCYLGSSVFLESWHEGAGLGSGLFKVSKGTTEVGDDGSVIVADDGTRLIRVFDGPIFADMWGALPSSTYNSLPAIKSAYLYASSKLQQLFLGGGSYKVTGSSGIDIDPSLAGISALSRARVDATEFTGDYLFTITSSYSYTPAPYYNNLSVALEGLYVFGNKTEGRSGLLTGRRTTDGVKSYNGQTEIRNCTFDKFDYNIRMGHNSWRIVFYKVNSLNALNANGILYVPSGLDDSGEILTFYHCQFFDGAGSNIRISCSSFAMTFVSCSFLNITFTIDAGSSVSVTAMGCNFENPGSQNTRRYIEITAGHTNIFNVVGGSIVTNANAGQTQALIHVSANNQINLSNLTIPYGAHYQQEADSGYHAFCSGQGYVSTSNCSLQLLNGAGCCPIHPSLSVFTNWNLSYANLNAWTVNKGSAPTSVAEYLSAQGPKGEGVLHVAPTTQGVNISQVATVSKQAGSMSMSVMVNIISASANAGQISLAYLDAFDNNLGGVSANLGTTTGWKVIGKNTLRGRLPIGTAKVRLNIQTVAGADVQYTNILCNII

>K13PH07C1S cds 53, tail depolymerase

MEVQGSLGRQIQGISQQPASVRLPGQCTDAINCSMDVVEGTKSRPGTVHIARLGDLGLIQDNTNIHHYRRGDDVEEYWMITNPLGIPDIFDKQGRKCTVTETEGAASYFNSNNPRVDYKFFTVGDTTFVVNRTKIVRARADKTPAVGGTALVFSAYGQYGTNYQIIINGVKAAEYKTASGGSASDVETIRTEVIAEQLYTNLLTWAGASDYSISRMGTTIVISSLSGASFTVDTEDGSKGKDLVAIQYKVTSTDLLPSKAPVGYLVQVWPTGSKPESRYWLKAETADGNLVTWQETLGADEVLGFDGTTMPYIIERTNIVGGIAQFTIKQGYWDDRAVGDELTNPMPSFIDQSLSDIFMVQNRLCLAAGESCIMSRTSYFFQFFRQTVLSAVDTDPIDVFADASEVYALKHAKVLDGDTVLFSDNAQFILPGDKPLTKATALLRPTTTFEVDTNVAPVVTGEAVMFATKDGAYSNIREFYTDSYSDTKKAQPVTSHVNKLIRGGIYHMASSTNFNRLFALSEDNRSRVFVYDWLWQGTDKVQSAWHKWEFYGATIGGLYYSGETLYLIIKRNDGVFLEAMYMGDPLLSGSDQVRMDRTVTVSLTWDEATLSWKSSPLPWVPTQVEMLEAVLTNGDPAYVGGAFLFEYNPSTRVISTKYGLGDTSQIWAAKVGQMYKVEFVPTDVIIRDSQDRVSYQDVPVIGLVHLNLDRYPDFTVEITNRKSGAVRVAKASNRVGGARNNAVGYVKPTSGTFSFPLRALSTDVEYRIISISPHTFQLRDIEWSGSYNPTRKRV

>K14PH164C1 cds 24, tail spike protein

MLLSALLNQPMRLKNMALYREGKAAMAADGTVTGTGTKWQSSLSLIRPGATIVFLSSPIQMAVVNKVVSDTEIKAITTKGAVVASTDYAILLSDSLTVDGLAQDVAETLRYYQSQETEIADAVDFFKSFDFEALQSLADQVMADSEAAGASASAAAASEVEAKTSETNAKASEVAAESARDQVQQIINDAGEQSTLVALAQPDGAKKVHLKHGGNVQDAVDFITFDMWSDTIDKTGETSVLDKMIAFFAYASANNLKIKQSGGKYLLSGTGNIVFSVPYEFTDGCEFIIDNWNGYFLYSNGNGQQMTYNADNESTYGVLAAVNSSADLGKGTSRLSGLSGDARFSDHAVFMESSKPWYYARGKLYNWHNLNRISNFGYLDAPFMYDSGGAITSIYALPVAKRFSTLKGLQLALRNVTALYVLRIEHSSLVKIEEVTVSEKPETLPKSFAMISTDYVYGLHTADIRDKYPNNTVINGSAAFGYMLNLNHTFEVVVERAKGEGKGWGVTNGTFTGRVTYRDCVLNRIDTHNPVHEYLHIDNCVLGRSSISVVTMGNMRIRDCIINETDEAGGHLVSTRGDLGGWSAGTLEISNLTIGGKVTDRTNISWNFDYLVLGFNDTSTGTTGVVAGSGISNRLFDKIIIDGIEADADSSGLAPFTKVFDNKNPYRIGCPELVSIRNVEWDYDTPLTIDYKNFLDPDGTRESTAIHPVLAKPTTRLVMENITAREIKINGEAYYHTPDVVMKNIKPPVGKDGVKLTLAQRGRYSIDDFDFSQVVAAGDGNVTPNSLNGRISLKMRNGRFFNSETATNGGLLGNALLITGSHDQSEIDVENSEIAIALSGISDRTNNIALAKAARISNCIFWDTITGARYVGLPLGVGGSGATSISLNIKLKVGNRFAVITATGYADVFVPPFPGERNIVDTTGTFKVAFAVDSDKKSATIGNVTAPIAITAMYIL

>K15PH90 cds 49, tail depolymerase

MAQSLEGTIQSLLQGVSQQVPRERQPGQLGAQLNMLSDPVSGIRRRPPGEIVWESTIDNPGLDSLFTEYVERGTDGRHLLINTSNGNWWLLAKNGKTILNSGNDPYFVTTVGQTSLQTASIAGLTYILNTEMAPNTTVDNTGRIDPSTTGFFYVKSAAFQKRWNVTVTSAGVDYSGDYTAPAAGSTSGNAEEVSGAYVAQQLRDSLVANGLPAGNVSVRGAYLFFYGLSNCVVSSDAGDTYAVVSNQSRVDQEQDLPAQLPAEADGAMCRVGTASSETAWYQFDYSTRTWSEVGAYGSITKITNMPRELAADDNIIARDWEGRLAGNDDNNSDPGFVENGYITGIAAFQGRLVLLSGSSVDMSASGLYQRFYRSTVTSLLDTDRISISSASAQDSVYRTAVQFNRDLVLFANSMQAVVPGSAVLTPTNASISITSTYDCDSRVTPVMAGQTVIYPNKRNDSYAGILELIPSPYTAAQYTTQDATVHLPRYIPGRVLQMQNSSVTNMAFSRMSGERNSLLVYEFMWGGNDGAKMQAAWHKWSFPYPILSVQALEDEVFLYMQGPGPDNKLLIVSMDPREGYQLGSEYREAYSDLQKQVQVQDGVFTVPSVLRPVGWADNYKEELILTYLPSNPMGPTEVGIKEIAGENTLRVVRGVPDGTYVIGRRYRSTFTLTTPILRDQNDKLVGSGHVRLLRLDVAVRNSGHFDVQVLDTPRDVNWGGELTGILMNSKELTLGQALRMDLATITVPCRTNADTTEVSLFTDGSMELNVLDISYILRYNQRRRRI

>K15PH90 cds 54, Putative tail depolymerase

MINMAYSWQEQVKPAGTQDIQCDIEYLDKSYIHVYLDGTETTAFTWTSSTNIRLNSPLSAETVVLLIRKTEREYLYIAFASGAPFIEGNVDTQNTQFLHLAQELVEGRAIDGFYGDINMHQYRITNLGHPSQAQDAATKGYVDSEIASINDAFRAGDASLDKKIDDRFSRTLRVGESSIPELPPLPSLEGRLLAISGGKPVGVLPESGSASDVMVELAKPDGYTNIGGFFSAEGAESVGDTNQSVWERIYKRGEFEIHQMPGDPAGVAGIVFGGPNNKAALVIDERGRIQTYAVVDGKLSAAQVGTPIVQGTGYFNNGFEDRIIRFPSAGGESDDLTPGILITTNANGICDFFVVSPNQFNDLGYPTGSSIDKQMFKLVKSLDIGTSLQRTIDQYPTVENSSPVMYRLFDVNYSGFGSKVVSGILSVGNYVDYTSNVYLVTLNPQNLSAAPSLGPDNIYQWLKVTALHGGQSGWGDSSLTPAVGLVNDPTNSLAKIFLRLPSYGQRISFTPINVANPSVVTFYYNDLRNNVTVDTPNYLVYSFAEKMATDRFYVRMNNGDVRYSPGTVVRVSGNVTDRLSDKLSDGVFAIGGAYHLANKGRASSIAVTNPSTGQYVISGCNLRADSWKVCPPVGFSGTSFGTPPYVVNIVSQGTNTFTIEVRTAAGSLVNINGFDWLDLHVQPV

>K15PH90 cds 55, tail spike protein

MQYLYVDPSDDVNKPVPSAFKTVTAAIAAITSYPFSILIRRGTVLKEAVNARLVNTSSDMSFFDTYGEGPPPKWLSTSNSAGQLASWNARRITVQNIHFMDRDDGFTSSATSVNLQCSGDASGLADVHVKNCYFTGNAYSVSNPTKNTVGRHVQLFAVAYDNAATPAHKLSVEDCMFNMVNAGVYIRGNTYAADTTDNRGDGSRTYGAKVLRCSFTNVVQEGVLMHACTSKNDVYTDDEWQSCVRDCYYSSYRWDKFTTSGVSTYDAPFWMWHCNRVLFEKLEVHGSYPMAPDNMAVDIDGMCWDNVVRYVYSTGCARTVMFVSADNAGLQAAPPAGMSNFEFYYTRRQGSGNNVVEYCFAFNDGIQRLQGQVGTPTNDACSISHHRNQFNNTVRNCVFIDTASVRQRRLISTNGYDTESVSVPSLTVESCIFYCKWMQPSSIIAQLSTGSRTIAPASQIVLNNNIIWSDAWSAAPDLSALATLSGNIWAKPQYALLPPSPPSTRRGAVNLGIPANAKGAGVSNLLLDIKGHSGNNIGWLQ

>K16PH164C3 cds 43, tail depolymerase

VESSDVNQKLKEDQMALVSQSIKNLKGGISQQPEILRYPEQGSIQVNGWSSETEGLQKRPPMVFIKSLGDRGYLGEDPYIHLINRDEYEQYYAVFTGNDVRVFDLSGYEYQVRGDRSYVTVNNPKDNLRMVTVADYTFIVNRTRQVRENQNVTNNGTFRDNVDGIINVRGGQYGRKLEVNINGVWVSHQLPPGDNAKDDPPKVDAQAIAEALATLLRTAHPTWTFNVGTGFIHCIAPANTTLDVFETKDGYADQLINPVTHYVQSFSKLPLNAPDGYIVKIVGDTSKTADQYYVKYDASQKVWKETVGWNISVGLEYHTMPWTLVRASDGNFDLGYHEWKDRRAGDDDTNPQPSFVNSTITDVFFFRNRLGFISGENIVMSRTSKYFEFYPPSVANYTDDDPLDVAVSHNRVSVLKYAVSFAEELLLWSDEAQFVLSANGVLSAKTAQLDLTTQFDVSDRARPYGIGRNIYYASPRSSFTSIMRYYAVQDVSSVKNVEDMTAHVPNYIPNGVYSINGSGTENFACVLTKGAPSKVFIYKFLYMDENIRQQSWSHWDFGDGVEVVAANCINSTMYLLMRNAYNVWIAAVDFKKNSTDFPFEPYRFHVDAKRSYHISETAYDIETNQTVVNVKDIYGASFSKGTVAICESDGKITTYEPMGDSWNSTPDIRISGDIAGKDIVIGFLYDFQYVFSRFLIKQEQNDGTTSTEDAGRLQLRRAWVNYQDTGSFTVSVDNGSREFNYLVNARVGSTGLRLGQKAATTGQYRFPVTGNALYQKVSLSSFNASPVSIIGCGWEGNYSRRANGI

>K16PH164C3 cds 48, tail spike protein

MDQDIKTVIQYPVGATEFDIPFDYLSRKFVRVSLVTDDNRRLLSNITEYRYVSKTRVKLLVATTGFDRVEIRRFTSASERIVDFSDGSVLRASDLNVSQLQSAHIAEEARDAALMAMPQDDAGNLDARNRRIVRLAPGIDSTDAINKNQLDTTLGEAGGILSDMKDLEGELHDYIEKFADDTALVRGVAWVYNLGSANGGENVVTIDKPTRAYAVPYIAINGARQEVGYHYQFDINTQSISLVKPLEKGDFLMAMTTESSVPLESILASPIGASGIGTLNGMTVQEAFEQLPYVPIEQFGAKGDGVTDDTDAVQAALASGKNLSLSGTYLVKSTLSITKDGQKLIGAGGVLKVDVGAVTLLKATGVSNVSITGVSLQSSSMETNTYNGACIWAENCSGWVISGCLFSNYKASALFLNKSSNFKVVHNTFKGAAGAAGDVTMWRSCSGNVVSENTMMSGSDTAIVLQTIEDADISVDNIISYNRIANCTRYGIVVYNSLERKTGSLLRTRIIGNSVDTIYGSVTNPSAGNSKTYGAGIYVLSAEGVIVSGNSVSRCNLDTNSSTLSPGCIGVNATSNAVISNNVVMAGGYHGIFVSDALQQGAGNGAGSAAYRPIGGVVISGNTATQCVRDGIHIENKHSVKLIGNTCTHNSGNGVLVSTTSGGTNYPALVDISVEATSSLRNGLAGLSLSNCQSAQVINGSFSWNTGDGVSTGSPASVLANLLIVGNSRGIVMSASAVDCSVTLCRVSSNSAGLVAFSPYLFNNNIIKGNTTDFNGNYGPVKNLTGSATPSVKFVKVITLGDSTTITDLLDGELGQTVVIRAAANGTTVVHSTNGIRLNGSVNFVMQAGDTLTLTKFFQSQWDEVSRMTRAVVTS

>K17alfa61 cds 23, tail spike protein

MALYREGKAALSADGTVTGTGTKWQSSLSLIRPGATIMFLSSPIQMAVVNKVVSDTEIKAITTNGAVVASSDYAILLSDSLTVDGLAQDVAETLRYYQSQETEIADAVEFFKGFDFESLQNLANQIKADSEAAESSAAAAAASVIESKASEANSKASEVAAETARDQVQQIINDAGDQSTLVVLAQPDGYSKVGGLVERWQEEERQVIYAVNHGIIESGAVDSSDKLQNLIDSAGSAAIILPKIIGLKKTVKYRTNSCILGVPGSFTQVNMMSGFVGVSAFEPISKERNGAISPVIKYLTITDVTAVSVGAGTDSEKNGVDITGTFDADISDIKGVRIKNTVYSSPGTELQHTRRPKINALDGSNVDRHILLEGSGTGRFAYGDVFVSMFKTTGNCLKQSVIESADGLQVDGGVIFPNGGLRISGHYLTLTNVHLFEPKAELGSDEVPAGIHIKRMSPTSPSKNVTIAAVDIAFPGRLANTTIGNPAQVNNPGPGLIMEGVEDFNIQVNINNSSMQSAHLIDCANGVLSLTSREANTQQLGSGALPPGTYDALFMEGCADVHVMLVDNSASRKYAVNLDDNCKGCTVNGSVSKGYVIGQDVKIPNNQSNRVQLSIGNGASGLTRYQYDSTPPMPRYVSSEDGNTTPSVTGGVNNTVVKFQNGAVTNVTDLPDVTNSMEVTVNINDNNATRLVDVANGGKFKTRLGANIMGKGTYARFIRDAATGYLVQL

>K17alfa62 cds 64, tail spike protein

MNKMFTQPVGPVAKQVNRQTIARIFELPINQVGYISPFQAIDDYEVLFDPISQLCWWRDDAVGTPVSWGVSGSTLTLNTETAGTFLLKQAKCWKELQDILASATGAYGVNLRKNYSGAALRSLAAIVEETISPFDFDDIYSDAVYDPTTGLVSGSDSTSGMQRMLNEAHAHGANIEFPLNGRFATKSLYLHYDADLNPDWEGRPGRIQLSGGVTGHATGDVETQGTAIFHIPGEATPLISMKGEFSLTNPSAMGGYLSLSRLNLVGSKDSSDVLLLQGSQGQIELFDYTVKVINPNGSGITEATTWETMHRNGLIRGGASTAAPGVWKGIGLDLKSDGTQGQINMKIYENVNVYRMGTGIDIGRKYEAQGTFGPLVFIGGQTSLSDEYGMRLGGGVIAFTSLGQQHEGARKNGIRIDRTLADGTLESDLPRSIKIQQTYITGCGTIEDDSVDSYAIYVANGDGIEIDSPTFNTVGNGIAFDSGVVDDLLIRRPTFRTVRTYGTASGYGIRAFGEQNASKRHQLVDPVFNQNPATPIDTVAREIFGRGAVGGRISLATNLATPSISMGSSAGGTSFKQLNFNYSVPTTVTNILGGNTHQLLLVTFSNTNATLEHGTNIILQGGKNVQGSTNKVIQLYYNGTVWREIGDPVRALTGDSANRPYSTAFAGMMYFDTTLNKPIWRNAANTAWVDASGTVV

>K17alfa62 cds 66, tail spike protein

MTRYNGCGCGGHVHSGCNPCTSHNEIQQAVNDALALEKENLEQYETNAAQSATDAAKEAADAASSASAAAQSQANAETAAGTATQAAASVTDTAVVLEETAERIEQAQDLLEEQISALQTKPVYFEVSTPTSSLVLPDTETVFNVRSIYVASARQDVGYGFTFDKATRTITLTDEITAEDLAGAEGGFILVTVICDVYSSDDPTSFPLIMASNAGATNVGTSTGATVEARLTTLDSQVDPTLRPNLASDEEGLGADLVNTVLDVSVGKYIQNSSPRISDYKVTKGADATLAFAKAFAKAAETTGVVIVDVSCVVDTIAVPSETTIRIADYKTVTHKDNASAHMFILPSDSLIKANNKKGRLRGNSANQSAIRRCISAVGVSNAGVDGVDIRDFNSYGAYFENCEDTFCKNSTIRDITGGAVETAAGQYVTNCLRHESAFNDIRDTGSNGIKFRADTLGQTQGCSSTNDKVYRAGFIGIANGKCKDHTVTNAYCESCVDNGCDMNGCYNTRFIDCVSVACQDGFYMGENGIDNCHIINPTARNCKRSGVGSLGSLTRCSVINPTIDACGSGIYCSGFVGFKIRDGSITNSVKQTYTDNETGTVKTSTGNGIDLQSNLSGCYQPDISGTTFYGNAGYDIAFGSSGTIGNLMLGECAFQDSTGDGKISYGGATLADPQIRNSQGYIVSRTQAYNLAGDGTTLDFTLSLPQAVADANYRIVSVVPDWLTTVRYLDSTKGTSYFGISFGSAPPSGTRRVNISFERLRPV

>K18PH07C1 cds 231, chaperone dependant tail spike

MADTTQFEQAVDQVIEDSERLHKVVNGSAIDTVIVEDGSTIPTLRKALLDNVYFKTPPQPWVAGTQATVFNQLYSFTNSAGTFWWYAPGASPSTPITLPADPSTSTAWKVYNDSVVVSEKFAPLNTPAFVGSPTAPTPAQGSNSGAIATTAFVNLAVAAAINSLAGSSPSYAALTVVGASTLNTLVVSGTSQFGGTLDASGVLGKFQKISLSGQTATLSFDYTAASNYLKTIISPNSVQTNNLTSAVIVNGTASADNTTMSLTGVGNNTFDYVYIRGNSSKASTEPRLKVTGTTELENVRVTGSLSGVNVGVDGLDILPNSIITTTTAEIGSDLTVNGITTLGSTTIQDLGVVSSLTVNGNSTLTGGFTAGSASSVAGNLSITGLLAVTGEATVSTNLTVTGNANLNNGGTGTTTVNNLNVLGTLTGVSIDVNGKDINPNSVLATTTIEAYGSLKGASLVITGEATAPKVTANFVGTTPGSVTTPSAGTTWTPGGTGSDLTKMNNIYNVDVTNTLTIGPWANLGSAFTATIYLFQDATGGHAVTLDASYKIINGGAISTTANSVTILQVTYCGRGTVYDVAVYQRP

>K18PH07C1 cds 243, tail spike protein

VVKCTNVKKRGKYKMLQSVKISELPSASTLTEDDLIVVDQPDNTKKATLFQVFSHLEDTVEQSVLVELAQPTGASKIGLSQGGNLQQSVMYFTPDQDGAVGDGVNDDSDHVQNIFNKAAAAAIASPSGIFIPQTVKINRPYRITKTLTLDGSKVRVEGDAGGCLYFDPAGTYTNSRGIVVTNNANLAAFIGNAKPLFSGISFLTTGNTLDLFYAVRGDAVSPNNGACLHHITSCVFRGFNRLFTHGAGGWGWVWMACTATNTQFWLYLTSEADTYERHSFFGCQWGSGGTALYINNTNGKVYWEAGSVDYCTSIATVAAGHFSMSGHYEFTGRTSSLINILGSNASVSLEGMWAIVGNTSTKWYLVNQFQDYQLSIDKLTVIHDGNNIASGLFSNKKFRKGYIYFPSDAAKLMGYFSSDTELLLDSSGYFDFSLSNSAVTTASISGGQLRYTGVGGSGTTSYLYVDIPIHGASQISFMLTASYTSTIGPMVMTKQLLTSGKTVITTLTEGTVSWPVGSSSVAGGMRTIKEIPPGASFLRLIFNGFYLNSTTSFNIESIKLWRA

>K18PH07C1 cds 245, tail spike protein

MSVTQTWRTGVPTPGVLRTYPPNPLYVDTVRGSAAGTGSLNNPVNSLSLALGLCAGLPDYVIKIAAPETNPLRQEVIFDTSMNVFLEGIDNEPWHIYGSEMHTSGWVLSGNIYSKVINYTSVMQVVVTTMTETIADKDDFYLKLVQNTTTPTTPGLGQYGYSGSTIYVRLPDDSDPNTHQIEVSRRNFGVGTVGFGLLTVRDVVSRYCMINGISCGLSTQPAGTGFLTVYDSVVEYCANGGVGGTGRNERIECTNVKAYRISNDGFGQHAPTGGAGLMILNGCDGSYNGDAPGQSAQGASNHEATTMILNGGTFNHNVSGGMVVIESGTCDIHGDTEYGPVVMDGNMRLGNTAGTIANQAGCAWLDNAKGTVTGSVTVKNGLGIGVKRASTAIVDGISNIISENNAFPDQL

>K19PH14C4P1 cds 43, tail depolymerase

MSLINQSIKNLKGGISQQPDILRFAEQGSVQINGWSSESEGLQKRPPMIHLKTLGPAGYVGAQPYVHLINRDEFEQYFVVFTGEDIKVFDLDGKEYQVRGDRSYVRTANPREDLRMVTVADYTFVTNRKVVVQSNDQSVNLPGFKDQGDALINVRGGQYGRRLSIEFNGSERAAVQLPDGSQPAHVNEVDGQAIAEKLAAQLRNNLGNPNNDQDPNKWRFNVGPGFIHILAPNNDNVWGLQTKDGYADQLINPVTHYTQSFQKLPINAPDGYIVKIVGDTSKTADQYYVRFDLNRKVWVETIGWNTRTHLHYHTMPWALVRASDGNFDFKYLEWGARTVGDDTTNPYPSFTGQTINDIFFFRNRLGFLSGENIILSRTSKYFNFFPASVSNYSDDDPIDVAVSHNRVSTLKYAVPFSEELLLWSDQAQFVLTASGILSSRSVELNLTTQFDVQDRARPHGVGRNVYFASPRASFTSINRYYAVQDVSSVKNAEDMTAHVPNYIPNGVFSISGTTAENFAAILTSGAPNRVYIYKFLYIDEEIRQQSWSHWDFGDNVTVFAAQVINSTMTVLMSNEHAVWMGRLHFTKDSIDLPGEPYRLYIDAKRKYTIPAGTYNDDTYQTSISLSTIYGMNFTKGKVSVVFPDGKIVEIDQPIHGWGSDPILRLDGNQEGQVVYIGFNIPFTYTFSKFLIKKTAEDGSTATEDIGRLQLRRAWVNYEDSGAFIIRVNNLSREFIYTMAGARLGSDNLRVGGSNIGTGQYRFPVVGNAQTNIVTIESDASTPLNIIGCGWEGNYLRRSSGI

>K19PH14C4P1 cds 48, chaperone dependant tail spike

MLIYKGGHMATTIKTVMTYPLDGSTTDFNIPFEYLARKFVRVTLIGVDRKELILNQDYRFATKTTISTTRALGPADGYNLIEIRRYTSATERLVDFTDGSILRAYDLNISQVQTLHVAEEARDLTADTIGVNNDGDLDARGRRIVNVADAQDVGDAINLGQIQRWNDSALNSANRAKQEADRATARASEAATSAGNSASSASLSRDWAIKESPVEGTFKSSRSYALDSMAYRDASKSSADASAASAGAAKTSEINAKNSEIATKTSETNAKTSETNAKASEVRAIEEANKLENMNDLAGAIKEVLKPEGDTSGKVVWKYDIDSKVLRAYNGMSIGWDWDGTGSPFMNFYRGQGEPSGSLRLDANKVVRFNGMNSVDFDSTINAQAINGSWIHSTGDLVADATLYVHGGDYIYKDKWNPSGANKVTNLIRGSVGGFACDEFFSEIVGYYIERGWHLVGGDNDTWMRIKGNGNFEIAGNRGARIVINGGAIVEQDGNIRGSIWEGKWLRDFMNDRFLNDVWLGGQEWVFTNGGARIDYALGGGSVCTGYTQDATGGVRNAWLDGMFHRKLWIRYGNGSARVIGSA

>K1PH164C1 cds 8, tail spike protein

MALIRLVAPERVFSDLASMVAYPNFQVQDKITLLGSAGGDFTFTTTASVVDNGTVFAVPGGYLLRKFVGPAYSSWFSNWAGIVTFMSAPNRHLVVDTVLQATSVLNIKSNSTLGFTDTGRILPDAAVARQVLNIIGSAPSVFVPLAADAAAGSKVITVAAGALSAVKGTYLYLRSNKLCDGGPNTYGVKISQIRKVVGVSTSGGVTSIRLDKALHYNYYLSDAAEVGIPTMVENVTLVSPYINEFGYDDLNRFFTIGISANFAADLHIQDGVIIGNKRPGASDIEGRSAIKFNNCVDSTVKGTCFYNIGWYGVEVLGCSEDTEVHDIHAMDVRHAISLNWQSTADGDKWGEPIEFLGVNCEAYNTTQAGFDTHDIGKRVKFVRCVSYDSADDGFQARTNGVEYLNCRAYRAAMDGFASNTGVAFPIYRECLAYDNVRSGFNCSYGGGYVYDCEAHGSQNGVRINGGRVKGGRYTRNSSSHIFVTKDVAETAQTSLEIDGVSMRYDGTGRAVYFHGTMGIDPTLVSMSNNDMTGHGLFWALLSGYTVQPTPPRMSRNLLDDTGIRGVATLVAGEATVNARVRGNFGSVANSFKWVSEVKLTRLTFPSSAGALTVTSVAQNQDVPTPNPDLNSFVIRSSNAADVSQVAWEVYL

>K1PH164C1 cds 53, tail depolymerase

MAQSLEGTIQSLLQGVSQQVPRERQPGQLGAQLNMLSDPVSGIRRRPPGEIVWESTIDNPGLDSLFTEYVERGTDGRHLLINTSNGNWWLLAKNGKTVVNSGNDPYFVTTVGQTSLQTASIAGLTYILNTEMSPVTAVDNTGRIDPSTTGFFYVKSAAFQKRWNVTVTSAGMDYSGDYTAPAAGSTSGNAEEVSGAYVAQQLRDSLVANGLPAGNVSVRGAYLFFYGLSNCVVSSDAGDTYAVVSNQSRVDQEQDLPAQLPAEADGAMCRVGTASSETAWYQFSYSTRTWSEVGAYDSITKITNMPRELAADDNIIARDWEGRLAGNDDNNSNPGFVENGYITGIAAFQGRLVLLSGSSVDMSASGLYQRFYRSTVTSLLDTDRISISSASAQDSVYRTAVQFNRDLVLFANSMQAVVPGSAVLTPTNASISITSTYDCDSRVTPVMAGQTVIYPNKRNDSYAGILELIPSPYTAAQYTTQDATVHLPRYIPGRVLQMQNSSVTNMAFSRMSGERNSLLVYEFMWGGSDGAKMQAAWHKWSFPYPILSVQALEDEVFLYMQGPSPSNKLLIVSMDPREGYQLGSEYREAYSDLQKQVQVQGGVFTVPSVLRPVGWADNYKEELILTYLPSNPMGPTEVGIKEIAGENTLRVVRGVPDGIYVIGRRYRSTFTLTTPILRDQNDKLVGSGHVRLLRLDVAVRNSGHFDVQVLDTPRDVNWGGELTGILMNSKELTLGQALRMDLATITVPCRTNADTTEVSLFTEGSMELNVLDISYILRYNQRRRRI

>K2064PH2 cds 25, chaperone dependant tail spike

MALYREGKAAMAADGTVTGTGTKWQSSLSLIRPGATIMFLSSPIQMAVVNKVVSDTEIKAITTKGAVVASSDYAILLSDSLTVDGLAQDVAETLRYYQSQETVIADAVEFFKDFDFEALQNIANQVKADSEAAGASAAAAAASEGAAKTSETNSKASEQAALTARQQAGSARDQTQSLYNQTVDLVAGVQAPDKLPTSPGSSRAWMKIANVKNAGQSNCFAQFIIGGGANFGSANLPVDIFSISGRGLPDTLTSNNIDANFTQCALIAAANSTSRLKLGVVKNTDGSFDVYLLSPNGYFPVMWLNRLNVQSNNGLITGPIIDRTGYSWITTEPAGIVYNSPSDYLMANDSTIPRTNVANTFSQPQAISVPGGNATLTLNGAVVRANNNNAIVYSIPVDGQGMYFRPNGDMNNAKQVVFDAANFTVTGLNATFSNAVTMLSTLRVNGASNLSGGVDVTASQKLPLKETTATTGIGVNFIGNSATECSFGIENTAGGSAVFHNYTRGASNSVTKNNQLLGGYGSRPWLGSDYTAHSNAALHFLGAGDASARNNGGWIRLLVTPKGKTISDRVPAFRLSDNGDLWLVPDGAMHSDFGLVRSFETLNAAVPKFNAPTNQDGRGLKIVADGAPEINMIAPRGSNTSSPAVRAMWCDGSLGNSDKYIGAVQQWSTFFFGASGHDGQKFDSMRGAVSIQATEGWGKTSTPTRILFETCAAGSTTRTSRWCVDHNGNFIPMGDGGYDIGWGSGRVNNIYAKNGAINTSDGRMKNDVRAMNDPETEAAKAIAKEIGFWTWKEQADMNDVREHCGLTVQRAMEIMESFGLEPFKYGFICYDKWDEKTVVSEYGPANEDGSENPIYKTIPAGDRYSFRIDELNMFIAKGFEARLSALEDKLGM

>K2069PH1 cds 25, chaperone dependant tail spike

MALYREGKAAMAADGTVTGTGTKWQSSLSLIRPGATIMFLSSPIQMAVVNKVVSDTEIKAITTKGAVVASSDYAILLSDSLTVDGLAQDVAETLRYYQSQETVIADAVDFFKDFDFETLQNLANQVRADSEAAGASASAAAASEGAAKTSETNAKTSEQAALTARQQAGSARDQTQSLYNQTVDLVAGVQAPDKLPTSPGGSRAWMNIAKVKNTGSGFAFAQFIIGGSSGYGAANVPVDIFSLSGRGLPASPLTSDNIDIWFTQRSLVAARPSAPRINLGVVKNTDNSFDVYLHAPSGYIPEMWLNRLNVQPRGDVITGPIIDRTGYSWITTEPAGIVYNSPSDYLMANDSTIPRTNVANTFSQPQAISVPGGNATLTLNGCVVRANNNNAIVYSIPESGQGMYFRPNGDMNSAKQVVFDASNFTVTGLNASFSNAVTMSSTLRVNGSSNLRGGVDVTASQGLPLKETTATTGIGVNFIGSNTTECSFGIENTAGGSAVFHNYARGASNSATKNNQLLGGYGSRPWLGSYYTEHSNAALHFLGAGDASESNNGGWIRLLVTPQGKNIGWRSPVMRISDNGDTWLATAGSMVSPDLDGVRSFETLNAAVPKFNAPTNQDGRGLKIVADGAPEINMIAPRGSNASSPAIRAMWCDGSLANSDKYIGATQAWSNFFFGASGHDGEKFDSMRGAVNIQAPGGWGATSTPTRILFETCATGSTTRTSRWCVDHNGNFIPMGDGGYDIGWGSGRVNNIYAKNGAINTSDGRMKNDVRAMSDPETEAAKAIAKEIGFWTWKEQADMNDVREHCGLTVQRAMEIMESFGLEPFKYGFICYDKWDEQTVVSEYGPANEDGSENPIYKTIPAGDRYSFRIDELNMFIAKGFEARLSALEDKLGM

>K21lambda1 cds 28, tail depolymerase

MADLSISIISDQASESNQAGWWHPLDSFQGVEYYGLCKEYGTVDYHQVEIVRRDADGTLTRGMCKNVDGTVAEFKNDVGHNQPSVVVDGAGYIHVFTSMHVNLLRYFRSARPGDVSQMVDATLDFPDVDWVWTYPITGRGPDGDAYCMMRVASRSTVGENKRAGILYRFDVGSLRWVRYAHVAETADRAIYPDDMAIDEDGVHLLFQWSAYPSSAVRHVGEYGIIGTDGLMRAVNNTPLPMPVAQGQLAYKPLQPGENPAISDGLKIGIQSAKFALNNGGLSHITYRFRTVDDPTGTWFGKFGVYVATWSGSSWNEELIAYVPPEQGNTSAALATTAQGGKRRVYFSVEYTSSGNTVAVIVLAENAGSGWVYSVLGNSAPTLLRLGSAPGNGGDVLYVSAPFEAKVYRYFVPEDYFPAQQFTNFDELLLTLT

>K22PH164C1 cds 10, tail spike protein

MIQQLSAAFVRIKQGITLHQWLSYNRGKRANVLDFMSAEEREDVLNFTGTRDNSEAFRQALATGCKIIDVPAGLYHVGEVTIPNKVRLFGEWAYRPYNMASDASFDKDGTMIKKVSGAPSMFLWGTACGADQIMFDGVDRTASAIHSKTDGKITVAFYRCGFYRWARVGNRNGAYLACSMHFCNINQNNVGLYNTVDGNHIGLTINANRSDGVRLETGADSNTFTNCRNEWNEGNNWNFYGCVSIQVIGEVTDRAFKYGFRISNSNVQLLNVGIRRSGRTADSTATSAQIFIENSRVRLIGVKTSAGADDAGGSITEPSPAFTFRFEGGNEGILEMTSCKLDGNTTSTISGSARPAQMRISDCAGWDDYCNVGLYRKSNGKIYNDYVITSGAAGVSALTLSFSKGPVSTNSNATIMVELLWRNTDTGGSNVAWLYIGFARASGSATAFIAKVISRDDTVGLTDVDSSPAPGQNYTVAINVTSADASTFDITFKAKSTVTTNFSLRGYVKA

>K22PH164C1 cds 11, tail spike protein

MPIIKGGYMALTRSTSPTRNFDTIGQAISFGTKFKVGDRIFIYGDEGVSFDYKVGSYLVDNGTVFQAGSGYLVRNFEGPARSSWFSSWSGLVTYMSLPGRSLVVDNEVKATSVMYLKSLSSIKFTANGVIIPNDTPAQVINILGTEPTSFTSLSGDMLADSALVTFADGAGISPGDSVVIKSDRLCDGGPNTYGVRASVLRKVLATTSTGGVTTALLDQSVHYTFLVSENAVIGKYNPVDDVELVDVKINKVADSNTLFTLGISMQYCNNVRIKGGVIQGSKRAGAGDITGRSAIKFANCRNSYVDGTHFYAIGWYGVEVLGASEDIQVRNIKAWDVRHAISLNWQTTTDGPKWGEPITFKAIDCIAHKTIQSGFDTHDIGKRIQFIRCISYDAGDDGFQARAHNVEYIGCKAYRPLLDGFAANTGVNFPIYRECIAYNAPRAGFNASYGGGHIYDCEAHFCADGIRTSGGTVQGGRYTHNSKVDIFITKDVAGTQQTPLNISEASLRYDGNGRAVYFHGTMGIDPTLVTISDCDMTGHGLAWALLSGYTVQPTPPRMSRNTLDDVNVRGIATLVAGTATVNARVRGQFSSNANTFKWVSEIKLTRLTYPSNAGPVSVTAVTQDASVPTPNPDLNSFTIKSANAADVSQVMWEIFL

>K22PH164C1 cds 50, tail depolymerase

MEVQGSLGRQIQGISQQPASVRLPGQCTDAINCSMDVVEGTKSRPGTVHIARLGDLGLIQDNTNIHHYRRGDDVEEYWMITNPLGIPDIFDKQGRKCTVTETEGAASYFNSNNPRVDYKFFTVGDTTFVVNRTKIVRARADKTPAVGGTALVFSAYGQYGTNYQIIINGVKAAEYKTASGGSASDVETIRTEVIAEQLYTNLLTWAGVSDYTVSRLGTTIVINRNDGASFTVDTEDGSKGKDLVAIQYKVTSTDLLPSKAPVGYLVQVWPTGSKPESRYWLKAEVADVNLVTWQETLGADEVLGFDGTTMPYIIERTNIVGGIAQFTIKQGYWDDRAVGDELTNPMPSFIDQSLSDIFMVQNRLCLAAGESCIMSRTSYFFQFFRQTVLSAVDTDPIDVFADASEVYALKHAKVLDGDTVLFSDNAQFILPGDKPLTKATALLRPTTTFEVDTNVAPVVTGEAVMFATKDGAYSNIREFYTDSYSDTKKAQPVTSHVNKLIRGGIYHMASSTNFNRLFALSEDNRSRVFVYDWLWQGTDKVQSAWHKWEFYGATIGGLYYSGETLYLIIKRNDGVFLEAMYMGDPLLSGSDQVRMDRTVTVSLTWDEATLSWKSSPLPWVPTQVEMLEAVLTNGDPAYLGGAFLFEYDANTRILSTKYGLGDTSQIWAAKVGQMYKVEFVPTDVIIRDSQDRVSYQDVPVIGLVHLNLDRYPDFTVEITNRKSGAVRVAKASNRIGGARNNVVGYVKPTSGTFSFPLRALSTDVEYRIISISPHTFQLRDIEWSGSYNPTRKRV

>K23PH08C2 cds 220, chaperone dependant tail spike

MADTTQFEQAVDQVIEDSERLHKVVNGSAIDTVIVEDGSTIPTLRKALLDNVYFKTPPQPWVAGTQATVFNQLYSFTNSAGTFWWYAPGASPSTPITLPADPSTSTAWKVYNDSVVVSEKFAPLNTPAFVGSPTAPTPAQGSNSGAIATTAFVNLAVAAAINSLAGSSPSYAALTVVGASTLNTLVVSGTSQFGGTLDASGVLGKFQKISLSGQTATLSFDYTAASNYLKTIISPNSVQTNNLTSAVIVNGTASADNTTMSLTGVGNNTFDYVYIRGNSSKASTEPRLKVTGTTELENVRVTGSLSGVNVGVDGLDILPNSIITTTTAEIGSDLTVNGITTLGSTTIQDLGVISSLTVDGNSTLTGGFTAGSASSVAGNLSITGLLAVTGEATVSTNLTVTGNANLNNGGTGTTTVNNLNVLGTLTGVSIDVNGKDINPNSVLATTTIEAYGSLKGASLVITGEATAPKVTANFVGTTPGSVTTPSAGTTWTPGGTGSDLTKMNNIYNVDVTNTLTIGPWANLGSAFTATIYLFQDATGGHAVTLDASYKIINGGAISTTANSVTILQVTYCGRGTVYDVAVYQRP

>K23PH08C2 cds 225, chaperone dependant tail spike

MAIPTIPLQIWAESDVVLPNAHTANKISPIADLWDKGWDLGEKPACEELNYVLNMMTWWMSYISTEQIPGMAADYLRKDQNLSDVENKATARTNLEVYSKAEGDNRYVNVEGDTMTGPLTVPRITFPSDASDTAHITTTLGTDQTYLDFVIGDNPGVAGQPNVDIMRFRFVPVNNSATVSPFNMMELNATGTNTALLRVQGNITATGTMTTGTLASTTINNGGNIQTTSLGVSGTATLQNLVVNSNNATVGGRSIVRAVNSTAANANGDLYISIGVSDIRWSGEQNKVNVDFENYGSGGRFARGPDGSVLTGLIDANASGDLYLHDIDEIRFRFLQKALDGIWYTVGL

>K23PH08C2 cds 228, chaperone dependant tail spike

MATIELPVIKIKNLSDKTLVTGSDEIIIQSSTDTEKTSINKFITDIGLLKRSEVTGITGASVIGTHSGATVQQVLDSLSNSWQNLYYFSTTGNEIGTHVDTIALSPVQRTYGVQISTPLASFSPKVDNVLSNGTTSLRWSQVYAVNSVISTSNKKKKTNLRQITPTEAKAFYEIGKLDSVWQWLSKYSSEKGAARLHSGPTVQDAIKVMLKHGLDWTKYSAFCYDSWEANGDTPAGEEFAFRKEELLFWILRATIAVQEDLDKRLSALENSLSGN

>K23PH08C2 cds 233, tail spike protein

MLQSVKISELPSATTLTEDDLIVVDQPDDTKKATLFQVFSHLEDTVEQSVLAALAQPDGASKVGLPKGGTVADAIYWITLEMFGAKGDGVYDDAIALQAAVNFAESINSGITTRAPRVGIRFAAKKYYLKQTVKVTKGTVYWEGSGMYQTVFTPHPEANSPELQYKYFFDFSSPDWVSNATRTLHEVHLRNFTFLGDTGTTLRPVFYLGGVGWDCVFDTVHIYSAALSAIVADDLFDTQFYNLRINSCGKLHDPSNAEDIFTHPIIFRGKYDNCNAIRFIAPHFENNFTGVISITGRSNNIIFTGMPKFEQNSRGAASGAKYPVISINGPLIDSIKLDNIFVSHPENITEWFLESNSRHLSIRGGSYMSPSDLSGYTAKKWFRIHRDGWASATRCVGAVIDIDMMHVDGLGDTGVAPISIENDAVVNIKAPRVANPNRFIDILYDCQVTVDDLTVLGSITAANQTALFNVTGTSVKADIAVKQQQGAVTGLIYVNANMTTESRRKSKIVLRSQNTQGGVGTLVSGSDTIGYDEGYVFQASGLVSSLGYCHTGKKMVVRCPDTANCLVTGGNIFLPGGAVTSACMITLMATNFAFRQGWTEISRVNGV

>K24PH164C1 cds 8, tail spike protein

MALTKLVAVQSPTGNQRDCANIAALRAFTCSHVGQEVTLREHTSGQGSGGGVWYCHALTAGSAVDDNGCQIINNAGQVLRRRDLKELTSSYFGLKAGDIIDPVLDNMYKASRTFNIYEAKIENPGFDKGYLLTGGKRYYCGDKPFYILSYSIGTLRGPNIWHTGDNVGITFSRFKTDGTSEQAWSGGGIRGFRIWGAASYLVQGNAGVNATPVRLSDMWQGEACDLWVTGYNNNTNGAVVSLYNEYAWTEGTIVENIMVRQSLRGLTFLRNHGTTATDSFFRVVADISFNAGVSGKSTQVMVVGDGTAAGACLVYGHDIKLTQWMSAGSWHDIVRLEDYSIIAETGVIKIVADGYGISKTTVPSTEVVHSINVRGLNARFRSRVENWSNQAGGWGLDFLNIIFQSSMYTNAMTFYESDFDALPTINPVGMKIRYNGTFTVAERQSGKVYTLNGLIPGTTLKVKLTSRNGDDLNDAVVQEWKVFVRSTNLPCIVVPMSGSANIATTDGLAVTNVSPVQTATFLKTVTPTQARNFIGQNYGLTVKNANDDNSISYAVNSGRKIRFILPANAGATTTSPYSVEIEVL

>K24PH164C1 cds 55, tail depolymerase

MAQSLEGTIQSLLQGVSQQVPRERQPGQLGAQLNMLSDPVSGIRRRPPGEIVWESTIDNPGLDSLFTEYVERGTDGRHLLINTSNGKWWLLAKNGKTIINSGNDPYFVTTVGQTSLQTASIAGLTYILNTEMAPSTTVDNTGRIDPSTTGFFYVKSAAFQKRWNVTVTSAGVDYSGDYTAPAAGSTSGNAEEVSGAYVAQQLRDSLVANGLPAGNVSVRGAYLFFYGLSNCVVSSDAGDTYAVVSNQSRVDQEQDLPAQLPAEADGAMCRVGTASSETAWYQFDYSTRTWSEVGAYGSITKITNMPRELAADDNIIARDWEGRLAGNDDNNSNPGFVENGYITGIAAFQGRLVLLSGSSVDMSASGLYQRFYRSTVTSLLDTDRISISSASAQDSVYRTAVQFNRDLVLFANSMQAVVPGSAVLTPTNASISITSTYDCDSRVTPVMAGQTVIYPNKRNDSYAGILELIPSPYTAAQYTTQDATVHLPRYIPGRVLQMQNSSVTNMAFSRMSGERNSLLVYEFMWGGSDGAKMQAAWHKWSFPYPILSVQALEDEVFLYMQGPSPGNKLLIVSMDPREGYQLGMEYRGAYSDLQKQVQVQDGVFTVPAVLRPVGWADRYREEIILTYLPSNPMGPTEVGIKEIAGENTLRVVRGVPDGTYVIGRRYRSTFTLTTPILRDQNDKLVGSGHVRLLRLDVAVRNSGHFDVQVLDTPRDVNWGGELTGILMNSKELTLGQALRMDLATITVPCRTNADTTEVTLYTKGSQELNVLDISYILRYNQRRRRV

>K25PH129C1 cds 56, tail depolymerase

MAQTPIRALEGTIQSLLQGVSQQVPRERQPGQLGAQLNMLSDPVSGIRRRPPGEIVWESTIDNPGLDSLFTEYVERGTDGRHLLINTSNGNWWLLAKNGKTILNSGNDPYFVTTVGQTSLQTASIAGLTYILNTEMAPSTTVDNTGRIDPSTTGFFYVKSAAFQKRWNVTVTSAGVDYSGDYTAPAAGRTSGNAEEVSGAYVAQQLRDSLVANGLPSGNVSVRGAYLFFYGLSNCVVSSGAGDTYAVVSNQSRVDQEQDLPAQLPAQADGAMCRVGTASSETAWYQFSYSTRTWSEVGAYGSITKITNMPRELAADDNIIARDWEGRLAGNDDNNSNPGFVENGYITGIAAFQGRLVLLSGSSVDMSASGLYQRFYRSTVTSLLDTDRISISSASAQDSVYRTAVQFNRDLVLFANSMQAVVPGSAVLTPTNASISITSTYDCDSRVTPVMAGQTVIYPNKRNDSYAGILELIPSPYTAAQYTTQDATVHLPRYIPGRVLQMQNSSVANMAFSRMSGERNSLLVYEFMWGGSDGAKMQAAWHKWSFPYPILSVQALEDEVFLYMQGPSPSNKLLIVSMDPREGYQLGSEYREAYSDLQKQVQVQGGVFTVPAVLRPVGWADNYKEELILTYLPSNPMGPTEVGIKEIAGENTLRVVRGVPDGTYVIGRRYRSTFTLTTPILRDQNDKLVGSGHVRLLRLDVAVRNSGHFDVQVLDTPRDVNWSGELTGILMNSKELTLGQALRMDLATITVPCRTNADTTEVSLFTEGSMELNVLDISYILRYNQRRRRI

>K25PH129C1 cds 60, tail spike protein

MAFSWQESVKPAGTQDIQCDIEYLDKSYIHVYLDGAETTAFTWTSSTNIRLNSPLSAETAVLLIRKTAREYLYIEFASGAPFIEGNVDTQNTQFLHLAQELVEGRSIEGFYGDINMHRYRITNLGDPVDARDAANKQYVDAGDARLDQRIDAEHAAWVAAVDNEASIRKAADDALDVRTTNLEQTYFNSNTNSFPWWTVLTEDTDTVTPGMPFTKAKVRVNGVTQTAGYSYTVNAGVVKFAKVLPAGTLVDMTIGVDTDADTSAVSSVLGLLGSNSGAGYIGTQSGVNLQQRLLDVDAAIAANSKLLSYVEKSIFEFMTQSDISIIKSTTGTVDVNYALKAAVSSGVTSLIFPPVSGVYGVGSANTADIVQLPRGMRLRGFSWRPYTVSGDSSFLTTGTTLRRNTGAPALFYGAQRMCFRDINFDGVAQSNGAFFASPNSSEQFNGTRLEGCGFYRFSVALGWGNYIAALFALRCSVSGCGDAVRNTIDSNLIGCVFNANARGVNLQSGANNNSFIACRNEWNDGDNYFAFNAIENIIVGELCDRAGRGGVVAQGEARWYVSNSVVRRSGAKQAAGSDYSANFVTVDNGTILLSGVRTAVGEDDGGGGTLSPSFCLSTLGAGRGTFIAGACDLRGFTQSAHNSKLEPNISAVGNPGFPDSVNFGKSQIAGGRYCLGSARGTLSGVAGTTSSVTISNRMPTQYAVHLLRTLLVETRLEDGRDEVLKVPLIMTNETSKSIRTWSSEIKASTERIGVSSATGVVVSFALTGTDDIAVTLTSVDGLTRKYIVTLLPS

>K26PH128C1 cds 44, tail depolymerase

MALVSQSIKNLKGGISQQPEILRYPEQGTLQVNGWSSETEGLQKRPPMVFIKSLGGRGYLGEDPYIHLINRDEYEQYYAVFTGNDVRVFDLSGYEYQVRGDRSYVAVNNPKDNLRMVTVADYTFIVNRTRQVRESQHLTNGGTFRDNVDALVNVRGGQYGRKLEVNINGVLVSHQLPPGDNAKDDPPKVDAQAIAEAIAVLLRAAHPTWTFNVGTGFIHCIAPAGTTIDILETKDGYADQLINPVTHYVQSFSKLPLNAPDGYMVKIVGDTSKTADQYYVKYDKSQKVWKETVGWNISIGLDYTTMPWTLVRAADGNFDLGYHDWKDRRAGDEDTNPQPSFVNSTITDVFFFRNRLGFISGENIVMSRTSKYFEFYPPSVANYTDDDPLDVAVSHNRVSVLKYAVSFAEELLLWSDEAQFVLSANGVLSAKTAQLDLTTQFDVSDRARPYGIGRNIYYASPRSSFTSIMRYYAVQDVSSVKNAEDMTAHVPNYIPNGVYSINGSGTENFACVLTKGAPSKVFIYKFLYMDENIRQQSWSHWDFGDGVEVMAANCINSTMYLLMRNAYSVWIAAVDFKKNSTDFPFEPYRFHVDAKRSYHISETAYDIETNQTVVNVKDIYGASFSKGTVAICESDGKITEYEPTGSSWDSTPDIHISGDISGKNIVIGFLYDFQYVFSRFLIKQEQNDGTTSTEDSGRLQLRRAWVNYQDTGAFTVSVDNGSREFNYLVNARVGSTGLRLGQKATTTGQYRFPVTGNALYQKVSLSSFNASPVSIIGCGWEGNYMRRANGI

>K26PH128C1 cds 49, tail spike protein

MDQEIKTVIQYPTGSTEFDIPFDYLSRKFVRVSLVANDNRRLLSNITEYRYVSKTRVKILVDTTGFDRVEIRRFTSASERVVDFSDGSVLRANDLNVSQLQSSHIAEEARDSALMAMPEDDAGNLDARNRKIVRLAPGEIGSDAVNKDQLDTAVGDAGGILSDVKKVQQETYDYIEKFADDTALVRGVSWVYNQGAANGGETSILIDKPTRVLAVPYIEVNGLRQEVGYHFSFDIATQRITLVKPLVAGDFLVALTTESSVPVEDLLANPTGASSIGTSDGRNVQVVLDGLTADLQTVRSAVSPKEFGGVPNTDATAAIKLAIAAALVRKAPLDLRSGPWTITETIDLTDIKTVISDATGVLRVDPTTFTSKFSNKYAVTFGNPDVTFGQGRSSHVQVIGTLVVSGLNRAGALNGVYFKGSWLASNVVRVSGLNGSAIVLEAVWDSVFQSLSAELCGNESNYQIDVRGGGDTSNCLHIGRIQSERAYHKCLRISAIRSVFNTIHAERTAVLTTDDGSTNADGTRYTTLMFSLGNSVVNQLIHDAISGNAPDGRPTVGMASSRIDADYCVINAAGLSGSGLSSNSGRNTTWNGMSVRKWTFSGTATGHTIVSPRIIESLAPNNSITVKGGTAGQVSFGYNAKDVQIDSMAIDDLSFPNTIRGNINFTSCTFPETLTIGSTRAPEGYTAQSTLGETNTPVTFTDCVHLGTLAGAFQSRCVWKGGYIANISLVSRAVVELYNVSTKSFSATGDRAYITRQVRATNVGSWGVPTHVAYPIGTITERLGTDELNVGTGFRNADGTTTGFVKIY

>K26PH128C1 cds 50, tail spike protein

MLDKLNQPKGSTIGVLRDGRTIQEAFDELGGSLTLADIRSRTFTKVGEEVTIGEHTAGQGMGGGTFRVVSLTPAGYTDDNGCQIITNSGAVLRRKTHFISSDMFGLVGGGDIIACLNNMYKASRTLKIEEVLVCRQTNGQTYRADTRTAPGFSAEITDGFGFYIRGLGIGYRGPRIDHVGGGVLLRISKNRNTAVDFWATGGISGLVIQGRADTWTGDNVNTDATPIRIQDIIGCELKDIFIQGYRANTSGAAFSLYNVTGWTELCKFDNIMVRNSSVVIRCHRDPNGNGGTDSFFGLKGNIEANAGANGPTTFLRLGDGTSEGRCFLYGHDLKITGWMSSSAGHTGVHVTDYSTCTEGLFTFVFDGYGISQGAASAVLHLIRVDGLNGRFDCDVRNYSGQSGRAPISLLQQIWDSCVRSSPDAINNSLNRAYPCIRPRGMKMKFEGTFEMGSTQNGVTIPLGNLLPGMRLMVTLHSWNTDKWQLKVTKYDVQVMSIDYPCVITPVFGVGPTVTPTATTVSTPDGTTQALTAVAVSHNDRIQNGSLGSMSLRLNNGRPDNSTSYAVGSGLKLYVELPGDPSATVAKNYSVEIEIQ

>K27PH129C1 cds 43, tail depolymerase

MALVSQSIKNLKGGISQQPEILRYPEQGTLQVNGWSSETEGLQKRPPMVFIKSLGGRGYLGEDPYIHLINRDEYEQYYAVFTGNDVRVLDLSGYEYQVRGDRSYVTVNNPKDNLRMVTVADYTFIVNRTRQVRESQNLTNGGTFRDNVDALINVRGGQYGRKLEVNINGVWVSHQLPPGDNAKDDPPKVDAQAIAEAIAVLLRTAHPTWTFNVGTGFIHCIAPAGTTIDILETKDGYADQLINPVTHYVQSFSKLPLNAPDGYMVKIVGDTSKTADQYYVKYDKSQKVWKETVGWNISIGLDYTTMPWTLVRAADGNFDLGYHEWKDRRAGDDDTNPQPSFVNSTITDVFFFRNRLGFISGENIVMSRTSKYFEFYPPSVANYTDDDPLDVAVSHNRVSVLKYAVSFAEELLLWSDEAQFVLSANGVLSTKTAQLDLTTQFDVSDRARPYGIGRNIYYASPRSSFTSIMRYYAVQDVSSVKNAEDMTAHVPNYVPNGVYSINGSGTENFACVLTKGAPSKVFIYKFLYMDENIRQQSWSNWDFGDGVEVMAANCINSTMYLLMRNAYNVWIAEVDFKKDSTDFPFEPYRFHVDAKRSYHISETAYDIETNQTVVNIKDIYGASFYNGTVAICESDGKITEYEPMGNSWDATPDIRISGDLSGKDIVIGFLYDFRYVFSRFLIKQEQNDGTTSTEDSGRLQLRRAWVNYQDTGAFTVSVENGNREFNYLVNARVGSTGLRLGQKATTTGQYRFPVTGNALYQKVSLSSFNASPVSIIGCGWEGNYTNRASGI

>K27PH129C1 cds 48, tail spike protein

MMNQDIKTVIQYPVGATEFDIPFDYLSRKFVRVSLVADDNRRLLSNITEYRYVSKTRVKLLVETTGFDRVEIRRFTSASERVVDFSDGSVLRAADLNVSQIQSAHIAEEARDGQYYSLNLNDLGQFDAKGTKIINLGVPTSPQDATTKLYVDTNDLALSQRILDEIKARAEGDIEMLRQAKMYSDSQIPGILPDGATSVGYWLPSATALMNDDSGHPVVRVRGFYTADDGGEGSWVWTNEVDAQKAGTHDILTASVYDKVGRRYRINITIGAVNAAQNGARRLTYAQATDRTTDDFVCLGQVISGITSLMPLPVSTNNSESGYIGDYRINLTISPGRYRIGKESGGIDSGMVINAWGARVHVVAGKSYTRAITGKWIHGLVHGYDQIKRKWEAVGKQAFWGSVSLKDVVINGGFWIGDHDLRTPASECSAGVGTLLLNPEGVIMNGVHQRNFNWVHVAMGAMIEETWYRTSKGRLFDDNELDYPYVMDFMTSNVPPVTRRFGNFNRVAYHGCKFESGRRGVFRNGVDWSGCYNTEIINNIAWRNPANADGSLPEFIAVLTGTAFHMSAGYMGPAAAKDLNAGVASVYSSTQNIKFDATYTEWTYAFMMISSWGFVDSASRLQGLSLDLVSVYKDNFADYGQIIFEEGCFPTMSEDGSATYPAGFKHFDTPQGQSPYVIGKPVRDVGAFRNQGFDFKYATNNVYLTAGTDWESWRDRPYAREMFNAFGLQINSGTAFLPLQNPADKSMTCIWYKDFTGNFDTRKVIQFITAAAQEGPNADEALYKSYAERVVDFGNGYKMMMIPNKRLTAWDGIYTYARNANITVEVSADTPIALIAVEAYTGGVPLFPNGVPNYVPESSCASVVPTAPQVGFDSNLGGGIFFDGDVVGPWVHVRRNKVGRRITPQVTAGSVLSTRRVSGQGITLESALKTTFTATIVSSNADTTTISIQSASLPSVAVGIPLYVQSGSSTGFTGLAHLHLRVMLANGKADNKYVVNGKLGEAGDNLTIDQSQIAAYTLLADFTQTAITAETMTLSGVARASAFRASVGVDVGYGGSSGPKVVAFYFNGGTTQTHAITATSLNGMSVNANGNLSINGHTFSTQDGTHTLGTAQNRWAAVWSANGTLQTSDERFKILDGDIPEELLDAWERYVKVKVYRMKSAVELKGPDARKHVGYIAQHVIEALTQAGLDWTKYGVVGFDEWEANSFGPAGNRYSLRYDQCAAIENAVLDRRIRRLEELLLNK

>K28PH129 cds 24, chaperone dependant tail spike

MALYREGKAAMAADGTVTGTGTKWQSSLSLIRPGATIMFLSSPIQMAVVNKVVSDTEIKAITTKGAVVASSDYAILLSDSLTVDGLAQDVAETLRYYQSQETVIADAVEFFKTFDFDSLQNLANQVKADSQSAGASATAAAASESKAKTSETNAKASETAAKTSENNAKSSETKAKTSETNAKASETAAKTSETNAKASETAAKTSETNAKSSENKAKTSETNAKASETAAKTSETNAKASETAAKTSETNAKASETAANSAKTDAQTAKGQTQNLRDQVVDLVAGVQAPDKLSRVASSSESWMKIANIKSTGSAYAFVQFIIGGGSDYGAANVPVDIFSLSGRGLPASPLTSGNIDAWFTQRSLIAARPNAKRVNLGAVKKADASYDIYLHAPGGWIPSLWLNLLNVQANNGSITGPIIDRTGYSWITTEPTGIVYNNPADYLLANDSTIPRTNVANTFSQPQAISVPGGNATLTLNGCTVRANNNNVLVLSTPSGSEGMSFRPNGDTSTNGQMTISGNGDVLVNGTVKSNGVDVTASQKLPLKETTATTGVGVNFIGNHTTECSFGIENTAGGSAVFHNYTRGASNSVTKNNQLLGGYGSRPWLGSDYTEHSNAALHFLGAGDASGTNHGGWIRLLVTPKGKTISDRVPAFRLSDNGDLWIVPDGAMHSDLGLVRSFETLNAAVPKFNGPTNQDGRGLKIVSSDGAPEINMIAPRGTNTSSPAVRAMWCDGSLGNSAKYVGAVQAWSTFFFGASGHDGEKFDSMRGAVNIQATEGWGKTSTPTRILFETCAAGSTARTARWCVDHNGNFVPMGDGGYDIGWGSGRVNNIYAKNGAINTSDGRMKNDVRAMSDPETEAAKAIAKEIGFWTWKEQADMNDVREHCGLTVQRAMEIMESFGLEPFKYGFICYDKWDEQTVVSEYGPANEDGSENPIYKTIPAGDRYSFRIDELNMFIAKGFEARLSALEDKLGM

>K29PH164C1 cds 25, chaperone dependant tail spike

VNINQIGVCIMATYKVGKVKINGNGLMTGTGTNWTAANALVRVGATVVLATNPVRIYTVGSIISATSIQLSDWGSDAAITTDTNYSILLHDGLTVQGLAQDTAETLRYYRNFENTLGDAAKATIGEAPGNVMKVGAFGLGANKPGGTIASTTYPSANALFKALMDAGCGWWRSPGSSENGIFGHGSSYFSYVSDTCSAINVAYETGRVIVLASNRAKINNGYQPARNVLYGSANPPDLNNETRGVLNMANGGTGSTNASDARLAFGLRMIDVPANTSGAVRCIKIASIRTPGAAGSFASMSIYGGSGIGSGPRVNIDTVIISGRNSGNENNPQGDVSILHRCLRAGNDTLKFGLVKTGEGQYDVYMKVMGYVQGLRIVVDTILSQSFIDGPVYTGSFGSIGYVNESEIPTPAGNIGWASTYDIVNQYNKDIAQEFSEVTINRASTNDQEARLLFNFGKTESRGAELRSTYTNGNIVLATGHNGASSPVFGEIYLRTAGSTSSTGQFKFDKEGSATATGGQWKNSSDIRLKRDFKPISSPLESVMSFRGATYEMKASGVRAIGVIAQDIEKLCPDAIGRMEIELDGEVIPDAMSVDTAGFAAAYSVQALKEVVKLMDLMLEDPEAASVRIKALKEMINDELPE

>K2alfa62 cds 23, tail spike protein

MALYREGKAAMAADGTVTGTGTKWQSSLSLIRPGATIMFLSSPIQMAVVNKVVSDTEIKAITTNGAVVASTDYAILLSDSLTVDGLAQDVAETLRYYQSQETVIADAVEFFKEFDFESLQNLANQIKADSEASESSAAAAAASESKAKTSEDNAKSSENAAKNSEVAAETTRDQIQQIIDNAGDQSTLVVLAQPDGFDSIGRVSSFAALRNLKPKKSGQHVLLTSYYDGWAAENKMPTGGGEFISSIGTATDDGGYIAAGPGYYWTRVVNNNSFTAEDFGCKTTATPPPNFNVLPAELFDNTAMMQAAFNLAISKSFKLNLSTGTYYFESSDTLRITGPIHIEGRPGTVFYHNPSNKANPKTDAFMNISGCSMGRISSINCFSNSYLGKGINFDRSVGDNRKLVLEHVYVDTFRWGFYVGEPECINQIEFHSCRAQSNYFQGIFIESFKEGQQYGHSAPVHFFNTICNGNGPTSFALGATYKTTKNEYIKVMDSVNDVGCQAYFQGLSNVQYIGGQLSGHGSPRNTSLATITQCNSFIFYGTDLEDINGFTTDGTAITADNIDTIESNYLKDISGAAIVVSSCLGFKIDSPHIFKINTLSTIKLMNNTYNYEIGGFTPDEALKYNVWDANGLATNRISGVIHPRLVNSQLGINSVAFDNMSNKLDVSSLIHNETSQIIGLTPSTGSNVPHTRIMWSNGAMYSSTDLNNGFRLNYLSNHNEPLTPMHLYNEFSVSEFGGSVTESNALDEIKYIFIQTTYANSGDGRFIIQALDASGSVLSSNWYSPQSFNSTFPISGFVRFDVPTGAKKIRYGFVNSANYTGSLRSHFMSGFAYNKRFFLKIYAVYNDLGRYGQFEPPYSVAIDRFRVGDNTTQMPSIPASSATDVAGVNEVINSLLASLKANGFMSS

>K2PH164C1 cds 23, tail spike protein

MALYREGKAAMAADGTVTGTGTKWQSSLSLIRPGATIMFLSSPIQMAVVNKVVSDTEIKAITTNGAVVASTDYAILLSDSLTVDGLAQDVAETLRHYQSQETVIADAVEFFKSFDFDSLQNLANQIKADSESAESSAAAAAASESKAKTSEDNAKSSENAAKNSEVAAETTRDQIQQIIDNAGDQSTLVVLAQPDGFDSIGRVSSFAALRNLKPKKSGQHVLLTSYYDGWAAENKMPTGGGEFISSIGTATDDGGYIAAGPGYYWTRVVNNNSFTAEDFGCKTTATPPPNFNVLPAELFDNTAMMQAAFNLAISKSFKLNLSTGTYYFESSDTLRITGPIHIEGRPGTVFYHNPSNKANPKTDAFMNISGCSAGRISSINCFSNSYLGKGINFDRSAGDNRKLVLEHVYVDTFRWGFYVGEPECINQIEFHSCRAQSNYFQGIFIESFKEGQEYGHSAPVHFFNTICNGNGPTSFALGATYKTTKNEYIKVMDSVNDVGCQAYFQGLSNVQYIGGQLSGHGSPRNTSLATITQCNSFIIYGTDLEDINGFTTDGTAITADNIDAIESNYLKDISGAAIVVSSCPGFKIDSPHTFKIKTLSTIKLMNNTYNYEIGGFTPDEALKYNVWDANGLATNRISGVIHPRLVNSRLGINSVAFDNMSNKLDVSSLIHNETSQIVGLTPSTGSNVPHTRKMWSNGAMYSSTDLNSGFRLNYLSNHNEPLTPMHLYNEFSVSEFGGSVTESNALDEIKYIFIQTTYANSGDGRFIIQALDASGSVLSSNWYSPQSFNSTFPISGFVRFDVPTGAKKIRYGFVNSANYNGSLRSHFMFGFAYNKRFFLKIYAVYNDLGRYGQFEPPYSVAIDRFRVGDNTTQMPSIPASSATDVAGVNEVINSLLASLKANGFMSS

>K2PH164C2 cds 24, tail spike protein

MALYRQGKAAMDANGIVTGTGTNWQSALTLIRPGATILFLSSPIQMAVVNKVVSDTQINAISTNGAAVPSSDYAILLSDSLTVDGLAQDVAETLRYYQSQETVIAEAVDFFKDFDLSTLQELVEQAKEEAAASQQSASASQQSASASQQSASASQQAAAASQQAAAASQQAADDAEATRDEIQQIIDDSGEQSTLVVLAQPNGAKNIGRCSDIATLRTIEPTLPNQKIEVIKYASGYKPITGYFEYDQTDSTSVDDGGVCIVTAGGKRWKRIFDGAVNVAWFGIPSDGDITNAINKAISYAKPKRLGLVISAGQYKYTGSEMLEIDLGFISLVCHVGCASIDFSGVTSTYAINVYSSATYPAPLYRNTVNKLSGIECFGAKVAGKNGLLIGRPGGAQYYNGQCCIEHCSFHDFDYVVSCANSTWRYKFNECVITKGITSIFYAPAGLLDSGESITFNGGMVADASGAPIIIACDAFNLGLDGTSTLNTRVQITGNGATVSMSGMGNIENPGQSVWYPYVTCTGTGARFILSESTLTINQPLAQTQPIIYVGINAFIIFNVVKFPGNPYKFEQNSSDMVRAFVEGPGSVMCNACTSDIASGAGNIPVHRSLSPVYNSGFEQGNNTGWSINNAGSASQTAVVSAEYAKAGSYGMRMTSIAGLSIFATQQFNVKPGQYYMTSFWARVVNIGANGNAAGNINLSFYSQNGTQIAGPTANLPVNVADWAVYGSFIRGIVPPGAATAVISMRAYDGAVVDFDGVLINFI

>K30lambda2_2 cds 20, chaperone dependant tail spike

MAITTRIIAQQVTALDGANSRVSKYPKFTVQLGYSVSSLAATELLDAATKSAASAAAAKTSETNAKASETASKNSQTAAKTSETNAAASAQLAQNLAGKTSLVTPLGVMTGSAEAKIASITIAANQSSSVHVLFALYATGNGANRDDIYNMEIVSLALPGPVTSVTADNIGSFLSHRVIGPANTNGFMVGLKSTIEGSNVTYDVYLKSRSSFRDPKMAFLSGSISVTPPTGPLVDGTAPAWKTTGFDTEVIYVNRAQVIDDGISLAKLKALRVTGDSGSEYLTLTARPTGGIISLNNRSIYLRPAGTEDTVASVEIAPNGNLVFPLTATKNHILSWQGGARIRANDTGTMVISGNNGSGQTTGFLAFRPNGDSATTTEIQMRSDGNLKQTAANVDEPNVLTRRDTVINLISSRAPEAGVTSEALADLDVNNTESGNDQWGRGVQLQQAGAGTKNTPGNTTGRLGTIATFRQSQYRLSQIFFDSNTGNGGTSLGVFVRSLRADSGTSPRPWFALYHEGNKPNIQSGIAGITIDGNGFVKKASPIAKLIAEIPSKEDSFFWTGVETVGGYVGCNAEAQGVFAVKTGLGRYTIKGSLGWNTEGWKFELPRDDNGNMLCFVESDWNEEEKELNIQVFTRKFDINTGNIIAGEPMEIPQGRWIDLRLEMPKVEIPEVEFPEDPEVE

>K30lambda2_2 cds 152, Putative tail depolymerase

MGFYAGRIGDKKVLSLTSGNNKDVNNHTNPGWDTIFHSDMPHVVVLETHERDLWDGGDWYRCTRMPDRIIQVLSADYDRVVLTEVEFEDGTRRFIYGTSLGVGAKAYNAYFSNTVGSQVSAGTMASMKTNVCASADLHMDVSFYFEETPGTINEKLRDGTGCMYTWGVNSEWGDRGPGPPVGAPIPPNFETIIKAGWVLYRGAFSGNIAGSVSPPNRPLTIGVDAMRHPWMRTTGVNSICLRGETLNRNMYGHMGPRYGQSSNPVGGPYSHNIQTESYQEIQYKAGFFRGPPNNFMGWENTDNNNAGSGWGNNAIYRDNNFRVPKRVRWYITNMKYNGQGFYAEDVFGSRNQEIKISPREFIVNGINLMNTGWKFINQNDINYSPGNRPDIRVIATNVARFSGNPTVGNNGYVHFNQPLTRPDNGAEFGQGNISEMHVTTVGVYNFRSDAQWYVKSNPPEIGNQWGPVWSESTRPLRLVGGTGSADIGGNLRTSGNASHHLATLWLGVNNSRNGACVVTLDWKNDEWIAAAGIGCYNPLEDLTQWSEVDSRLRIFGNHFQKRVHQIMCLPVNMCVPFHFIRGTVTQCGVIPGNNAMQMKAMWAPATTNSATQGDYAIIYWLIARADGSVEVWVNVEMINIMNMRVILPEVRIAVQRLA

>K31PH164 cds 25, chaperone dependant tail spike

MALYRQGKAAMDANGVITGTGTKWQSALSLIRPGSTIMFLSSPIQMAVINKVVSDTQINAITTNGAVVPSSDYAILLSDSLTVDGLAQDVAETLRYYQSQETVIADAVEFFKDFDLEALQNLANQVRADSEAADASAAAAAASEGAAKTSETNSKASEQAALTARQQAGSARDQTQDLYNQTVDLVAGVQAPDKLPTSPGSSRAWMKIANVKNAGQSNCFAQFIIGGGANFGSANLPIDIFSISGRGLPDTLTSNNIDANFTQCALIPASNSTSRLKLGVVKNTDGSFDVYLLSPNGYFPVMWLNRLNVQSNNGLITGPIIDRTGYSWITTEPAGIVYNSPSDYLMANDSTIPRTNVANTFSQPQAISVPGGNATLTLNGAVVRANNNNAIVYSIPEDGQGMYFRPNGDMNSAKQVVFDAANFTVTGLNATFSNAVTMLSTLRVNGASNLRGGVDVTASQKLPLKETTATTGVGVNFIGDNATECSFGIENTAGGSAVFHNYARGASNSVTKNNQLLGGYGSRPWLGSDYTEHSNAALHFLGAGDTSGTNHGGWIRLLVTPKGKTISDRVPAFRLSDNGDLWLAPDGAMHPDLGLVRSFETLNAVVPAFNAPTNQDGRGLKLVGSPAPEINMIAPRGSNTSSPAIRAMWCDGSLSDETRYIGAVQNGSTFYLGASGHDGEKFDSMRGSVTIKANAGWGKTSTPTQVVFETCESGTITRMPRWGVDHNGTLMPMADNKYNLGWGSGRAMNIYAVNGTINTSDARLKNDVRTMSDPETEAAKEIAKEIGFWTWKEQADMNDVREHCGLTVQRAMEIMESHGLKPFDYAFICYDKWEETTVVDSYGPAKEDGTENPIYKTIPAGDRYSFRLDELNMFIAKGFEARLSAIEEKLGM

>K32PH164C1 cds 20, chaperone dependant tail spike

MAITTRIIAQQVTALDGANSRVSKYPKFTVQLGYSVSSLAATELLDAATRSAASAAAAKTSETNAKASETASKNSQTAAKTSETNAAASAQLAQNLAGKASLVTPLGVMTGSAEAKIASITIAANQSSSVHVLFALYATGNGANRDDIYNMEIVSLALPGPVTSVTADNIGSFLSHRVIGPANTNGFMVGLKSTIEGSNVTYDVYLKSRSSFRDPKMAFLSGSISVTPPTGPLVDGTAPAWRTTGFDTDVIYVNRAQVIDDGISLAKLKALRVTGDSGNEYLTLTARPTGGIISLNNRSIYLRPAGTEDTVASVEIAPNGNLVFPLTATKNHILSWQGGARIRANDTGTMVISGNNGNGQTTGFLAFRPNGDSATTTEIQLRADGNLKQTAANVDEPNILTRRDTVINLISSRAPEAGVTSEALADLDVNNTESGNDQWGRGVQLQHAGAGTKNTPGNTTGRLGTIATFRQSQYRLSQIFFDSNTGNGGTSLGVFVRSLRADSGTSPRPWFALYHEGNKPNIQSGIAGITIDGNGFVKKASPIAKLIAEIPSKEDSFFWTGVETVGGYVGCNAEAQGVFAVKTGLGRYTIKGSLGWNTEGWKFELPRDDNGNMLCFVESDWNEEEKELNIQVFTRKFDINTGNIIAGEPMEIPQGRWIDLRLEMPKVEIPEVELPEDPEV

>K32PH164C1 cds 151, putative tail depolymerase

MGFFAGRIGDKKVLSLTSGNNKDINNHLNNGWDTIFHSDMPHVVILETHEVTLWDGGDWYRCSRMPDRIIQVLSADYDRVVLTEVEFEDGTRRFIYGTSLGVGAKAYNAYFSNTVGSQVSAGTMASMKTNVCASADLHMDISFYFEETPGTINEKLRDGTGCMYTWGVNSEWGDRGPGPPVGAPIPPNFETIIKAGWVLYRGAFSGNIAGSVSPPNRPLTIGVDAMRHPWMRTTGVNSICLRGETLNRNMYGHMGPRYGQSSNPVGGPYSHNIQTESYQEIQYKAGFFRGPPNNFMGWENTDNNNAGSGWGNNAIYRDNNFRVPKRVRWYITNMKYNGQGFYAENVFGSRNQEIKISPREFIVNGINLMNTGWKFINQNDINYSPGNRPDIRVIATNVARFSGNPTVGNNGYVHFNQPLTRPDNGAEFGQGNISEMHVTTVGVYNFRSDAQWYVKSNPPEIGNQWGPVWSESTRPLRLVGGTGSADIGGNLRTSGNASHHLATLWLGVNNSRNGACVVTLDWKNDEWIAAAGTGCYNPLEDLTQWSEVDSRLRIFGNHFQKRVHQIMCLPVNMCVPFHFIRGTVTQCGVIPGNNAMQMKAMWAPATTNSATQGDYAIIYWLIARADGSVEVWVNVEMINIMNMRVILPEVR

>K33PH14C2 cds 25, tail spike protein

MALYRQGKAAMDANGVITGTGTKWQSALSLIRPGSTIMFLSSPIQMAVINKVVSDTQINAITTNGAVVPSSDYAILLSDSLTVDGLAQDVAETLRYYQSQETVIADAVEFFKDFDLEALQNLANQVRADSEAADASAAAAAASEGAAKTSETNSKASEQAALTARQQAGSARDQTQDLYNQTVDLVAGVQAPDKLPTSPGSSRAWMKIANVKNAGQSNCFAQFIIGGGANFGSANLPIDIFSISGRGLPDTLTSNNIDANFTQCALIPASNSTSRLKLGVVKNTDGSFDVYLLSPNGYFPVMWLNRLNVQSNNGLITGPIIDRTGYSWITTEPAGIVYNSPSDYLMANDSTIPRTNVANTFSQPQAISVPGGNATLTLNGAVVRANNNNAIVYSIPEDGQGMYFRPNGDMNSAKQVVFDAANFTVTGLNATFSNAVTMLSTLRVNGASNLRGGVDVTASQKLPLKETTATTGVGVNFIGDNATECSFGIENTAGGSAVFHNYARGASNSVTKNNQLLGGYGSRPWLGSDYTEHSNAALHFLGAGDTSGTNHGGWIRLLVTPKGKTISDRVPAFRLSDNGDLWLAPDGAMHPDLGLVRSFETLNAVVPAFNAPTNQDGRGLKLVGSPAPEINMIAPRGSNTSSPAIRAMWCDGSLSDETRYIGAVQNGSTFYLGASGHDGEKFDSMRGSVTIKANAGWGKTSTPTQVVFETCESGTITRMPRWGVDHNGTLMPMADNKYNLGWGSGRAMNIYAVNGTINTSDARLKNDVRTMSDPETEAAKEIAKEIGFWTWKEQADMNDVREHCGLTVQRAMEIMESHGLKPFDYAFICYDKWEETTVVDSYGPAKEDGTENPIYKTIPAGDRYSFRLDELNMFIAKGFEARLSAIEEKLGM

>K34PH164 cds 24, chaperone dependant tail spike

MANISDQLAADIHNAFSKYYTDLANQDQIFFGVGDVQITKQDGTTATIRSWNKVIGSVDTAAQRGQANTFTALQTFSAGINVSVGNINVMNDNSMVILGKNSDLALLKKSGQGGTIAVGSGTPFKIQRTNTATVSPASTVEDILTIGTDKKTTLAGALNTGGDVVANGFLYAQSIELSFGTPYIDFHFNYSTDDFTGRIIATAADQISVQRSHLRVDRDLRVFGMADIKGWAQCGVDLSANRTDFGSPAIGSLVSGGRIRSRMLGRGGNVDPSGAWGGFYVEEYVGTEHRIIMYMDGFGRTDAWSFRSGGTISTPKGDVLTTGSDVRLKTDFTQAPEKACERIERLGVCQYRMKGESRVRRGFIAQQADTVDKVYTYQDVEQEIDGERIKVMNVDYVAIIADLVASVQELRQELKELKGE

>K35PH164C3 cds 43, tail depolymerase

MVTHTFRVSSVVNQKLKEDQMALVSQSIKNLKGGISQQPEILRYPEQGTLQVNGWSSETEGLQKRPPMVFIKSLGDRGYLGEDPYIHLINRDEYEQYYAVFTGNDVRVFDLSGYEYQVRGDRSYISVVNPKDNLRMITVADYTFIVNRTRQVRENQNVTNGGTFRDNVDGIVNVRGGQYGRKLEVNINGVWVSHQLPPGDNAKDDPPKVDAQAIAAALADLLRVAHPTWTFNVGTGYIHCIAPAGVTLDEFQTRDGYADQLISPVTHYVQSFSKLPLNAPDGYMVKIVGDTSKTADQYYVKYDASQKVWKETVGWNISVGLEYHTMPWTLVRAADGNFDLGYHEWKDRRAGDDDTNPQPSFVNSTITDVFFFRNRLGFISGENIVLSRTSKYFEFYPPSVANYTDDDPLDVAVSHNRVSVLKYAVSFAEELLLWSDEAQFVLSANGVLSAKTAQLDLTTQFDVSDRARPYGIGRNIYYASPRSSFTSIMRYYAVQDVSSVKNAEDMTAHVPNYIPNGVYSINGSGTENFACVLTKGAPSKVFIYKFLYMNEDIRQQSWSHWDFGDGVEVMAANCINSTMYMLMRNGYNVWIAAVDFKKESTDFPFEPYRFHVDAKRSYHISETAYDIETNQTVVNVKDIYGASFSKGTVAICESDGKITEYEPTGSSWDSTPDIRISGDISGKDIVIGFLYDFQYVFSRFLIKQEQNDGTTSTTDSGRLQLRRAWVNYQDTGAFTVSVDNGNREFNYLVNARVGSTGLRLGQKATTTGQYRFPVTGNALHQKVSLSSLNASPVSIIGCGWEGNYTNRASGI

>K35PH164C3 cds 48, tail spike protein

MDNEIRTVVTYNLDGAIEFDIPFDYLSRKFVKVSALTEGGDKKLLENLLDYRYVSKTRIRLIGTQPGYSKVEIRRITSASERVVDFNDGSVLRATDLNVSQLQSSHIAEEARDQTVEEARLYLVDILEAADSARDSAASAKASEESSRESYENTVIIEKSLSDDIWQQLIEQAERFQQFLRASGYIFKGEYTSGIIRLESLSEVFQYNGQIWRAKAATTLPFTTTGTTAASWANDVNGLVSVGDAYLRQELFTPSGTSLVGHTRSKLSGSVQTLSKMLDNLPVSIWEFADRIVHKPTSDYQTWDWHPAVQATVDYCMSYSTAIGINPGMMLERKAYAPGGLYLLGDTVRITRVGSSAGSLMSAFTIEGDGRTSTIFQPTTEGGTAFSVKAASLNIRRIGLRAGAKGQTGVAYGDKNVWLPAAHCVAEELGTSGFSHGVRVFHAFDSSWFDIFIQSVDDTLDGTLAGGFTIETYSGPASLNGTTGGDNSNQLLLVRPTIETAVGVNCAMFIINGRSSDYLHHAITVIGGHIETHNSTTKLYNIKNGYNIQFYGTVMTQNGGVPVDKDTGLPLAEDKFYRLGWIENTKMLSFNSTRQVTTNRIGTPSESDTKAIAIVGSCQNVTFPGSHLTGPYQDQNATRHNAGLVIDYSAATLGKRAFNLDGCTVGDPASRKINQIIRVGSLSGTHDFAMEVDETEGGLTVKYSTNFTDSVEGSTLLGLSRTGDLSVAGGISLGSGSGAATRSISANGSRVSFDAAGRIYLTPSSGNQLVVSPTAVVPDVDGVLVLGSPASRFSSLYVVKVMYNNSVGDFAGSGSPEGVVVAAAGSTYRRIDSGASGARFWTKETGVGSTGWKGWA

>K37PH164C1 cds 41, tail depolymerase

MALVSQSIKNLKGGISQQPEILRYPEQGSLQVNGWSSETEGLQKRPPMVFIKSLGPRGYLGEDPYIHLINRDEYEQYYAVFTGNDVRVFDLSGYEYQVRGDRSYVTVNNPKDNLRMVTVADYTFIVNRTRQVRENQNRTNGGTFRDNVDAIINVRGGQYGRKLEVNINGVWVSHQLPPGDNAKEDPPKVDAQAIAEAIATLLRTAHPTWTFNVGTGYIHCIAPADTTIDILETKDGYADQLINPVTHYVQSFSKLPLNAPDGYMVKIVGDTSKTADQYYVKYDKSQKVWKETVGWNISVGLEYHTMPWTLVRAADGNFDLGYHEWKDRRAGDDDTNPQPSFVNSTITDVFFFRNRLGFISGENIVMSRTSKYFEFYPPSVANYTDDDPLDVAVSHNRVSVLKYAVSFAEELLLWSDEAQFVLSANGVLSTKTAQLDLTTQFDVSDRARPYGIGRNIYYASPRSSFTSIMRYYAVQDVSSVKNAEDMTAHVPNYIPNGVYSINGSGTENFACVLTKGAPSKVFIYKFLYMDENIRQQSWSHWDFGDGVEVMAANCINSTMYMLMRNAYNVWIAAVDFKKESTDFPFEPYRFHVDAKRSYHISETAYDVETNQTVVNVKDIYSASFSKGTVAICESDGKITEYEPMGDSWDSTPDIRISGDVSGKNIVIGFLYDFQYVFSRFLIKQEQNDGTTSTEDSGRLQLRRAWVNYQNTGAFTVSVDNGSREFNYLVNARVGSTGLRLGQKATTTGQYRFPVTGNALYQKVSLSSFNASPVSIIGCGWEGNYSRRANGI

>K37PH164C1 cds 47, tail spike protein

MRPLVQTVRLFAKWLDHVKDVVIPAKVKLFGTYSYKPYNVTSDASFGTDGTIIRKVAGADNMFLWNTACAAEGVMFDGRDRTSPAIQSQSGGKITVGFFKCGFYRFDRVGNRRGAYLGCSFQFCNFNQNNIGIYNTVDGNHIGCTINANKSHGVMLETGANSNTFTNCRNEWNEGDNWNFYGATSIQVINELCDRAFGYGFRISNSSVTLINVNIRRSARTAASGAASAQIYFESSTLKMIGVNSSVGGDDTGGSITEPSPDYFFRMAGTSEGRLEISDSRLTGYTVGLISGTARPSVIRVINSPGWEDTINEGVARISGGRPYIGTMPTATGPANVSPAVLGLSCGGVNTYDNDMFDIHLTIRNTNNGGHNGAILTVLLYREGGAARATIVRVDSRSNAVGEGDVNSTSADPQQVYQVSVEVTANDASTFNLLVSTKSDNNASYRFRAKVKP

>K37PH164C1 cds 48, tail spike protein

MLDRLNQPKGSTIGVLRDGRTIQEAIDDLYVFKDSQGFINVDTQAGTTLEEKLRNAFTIANTLLVGVRLTAGKVYPLTGTTPLEVNLAKFSLFTSGGRATIDASEFTGPTALWIHATGSYPTPMYRNTTNYMESIELVGGLKAGVDGWTWGNRGMTTGTEYNGQCIIRGCSVYKFDNCIKCTDSSWRYKVSDCMISTGITSVFNAPAGLIDSGESITFSDTQFSDSNGAKFIIACANFSVGMSGTSVLNTPVVISGNGASLLIDGMGNNENPGRSAWMRYVEVTGIGARFILQSSTLVCNGPSSQTRPLVLVGAKARAIFIAVKFPGNPYMFHMNNPEKVRTFCEGEGIVKTIACTYDIESGAGNIPVHRSLNRFYNNGFEQDLAGWALNVGGDPAQTATIVTDDTNSGGKAVKVASLDGKSVFLTQNVRVSSGEEFASFVAYKVNKAASGSTPGNLTVTFKSENGTTIGTGSTSNFSNTVGAWQQGGLFCRGVAPVGAVSAEISLRVRDGAEVILDDVIVNFL

>K38PH09C2 cds 24, tail spike protein

MALYKEGKAAMAADGTVTGTGTKWQSSLSLIRPGATIMFLSSPIQMAVVNKVVSDTEIKAITTNGAVVASTDYAILLSDSLTVDGLAQDVAETLRYYQSQETVIAEAVEFFKDFNFEALQNIANQIKSDSEAAESSAAESAASENAAKTSETNSKASEVAAENARDQVQQIINDAGEQSTLVVLAQPGGASNIGVDDGLTLMDITISSVTAFMAMNISPSKIIVGGFYTRGDGGDGTWVATGNTDASKAGTHVVTEAKIYNASGVEYQLQVGVGMGIISPKSNGAKEAANYAETQLDTDNFVCLGQAINGIISALPLWMQPGNNINNQSTYSVINFNISSGLWRIGKEAAKLRSNVIYNLGAATIYVKASASRKFQVTGKYLNGFEHSVEDVEEVYTALGSKLYWESVSLGFTKMTGGKIIGDHVPGTLDSDCTAGVGILALNPWYCSFEDIRIENFRVNLVGMQVRIDEDGVLPSSIVPYKSSLLPEKIGNFYSCVFKNLYVSTARYCCVRLHIDWCQWIGGTISNNGRWASSVSGQQCDYFLIETGHGFHCSGAYLSIPAYNPTERKPNKSVIATAARGSVYSACYMEDTPSYVTILNQWWNDGNEKGFGLNIDCIGSQYRPDRTYKYLTFEEGAFGYYDENEKWVPPAGYGSYPSTNGIDFVRFGSPTHDAGAFPHGGFDFKYGTYGVMYSTNATYPNPPDVDSLRGHKTTKEMFSPYGLMAANGILQLPVLSPALHSNICIWFKDLTGNFDLNNIVLWQGANIDQSTTDVGYDYNLFIAKAEMAIDFGNGYKMAIIQNLKWNNNDGVGTAGSQQSLQITIPNSTPIVLKSVQAFTGGIPVFPTGINYIPESSDQCIWGNVHPTNGFRYNLFKKVGGGIFAPGDLINPWIAHQRANPDYKFGTTFYEDYGYSNLQRLVKGGNAIGSYFAKDFTVSVVSVDEENGRTTIDVPASYRPYVFMGVPLNIVGGSSTSYTGETRIHKRNINSDGTLSGQYVLDTVVGAEGDTLYISQIALAARTYYQDYKTGNLAVNGEVSLNGTRLRFGYGAETANTRTLEFFVNGGTTSTATINAYGTSLGYNATSHVFGGNILFDASSTRNIGSSNNLALASYVTKRMYTATVGDFYGSGSPEGVLTASVGSTYRRVDGGAGSSFYVKESGNGNTGWVAK

>K39PH122C2 cds 8, tail spike protein

MALVKLTRVAEWLGTYIHSLSSVQRLLGSKLDDTLCVLDFGAVADYDTSTQTGTDNTAAFRAAIDAAISKGIRNVYVPGGQYLITGELNLGGTSFTSGEGTRDYWRGITQGVHLYGDGPYSSILVFDAQDEYTPCVSARGGWGTHSPRALSGIAIEPKVWVDYSSTAKGTGVLLQGCCFVPVTDVHIGRFHRGLHLWNKLQGPNDTANTFTQGDFTEFNRMTRVRFFNSDIDIDYQVSLGNNSFHGNSFTDCMAQINSYGGIGMRMWDDGSRNAIRPSSMPYEYIANVYNNKHEINWFGSDARTCYLMHIDKAQGRGCNGDMTVEAAVTLRVVGQYWYQSFGSLHSISAINTVVDGTTDTATRPVAFMWLNSAYPQANFDGGDALLSSSLYPRQFDLNNSGNTGMELLNIRGTSAGAIWSIQNGAALGWILGRRAQSASRPGTRSAWQFSYNGEVIKSVAAANVGIQNQNGAGVGMLGDVLFRPYTGGTVSLGSPTYSFTRLRTTDWNIDTFGIVPVEDGIKNAGSASKRLGTIFAATGTINTSDARLKTDVRPMSAAEIAAARALSSEIGFFRWVDSVDNKGEDAREHCGTTVQRAIEIMQEHGLDPFNYGFICYDSWDEQVELNDETGEVISTIPAGDRYSFRMDQLALFLARGVDARLQALEGA

>K39PH122C2 cds 50, tail depolymerase

MAQSLEGTIQSLLQGVSQQVPRERQPGQLGAQLNMLSDPVSGIRRRPPGEIVWESTIDNPGLDSLFTEYVERGTDGRHLLINTSNGNWWLLAKNGKTIINSGNDPYFVTTVGQTSLQTASIAGLTYILNTEMAPNTTVDNTGRIDPSTTGFFYVKSAAFQKRWNVTVTSAGVDYTGDYTAPAAGSTSGNAEEVSGAYVAQQLRDSLVANGLPAGNVSVRGAYLFFYGLSNCVVSSDAGDTYAVVSNQSRVDQEQDLPAQLPAEADGAMCRVGTASSETAWYQFSYSTRTWSEVGAYGSITKITNMPRELAADDNIIARDWEGRLAGNDDNNSNPGFVENGYITGIAAFQGRLVLLSGSSVDMSASGLYQRFYRSTVTSLLDTDRISISSASAQDSVYRTAVQFNRDLVLFANSMQAVVPGSAVLTPTNASISITSTYDCDSRVTPVMAGQTVIYPNKRNDSYAGILELIPSPYTAAQYTTQDATVHLPRYIPGRVLQMQNSSVTNMAFSRMSGERNSLLVYEFMWGGSDGAKMQAAWHKWSFPYPILSVQALEDEVFLYMQGPSPSNKLLIVSMDPREGYQLGSEYREAYSDLQKQVQVQGGVFTVPAVLRPVGWADNYKEELILTYLPSNPMGPTEVGIKEIAGENTLRVVRGVPDGTYVIGRRYRSTFTLTTPILRDQNDKLVGSGHVRLLRLDVAVRNSGHFDVRVLDTPRDVNWGGELTGILMNSKELTLGQALRMDLATITVPCRTNADTTEVSLFTEGSMELNVLDISYILRYNQRRRRI

>K39PH122C2 cds 55, tail spike protein

MAFSWQESVKPAGTQDIQCDIEYLDKSYIHVYLDGAETTAFTWTSSTNIRLNSPLSAETAVLLIRKTAREYLYIEFASGAPFIEGNVDTQNTQLLHLAQELVEGRSIEGFYGDINMHRYRITNLGDPVDARDAANKQYVDAGDARLDQRIDAEHAAWVAAVANEAAIRKAADDALDVRTTNLEQTYFNANTNSFPWWTVLTADTDTVTPGMPFTKAKVRVNGVTQTAGYSYTVTAGVVKFAEVLPAGTLVDMTIGIDTEADTSAVSSVLELLTAPSGASLISSGNAPVSVLLRRSIFEFMSAGDRTLITSTIGTEVLVDYALAAAVAAGVTELYFPPAPGIYVVGQTPVTLPAGFSITGVAAKPYTASSNASFNSRGTVIRLASGATAPFILTSRHRFLNVILDGRNSSVNLMKGIGSDQTQYCRFDSCGIYRWLNGIGGSSSSGYTATVQVIGCALASNYRGVRNVIDSRFTDCTINANTYNGVELNAGANNNSFLNVRNEWNGAYNYFASGAKRNVVCGELCDRAGLSGFVAVNGGQWVVCGVTVQRSGKNAVVGSVDDAHFQLAGGSSSIIINGVDTLAGANDDGSGTSTPSYTLSTSGANSDEKTFIATGSRLGGFTGTYWLRSGVVKNLSVLGCAGIPDVKNFGFSQSEDGATRLGDKVSSLALSGAGSTATLTFTVSSDGARAQYSEPLVRKLEITARNNTDTGSVSRFYADLIISRELATATVAVDASSVKTHSTLSGGTWGLASASPTGVSVTPTISADGSTLTVTLTAVDNAPRRIWAQLRG

>K40PH129C1 cds 52, tail depolymerase

MATSLEGTIQSLLQGVSQQVPRERQPGQLGAQLNMLSDPVSGIRRRPPGEIVWESTIDNPGLDSLFTEYVERGTDGRHLLINTSNGNWWLLAKNGKTILNSGNDPYFVTTVGQTSLQTASIAGLTYILNTEMAPNTTVDNTGRIDPSTTGFFYVKSAAFQKRWNVTVTSAGVDYSGDYTAPAAGSTSGNAEEVSGAYVAQQLRDSLVANGLPAGNVSVRGAYLFFYGLSNCVVSSDAGDTYAVVSNQSRVDQEQDLPAQLPAQADGAMCRVGTASSETAWYQFSYSTRTWSEVGAYGSITKITNMPRELAADDNIIARDWEGRLAGNDDNNSDPGFVENGYITGIAAFQGRLVLLSGSSVDMSASGLYQRFYRSTVTSLLDTDRISISSASAQDSVYRTAVQFNRDLVLFANSMQAVVPGSAVLTPTNASISITSTYDCDSRVTPVMAGQTVIYPNKRNDSYAGILELIPSPYTAAQYTTQDATVHLPRYIPGRVLQMQNSSVTNMAFSRMSGERNSLLVYEFMWGGSDGAKMQAAWHKWSFPYPILSVQALEDEVFLYMQGPSPGNKLLIVSMDPREGYQLGSEYREAYSDLQKQVQVQDGVFTVPSVLRPVGWADNYKEELILTYLPSNPMGPTEVGIKEIAGENTLRVVRGVPDGTYVIGRRYRSTFTLTTPILRDQNDKLVGSGHVRLLRLDVAVRNSGHFDVQVLDTPRDVNWGGELTGILMNSKELTLGQALRMDLATITVPCRTNADTTEVSLFTEGPMELNVLDISYILRYNQRRRRI

>K40PH129C1 cds 56, tail spike protein

MINMAYSWQEQVKPAGTQDIQCNIEYLDKSYIHVYLDGAETTAFTWTSPTNIRLNSPLSAETVVLLIRKTEREYLYIEFASGAPFIEGNVDTQNTQFLHLAQELVEGRAIDGFYGDISMHGYRITNLGAPAASTDATNKQYVDDLHNDATSRADAQFSRALRVPEAGVGAIPGYVTRRNHILAFNSVGDPIAVLPESGSAADVLIDLASSEEGMGDNLLAHRVVADGAQSLTVHDKLLSVVSVLDFIPRTEHTAIRNKTSTLDLTANFQKAAAYALMVRKRLYIPAGQYYVTSGSFPSGLGVIGDGMGNSVLVTGVGTGAVIASLGYWASGDSWKNTETCVLEGFSVQVPEGNFYAYDSSFDIGYDFSKTTTETPIQLGYYKRLEIRSVGVSWSRGFSILATRNQELLITGCHIDHSMRDAIRALSNEHVMVVNNIVRHCGDDAITVGTNFGGEDLTPWGDVKERTAHVSGNTCIDTKSIRALGAMNTVIVNNIMQRPKESAIIVGGRDGTEGLNDPGFVLVAGNIINDPVSGVLIYAAGGSNTANNSEWNTGITLMARIAAAGVASAVPGQFDYTNMKWVLPETYYLSSMGSESPSGGARGIHITNNQIQRTLPYTGNYSAWGFDGTVFTPTGWVDPDMSTAMRTGKGINVRSNGTSVLFAFMGLCIADNRICGFNIPIDLSPARYLAFTSIIRNEITRYATSGIKLTSGNFGNSARLGVPQGSLDIKGNRFDADPYLEHVNRNTNGTWNSTDYPAGIECSNATGLDIQGNSFRNLVSPVTGRLRFLNNAADPSVNGTGIFRNNTFICQPATNWDDSTIGTADNAGVRFIMSPAENDFIIENSNPLSATYLQVIGSTGAKSAASIPSSGFYVKGTFVKNTVPAIVSETITDSGGSTFVHRYVLNGWIRLTTGTTNISGTDWVVSKSTITLSPA

>K41P2 cds 11, baseplate wedge subunit depolymerase

MIKAPSITSLRVDKLAANFVYLKWDDVGMDFYYVVEFARSRDMDGTIIPDEELIWTQLGYSYENEWFSDQVAPNTRYKFRIQTTHEGFDPSDWVISDELWTFDENAYAYTTMREFTPADSFINEKFAKNNRDYVDFNDDVIMASLMVEDFVYSPLYSDVSQISDKILKQESYHEIQDHIEHVCNDIDRTFLVYSNGLLYLFERFQNMAKVSNDKGQTWYYYKALNDRVGNPVSRTVSYQSTNTTYVLGYDRIFYGRTSTDIRWSSDEVRFSSDDVTFAKLGNQSGLDFDVDSYNTYARLPGGVSKYAEAIACSNEWLYVAAKNVMRRIALRNTPIDTDPGSPTFGERIFDEVSYTIVPGNDKIVVKKMDVLNDRLYVLVTGEVKKAMMDPTVKANVIPSNDAGVYLWDENAKTFTRVYGKTEDERFYITHEYTNMSTNGDEVYISVGNYKYPGTLPDPDLVEKYPEDVHSAVKYDLVTGYTASISINFATVRANQNDPTVWNFGPQEYYNEANFSWHFRDKVSTWITNDNRPLVVYPETLYTLVTDSASPASTIRVNHEVWDKGTVTIYLNNIKFTGFTKYTNGVLLYRSGGRIIGFYELSYRARDELTIFWKPDNTLMVASLVNQERENPYTPDIEPGLIDPDLSHMITRFAPQSYLDNQQFEKFGEYYLQYISLGSNSYYNKLLNLIRNKYPREKNNVEYLWSEINRRNIYLDKTKREAVVRFFESRASDFYSTKGIEASYTFLFKLLYNEDVSVEIESSNSLEYDILVSSTNISQDIVGRTIYTPTGRANVTYIEREYENGQLRWSMTLHNAQGNFIEGQVVKSEKTNFTGMVIRGVRGKQMANNSIDYINRGRSYYVMKIRSNLPTSRYKDDVLRFVHPVGFGFMGITMLTVFINSGLSMTHNETIIDILRNYRFDSGYPKFWPDRIASMDGNGNQQFDLVTGEPLYTVHPRAGEPFDVPPEYDIEEKPLNGVLPSARRFDQSPLFDCSGVKYSQFRNLVDKRLKDDAGNPRDPNPPSQIKVGN

>K41P2 cds 226, chaperone dependant tail spike

MADLEKIQFLRSTVAGKTPTTSQLADGELAINMADYAIYTKNGSSIVQLAGKGIPETNTKKLTVDGPSALNDTVTVAEGKTITFTNENLSGEITRHIVGKCASNDGWYIGSGGTSNNGILEIGTIDDGTETIQFVQRGAGNVEARKLVLLDGSGNTTLPGDLRLTTNKAVKISNGSTLTLEMGVGANDAYIKNLRGSGVLQLTNDGNLTFRNSQVYYATDGRGPGKGGTLLTNVENARQLEQDNFPVTTGTETSWIKVALLKDPGSSQSRLQLMVTNGGNYGNTHSSIDFIDCSARSIPATLTSANISSYLQIRRIGDPGIDNTNQMRYSLVRTSEGLELWFTQRAFIGGVKVALLSIAGYTEYYLPNGYTTSATAPEGLVESVAIRIYDEMNKPNLDNGTDGILSIAKGGTGASTADAARTNLGAVARAGDTMTGNLTINANLKVENPNGTMVDLGSENSDKYSRLTLARKVGSGATVAMLKVTPEGHVQFGYQDAVATPAPSKYILVKSSGLEVEGDLVFNQTYRGTEEAVDITDKTVDLNGLVIKGTDPGTRQMYKCFSSGGGSNIANKPTSDGNFVLEVLSLRKISDTDWTCKQTFTTKNGGVEGTYVRYGQNGSWSAWKEVVAGVQPINLGGTGATSVAAARNNLGVGEWQSVAFGVLNVNGVSNADPTITFKNGAMVREAQGGSLGALILSASASATDSAKYIAFRPFGDSSGTEIRVKANVYNEPSLEWSYGAGVRSSAVGAFVIYAKTGQALHLRPNGDNSNQATVINQNGKMTVGGEFEAQNSKITGNLSVSEDNRMIVLGKNTDIGLVKKSGMSGKMAISKSNSFTIMVSTNTNNQINPADTFNDIFKVDADGNQTVYGNAQINRQLTVSSAATVNGVINANGGIIVPTTKYVQIADAPTQNNQATNKKYVDDKVASAISNAGDTYLPLAGGTVTGRLTITGDQLKTTSLWVTGDAAVNGVLTVDGRARFNQEFIVSTSVNVQNTGNSHLVFRKADGTEKGLIWADEPGSVSIRAGGVSGPTWNFWNSGSCQFPGAISNFNGVNSTTNYPGGGSGAYLNTAGLTSRFSNGAYVSLYFQEYVGNYHQAILNVNGYGRDDNFYFRAGGDFFCTRNGSFDNVEIRSDRRAKSDIKVIENALEKVETLSGNTYELHNTSGGTTRSAGLIAQEVQEILPEAVTQDNEEDGGMLRLNYNSVIALLVESVKELSAEVKNLKAEIEELKSK

>K41P2 cds 227, chaperone dependant tail spike

MADLKAGSTVGGLPIWHSGTFPLVPVGNTLTYRGYKVYTENDKPQASNNDFVSKASGGQYLGVVAFKQGLQINATFTGGSDQNGLYSGDGDGATLSKANIDLVSWYGIGIRSSSGTNGRVLVINARNGDLNTQGNITVEKQISITTANPTDASHATRKSYVDGQINNVTTNANSRVLRAGDTMTGLLTAPQFASTGAASRPEHVPRLDQVITKGTIIDFGSY

>K42PH8 cds 43, tail depolymerase

MHSYRASSVVNQKLKEDQMALVSQSIKNLKGGISQQPEILRYPEQGSLQVNGWSSETEGLQKRPPMVFIKALGQRGYLGDDPYIHMINRDEFEQYYAVFTGNDVKVFDLSGNEYQVRGDRSYITVNNPKDNLRMVTVADYTFIVNRTRQVRESQNLTNGGTFRDNVDALINVRGGQYGRKLEVNINGVWVSHQLPPGDNAKEDPPKVDAQAIAEAIATLLRAAHPTWTFNVGTGFIHCIAPADTTIDSLETKDGYADQLINPVTHYVQSFSKLPLNAPDGYTVKIVGDTSKTADQYYVKYDASQKVWKETVGWNISVGLEYHTMPWTLVRAADGNFDLGYHDWKDRRAGDDDTNPQPSFVNSTITDVFFFRNRLGFISGENIVMSRTSKYFEFYPPSVANYTDDDPLDVAVSHNRVSVLKYAVSFAEELLLWSDEAQFVLSANGVLSAKTAQLDLTTQFDVSDRARPYGIGRNIYYASPRSSFTSIMRYYAVQDVSSVKNAEDMTAHVPNYIPNGVYSINGSGTENFACVLTKGAPSKVFIYKFLYMDENIRQQSWSHWDFGDGVEVMAANCINSTMHMLMRNAYNVWIAEVDFKKNSTDFPFEPYRFHVDAKRSYHISETAYDIETNQTVVNVKDIYGASFSKGTLAICESDGKITEYEPAGSSWDSTPDIHISGDVSGKDIVIGFLYDFQYVFSRFLIKQEQNDGTTSTEDSGRLQLRRAWVNYQDTGAFTISVDNGSREFNYLVNARVGSTGLRLGQKATTTGQYRFPVTGNALYQKVSLSSFNASPVSIIGCGWEGNYSRRANGI

>K42PH8 cds 48, tail spike protein

LVSLLIQLKEATMDQDIKTVIQYPTGATEFDIPFDYLSRKFVRVSLVSDSDRRLLSNITEYRYVSKTKVKILVSTTGFDRVEIRRFTSATERIVDFNDGSVLRATDLNVAQLQSAHIAEEARDATLLTLSLTDSGELDAHNRRIINVAPGVLPSDAATKGQIDEVIGEAGGILQEVQESQKWIKDYIDEFVNDSANLKLVTWVYNNGAAIGGETSITVDIPERVIAVPALYVNGARKDVEWEYEFSPDTKIITLKTPLNKGDFVICMLSESGTPIMNLLNGPTGANEIGFANGRVSDALGCTWISMYGVKLQTVQSIAAGTAVDMTPRIRAAIEAAKNSDTPLQIDLALQTGPSNNKFFYVTEHIDISGLRTIRGPLYLMANPDTMKETFLTSDAVPRPCMLINMNAKYTDGKIHFSTTTGGQQFDEIQTSFVKDPPHDRWIGQLHTTCYTHFRGQLWSRRCAMRFANTYDCTFGAQVAAVDAGSINYYAFQVGNYPWYDKVDESNSLTFPRVLVHSNKYRDILIEGSKTNINACHIEAAVVGPLTGLVQNTYDKQAPGGIATAVFGPTGGQIGVLNYNIHVNSASRGCLLVNTMGCNVSHIFTDDRADVVVGDIFYLGRGGSIGSIYTRSDVKTIGGSGLTIGNIRCGGNLSNTAVRTTIDAAYVGGHLLVNSGKINYAEIGGNATQDQYGSIHGGNCKGSFTMANSSLLENFTINGPYVSTAASPTVIRCTFNGSFKNTGGGHFERCKLKAFPIESKLSPTYYRCTLTGTVTSGVAGARSYFDKCSVGSYDFDNAQDVMVTIDGGSAASMTQQNYSGAILMTGGFRLTGSSVSIPGFKAPTTLSVGYGAMTINPYSGRGYVLLWSGTQAQWRATSLID

>K43PH164C1 cds 35, tail depolymerase

MALVSQSIKNLKGGISQQPEILRYPEQGTLQVNGWSSETEGLQKRPPMVFIKSLGGRGYLGEDPYIHLINRDEYEQYYAVFTGNDVRVFDLSGYEYQVRGDRSYVTVNNPKDNLRMVTVADYTFIVNRTRQVRESQNLTNGGTFRDNVDALINVRGGQYGRKLEVNINGVWVSHQLPPGDNAKDDPPKVDAQAIAEALATLLRTAHPTWTFNVGTGFIHCIAPADATLDIFETKDGYADQLINPVTHYVQSFSKLPLNAPDGYMVKIVGDTSKTADQYYVKYDKSQKVWKETVGWNISIGLDYTTMPWTLVRAADGNFDLGYHDWKDRRAGDEDTNPQPSFVNSTITDVFFFRNRLGFISGENIVMSRTSKYFEFYPPSVVNYTDDDPLDVAVSHNRVSVLKYAVSFAEELLLWSDEAQFVLSANGVLSAKAAQLDLTTQFDVSDRARPYGIGRNIYYASPRSSFTSIMRYYAVQDVSSVKNAEDMTAHVPNYVPNGVYSINGSGTENFACVLTKGAPSKVFIYKFLYMDEDIRQQSWSHWDFGDGVEVMAANCINSTMYMLMRNDYNVWIAAVDFKKNSTDFPFEPYRFHVDAKRSYHISEAAYDIETNQTVVNVKDIYGASFAKGTVAICESDGKITEYEPTGSSWDSTPDIRISGDISGKDIVIGFLYDFQYVFSRFLIKQEQNDGTTSTMDSGRLQLRRAWVNYQDTGAFIVSVDNGNREFNYLVNARVGSTGLRLGQKATTTGQYRFPVTGNALYQKVSLSSFNASPVSIIGCGWEGNYTNRASGI

>K43PH164C1 cds 40, tail spike protein

MDQEIKTVIQYPVGATEFDIPFDYLSRKFVRVSLVADENRRLLNNITEYRYVSKTRVRLLVDTTGFDRVEIRRFTSASERVVDFSDGSVLRAVDLNVSQLQSAHIAEEARDAALLAMPEDDAGNLDARNRKIVRLAPGELGTDAVNKNQLDTTLGEAGGYLADMKELDKIIRDFIENFANDPASLRGVVWVYNAGSAIGGETSVVITKPGDVLAVPCLYINGSRQDVGVTYDYDNRTKTITFKGDRVLKQNDLLIAITAEGSVPLADILMSLDGAKYVMTSQGVSVEAAIRGIRGNVKSLLDLYQAEDGDDYSPAATRALAQGNALYIPVGRWTFKSTVQIPAGTTIFGSGDKSIIASPEASDAAGSALVTVLRADNVGHISLKDFKVDGGSGAAHTVKKNVRGVRFYKCNNIRVDGVEVSRTADWATSFEQCTEVYVDGYKHRRSESPLHGGRDGLHFLDCNGFMATNLDIESHDDCVGITSETLGTFDGHIDGVRGTSTIGSLVIYNEETSGGVYIPMPMRGLRIDNVFPKEGQTARQLVRVAAYATTSDARDVIISNVQGNVTASHGVQVQNVKNVFLDNVDAHVVSSNSHGVYISDCDVIRGNVSGSTKASTHDGVQVFKSKNVELDITSRGSAGNAAQINGCTNVRVVPLFSSAGAAGLRITGSTNVDVPSGTFSGTFPGLCINQTGNTNLFIASTILYDTASGRINSPVASMFMDIPAVIISAKEAADGTLIVYRAIGGTITRLATGRYQITFTNPMQTAQFTFTMTAAHPGSVRQAYAASAIGTSGLTLGVKDSAGTDTHSEYIRFIAYNY

>K43PH164C1 cds 41, tail spike protein

MDFFTQPKGSTIGVLRDGRTVQEAFDSLMYTEVTLKSGRENAEANRVSLQAAADSTKLISVPAGEYFVSAHITLRTGNVIVGRGANASSSGYTRLVCETTGEGVFWYTGDTSTGQKRMPQIYNMGLKGDYPIRFNDERTAVIADQAASNVPYGMVPVVQFCTIDPLTNGVGIGISASKMFDGVFSFNEIANFDTGVLLNGCDLMYVAHNRIRNAYKYMVLELGVGTFGSQNEIYHNDILHAGSADCIFIKSTARHVRIYDNYLEQASGTSGQALIGFIDASVVDAPVFNGNAAAVRASTIIKDNRIDGQHFAKYFVYKYQPLGQTYGVIEDVSTVGPNTGLGANHLVLVDASGVTIDRVPFLYNSVQPCSFRFSGPRFGKWNGYNSASDYALKMTGSNMSMWGTSLGGNNLKDYLSARGNSLILSSGFTASAVLQFPSGTLIRPNSQYAIKVTAKCSSGSEALTFAGVANGTGQTSVTMQLSTEPVSATAQFTSGSTQSGFSLGRSNNGADIEIIAIEFIKLYSVEYSVASDTGTVTVYRSTGDIVISAAGNNQFPAYTVLRYIGGSLKEVYKSVTDAAVIGIAWSVSGQTLTVNITGSDGGKRFSVTQEEV

>K44PH129C1 cds 9, tail spike protein

MVGALSKGGIMALTKLSAALVRLASGGTLQEAWDTFRGNRVNVLDFMTRSDRKKCINYETGTDVSYAFKAAFDTGSKIVDVPPGLYYAKDVTMPNKTKLIGEWAYKPYNMVSDASFDKDGTMIRKIAGAENLFLWGTGCSANNIMFDGVDRKSSAIMSKTGGKISVGFFKCGFYRWARVGNKNGGYLGCSMQFCNINQNNVGLYNTVDGNHVCLTINANKSDGVRLEKGADSNTFTNCRNEWNEGNNWNFYGCVSIQVVNELCDRAFAYGFRISNSNVQLVNVDVRRSSKNASSTATSAHFYIENSLVKMIGVKTVSGVDDTGGNVVDVSPAYIFRMDGANQGELTLIGCKLTGSTTGLISGTARPAKMLVSSCDGWPDYCNYGLYRKNQGRQYKDYVTASGKANETPLTLSFSTVDATARTSLAQVEGEGYSSRAIGTYSNDIMRVELLWRNTTSGGSNGAVVWLLFKREGGSATVSILSVDCKSNVIGDADVNGSPLDGQLYTVGASATSADASTFNLTFKCKSDNTAGFQVRGYLM

>K44PH129C1 cds 10, tail spike protein

VPINKGGYMALTRATRPYVPGIRLLGEGTLDNALTFAVPEMFSVPKDGVHSANAGLMAMFNADYNNFRLTKGTYLLTDTLEIVFSKDVNIVCEEGVVIKLANNVRKHMLHFYGDRVHNFSWEGGELNGNWEGQGGEVLVSGHVDDVSHGIVISRWNVAKITDFFAHDCMGHHINHAGNNYFIAEHIRIDSHISTNFPAGGARGDGITGVSKNVYINDVSGYSSDDLIGIFPGATWIPGETDLNYLQVESIIIENINPKSKVDASGVTRYTWHGVTGGSWNGVNVKTVVIDNVVGEVQDGGVRYRCSPASGDDTILNGSADHISITGVHVYVNGKTTDQYETVAVMIGSHQQSTTLGATPSYFKNVHISDITSNTSDNLRTVIMAGHATIDNLNISDVSVNYTKPEHTASHVTLTGSNIIGRVHLSNCASHHQGVVSDTVMNARPVVRCSLNSNGVTTIRGTNLAVRRSSNGEAWIGSVLFTANFPNKVALFGEDLVLNAPNNFASAHPARGCHFTDRFLGRIRRDHILDGWVFEDACTQWDGSNFGRPNAANFTGYALVKDWKAGTLIRTTGSPWGECAGWVCVEGGAAPKWALAKANFDDSESRMTSTTTPTQLTPRMLITTALINASGFPGASGTLLTYTGAARTDRAGAYQEFTPASGTKKYMRTWNNGTSTWNNWLSVTFA

>K44PH129C1 cds 45, tail depolymerase

MMEVQGSLGRQIQGISQQPPAVRLDGQCTDMVNMVPDVVDGTKSRMGTTHLNKLMGSGDDNMAFHHYRRGDGDEEYFFVMKPGAVPEIFDRMGNKCNVVSSDAPMVYLSEVQNPREDVQFMTIADVTFMLNRRKVVRTRDERSKKVGSTALVFCAYGNYGTKYQITINGQVAAEYKTKDGGEASHVETIRTEVIAEQLFLKLQQWDGVSQYDIYRMGTTIVITRLDGNTDFTVNTEDGAKGRDLVAIKYKVTSTDLLPSKAPEGYKVQVWPTGSKPESRYWLVAEKSEGNLVTWKETIAADVKLGFDKNTMPYIIERTGIVGGVAQFRIRQGDWEDRRVGDDLTNPMPSFIDDDVPQRLGGMFMVQNRLCLTAGEAVIMSRTSHFFDFFRYTVVSALDTDPIDIFSDASEVYQIKHAITLDGDTVLFSDNSQFILPGDKALTKDNALLKPATAFEVNNHVRPVATGESVMFATSEGSYSGIREFYTDSYSDTKKAQPITSHVNKLIEGNIISMVASSNINRLFVMADKNRNVVYVYDWLWQGTDRVQAAWHRWVWPTDVKVRAMFYSSETVYLILERGSQGLFLEKMDMGDAIEYHLEDQIRLDRKALISFAYDAASDGWYSSALPWWPEHPEMLECVLTQGWPAYVGGSFLFQHEDDGNRLHTTFDLGEENSIIHCVVGEHYSQEFEPTPVVIKDSQGRTSYIDTPVVGLVHLNLDKYPDFTVEITNASGRKRVAKASNRIGGARNNIVGYVKPKEGVFKFPLRAKSTDAIYRIKSVAPHTLQLRDIEWEGSYNPSRRRV

>K45PH128C2 cds 227, chaperone dependant tail spike

MADTTQFEQAVDQVIEDSERLHKVVNGSAIDTVIVEDGSTIPTLRKALLDNVYFKTPPQPWAAGTQTTVFNQLYSFTSSAGTFWWYAPGASPSTPVTLPADPSTSTAWKVYNDSVVVSEKFAPLNTPAFVGSPTAPTPAQGSNSGAIATTAFVNLAVAAAINSLTGSSPSYAALTVVGASTLNTLVVSGISQFGGTIDASGVLGKFQKISLSGQTATLSFDYTAASNYLKTIISPNSVQTNNLTSAVIVNGTASADNTTMSLTGVGNNTFDYVYIRGNSSKASTEPRLKVTGTTELENVRVTGSLSGVNVGVDGLDILPNSIVTTTTAEIGSDLTVNGVTTLGSATIQDLGVVSSLTVDGNSTLTGGFTAGSASSVAGNLSITGLLAVTGAVTVSTNLTVTGNANLNNGGTGTTTVNNLNVLGTLTGVSVDVNGKNINPNSVLATTTIEAYGSLKGASLVITGEATAPKVTANFVGTIPGSVTTPSAGTTWTPGGTGSDLTKMNNIYNVDVTNTLTIGPWANLGSAFTATIYLFQDATGGHAVTLDASYKIINGGTISTAANSVTILQVTYCGRGTVYDVAVYQRP

>K45PH128C2 cds 232, chaperone dependant tail spike

MAIPTIPLQIWAESDVVLPNAHTANKISPIADLWDKGWDLGEKPACEELNYVLNMMTWWMTYISEEQIPGLSNDYLRKDQNLSDVSDIPTARSTLDVYSKGESDTRYVNVSGDTMTGALTVPRITFPSDSSDTAHITTTTGVDQVYLDFVIGDNVGTAGQPSVDVMRFRFVPVNNSDSVSPFNMMELNATSNGVALLKVQGNITATGTTTTGTVSSTTINNGGNIQTTSLGVGGTATLQNLVVNSNNATIGGRSVVRAVNSTAANANGDVSISIGVSDIRWSGEQNKVQVDFENYGSSGRFARGPDGSVLTGLIDANASGDLYLHDIDEIRFRFLQKALDGIWYTVGL

>K45PH128C2 cds 235, chaperone dependant tail spike

MSSDLPVIKIRNLSDKVLVSDTDELIIQSSVDTEKTTISKFISDIGILKKGDIMDVSGASLVGTHSGSTVQQVLDSLSNKWQNLSNGYYFATSGNEVGTYVDTISISPVQRSYGVQISTSLSAFSPKRDNFISNGTTALRWNQVYAVNSVISTSNKKKKTNLRQISTAEIKAFYEIGKLDSVWQWLAKYSSEKGLARLHSGPTVQDAIKIMLKYGLDWTKYSAFCYDKWEADGDNPAGEEYAFRKEELLFWILRATIAVQEDLDKRLSVLEDSLSGN

>K45PH128C2 cds 237, tail spike protein

MLQSVKISELPSADTLTEDDLIVVDQPDDTKKATLFQVLNHLEDTVEQSVLVELAQPTGAGKSGLMKGGTVQDALTFLTPGMVSSDPYTNFGTAMIAAFQQAMAQGYGEIRIPAGVYTLDRTIDVTLSRSMTLVFSPDAYIYVTSPMTAFKININAKHLNITANGARIMSNWGSTTSASPVYALHLIDASLDKSCSVTDFKVGYVGASKFDYAIRNESINLGTFHRCLLQGKNGIYLESNLANGASAHAMGCQFIGNEIYVDADVFTIANQGALGCEGLIIADGQYITSGTAIRISNVGLASSSYLPPLVRILNNHINAYQGLYVKDVSRLFVVGNDFQTKYNLTTVVKGIIELGGVQVFEHHGNAYSAVGYGGADATYKATPVYQFASTLTNAFFNSTGNVYWLPGMTKPAYDFESAVNVTRVLSANETLNSAGTWVSSSYKNLITLAPHASIGNNGAASGLDYSSDATFSGGVLTLGSTPPQGFTYNIGTSVVPNSSVITQIVSPAGMVGKEINLLFSAAEVTFTHGVNMICPDQKSAVMSIPNVVKVFAFNTTQTRIMDIGGNTTRRIDITSAPTSKTSAGYHGAEFFDTATKRYYKYFVGYGWVYTTMTDIP

>K45PH128C2 cds 239, tail spike protein

MSVTQTWRTGVPTPGVLRTYPPNPLYVDTVRGSAAGTGSLNNPVNSLSLALGLCAGLPDYVIKIAAPETNPLRQEVIFDTSMNVFLEGIDNEPWHIYGSEMHTSGWALSGNIYSKVINYTSVMQVVVTTMTETIADKDDFYLKLVQNTTTPTTPGLGQYGYSGSTIYVRLPDDSDPNTHQIEVSRRNFGVGTVGFGLLTVRDVVSRYCMINGISCGLSTQPAGTGFLTVYDSVVEYCANGGVGGTGRNERIECTNVKAYRISNDGFGQHAPTGGAGLMILNGCDGSYNGDAPGQSAQGASNHETTTMILNGGTFNHNVSGGMVVIESGTCDIHGDTEYGPVVMDGNMRFGNTAGTIANQAGCAWLDNAKGTVTGSVTVKNGLGIGVKRASTAIVDGISNIISENNAFPDQL

>K46PH129 cds 24, tail spike protein

MALYREGKAAMAADGTVTGTGTKWQSSLSLIRPGATIMFLSSPIQMAVVNKVVSDTEIKAITTKGAVVASSDYAILLSDSLTVDGLAQDVAETLRYYQSQETVIADAVEFFKTFDFESLQNLANKIKADSEAAESSAAAASASENAAKTSETNAKASENAAKNSEVAVEATRDQVQQIINEAGEQSTLVALSQPDGIYRSGWKRNPITKAISKAGERLDAMPISIWEQQFVDLITDKPTSDPNTWDWSPAITAAINYAADWSTVPMGTATRYSSWYLDFTPGKYIVRSQVKVDFSSMTFDAGKPLIKIRMNGAYISSAIDKDHAVWFMGCRIDVDEFRFTKEGNINAYYLKLGSEQTNNTTAVGGRISDLRCYYPTKGITFGQGYDLVIDETYMSGFTAVDDDDTENPATAIHFLSHANDNCNNIVFIRIHLETSMTANYVAIKADDNQAAGQPHHNIKFLGGHVEPHFRGAKWFDLGVSNEINFDTVIFTDNGSNEVEPASYNLGMLATSVQRFDGCTFQTNNLSAVAYNPAVHKSLLKFYPGNATSREFNTCYFATAFANVSGAGNFNAAIDATATTNGNNSYRPNNCSLNNFNRRVSGGNIYSDLAITSRKYLVGVDSADSALKWTYNNTDMSYNGGTVVMSLSTSGLLTTSGGLRTGGNIASGAFLQAGVNNTAAGNRDVSFYPYGNNTMSARILADNGGGLTITNNSNAINYVANAGVSAHIFTGTIRPNVKATYNLGSSTITFNNAFLQNAPTIVSDENHKELIQNITDELLDVWGTLDFQMWKMKAAIAEKGEDDARWHFGMIAQRVKEALTNAGLDWTRYGLITYEKWDAQEEIVVSWDDEYEVIPGSPAFYDEEGNLIQEAVEERRVLIREAGSMVTQEARDAGEIYMLRMEECFAVEAAYQRRRMDRIEAAISALNP

>K48PH164C1 cds 43, tail depolymerase

MALVSQSIKNLKGGISQQPEILRYPEQGSLQVNGWSSETEGLQKRPPMVFIKSLGPRGYLGEDPYIHLINRDEYEQYCAVFTGNDVRVFDLSGYEYQVRGDRSYVTVNNPKDNLRMVTVADYTFIVNRTRQVRENQNMTNGGTFRDNVDALINVRGGQYGRKLEVNLNGVWVSHQLPPGDNAKDDPPKVDAQAIAEALATLLRAAHPTWTFNVGTGFIHCIAPADTTIDILETKDGYADQLINPVTHYVQSFSKLPLNAPDGYMVKIVGDTSKTADQYYVKYDKSQKVWKETVGWNISVGLEYHTMPWTLVRAADGNFDLGYHDWKDRRAGDDDTNPQPSFVNSTITDVFFFRNRLGFISGENIVMSRTSKYFEFYPPSVANYTDDDPLDVAVSHNRVSVLKYAVSFAEELLLWSDEAQFVLSANGVLSAKTAQLDLTTQFDVSDRARPYGIGRNIYYASPRSSFTSIMRYYAVQDVSSVKNAEDMTAHVPNYIPNGVYSINGSGTENFACVLTKGAPSKVFIYKFLYMDENIRQQSWSHWDFGDGVEVMAANCINSTMYMLMRNAYNVWIAAVDFKKESTDFPFEPYRFHVDAKRSYHISETAYDIETNQTVVNVKDIYGASFSKGTVAICESDGKITEYEPTGSSWDSTPDIRISGDISGKDIVIGFLYDFQYVFSRFLIKQEQNDGTTSTEDSGRLQLRRAWVNYQDTGAFTVSVENGNREFNYLVNARVGSTGLRLGQKATTTGQYRFPVTGNALYQKVSLSSFNASPVSIIGCGWEGNYMRRANGI

>K48PH164C1 cds 49, tail spike protein

LVSLLIQLKEVTMDQEIKTVIQYPTGSTEFDIPFDYLSRKFVRVSLVSDDNRRLLSNITEYRYVSKTRVKLLAGTAGFDRVEIRRYTSASDRIVDFSDGSVLRATDLNVAQLQSSHIAEEARDAALMAMPQDDAGNLDARNRRIVRLAPGIAGTDAINKNQLDTTLGEAGGILSDVKELQGEIYDYITKFADDTAMVKGVNWVYNSGVAIGGETVIKITKATPVFAVPYLEINGSRQFVGWQFSFNALTQEITLVKPLVAGDFVVALTTESHLPLEDLLYGTTGAGSIGTTSGETVQAVLDRLGDVESPEKYGATGGPDDSDAVQRAIDAAAARAKVYEGIITPEVVILLNIYRIKKTLTVDGSRVRLVSLTSAGGLHFDPTGSYDNLRCIVVNGTAPNAAYVGQLKGFADGVRFTSTGKTLTLFHAVRASSNSGDNGACLHNIDKCAFRGFKDIFTHGAGGWGWTFNSSQFSGNDTLMNLVTAADTYERHTFIGCTWQNGGYAFNMANPDGKVYWIGGSIDYCDGLALISSGHLETSGHNEWTARTKPLVRITGDNASVVSSGTMFIRNNTTTPYVIFEQYKSRQVAVRDLTFVTDGVNVARGTLSNREVLKSNIFFANDTAKGIAYNSADEPILNGNRAAADITLSPSVNHTYTVQDSKITVTANAGGSSAHLYVDIFIGGSQKVALKMRCTNGSTTGPVFLNKSIMTAGKATIADLTSYGTTQWVAGASSDGGTVTVIDVPKQAAFLRLDFNLVNLTTATTFTIENLNLFTA

>K49PH164C2 cds 24, chaperone dependant tail spike

MATISDQLAADIHNAFSKYYTDLANQDQIFFGVGDVQITKQDGTTATVRSWNKVIGSVDTAAQRGTVNTFTALQTFSAGINVSVGNINVMNDNSMIILGKNSDLALLKKQGQGGTIAVGSGTPFKIQRASTATVAVSSAMEDIFVIGVDKQTTLPGALSAGGNIDNTSKGKVLTQAIELSMSTPYIDFHYNSSSADFTARLIQDQANRLTAQVASFWVQDGRVTASSTAPSNPASGAQLTGNPVRSMLRGRGAYGDVDGAYSQFYIEEQVGTEHRMVLYLDGYGRTDAWLFRAAGTISTPKGDVMTQGSDVRLKEGFTEAPASACERIERLGVCQYRMKGESRVRRGFIAQQADTVDKVYTYQGEEQEIEGEKFRVMNVDYVAIIADLVASVQELRQELKELKGE

>K4PH164 cds 22, tail spike protein

MVGALQDLYAIKHAADIKAGIAGIGVVEESLEAMKPRLSDRIFYALNKTGELVSQAVRVGRVTRRQKTGYNLNSWPQGKLCIDGNGRIYCGYNSAPSHGGSGTVPMITRSDDDGVSWSDPVQIVTGENYARGTDWWSLGVDDSNNLWGIVRSRGANNQVGVTFYNLYKSTDGGTSWVKVGEISSVTQVINDVSYVPELFHDMCYIPTTGRMVTGYHFANSSRVGFMSFDITDPLNTIVTQDVITHGEFSTLKYCEPTIAVEYTRNAEGTIYGGLRTQDNAYPSQLYFMNTDLTGFTRFNAPESVQYCPMTIRRINGQFVLLTIERYNTGAMNLWFGTPTDFYNMASSNFWKMPIGKIVDEVTVGASNVGVQDMEIHGDNLYFAWSSETKNTYADTYIGKMNMVYPASLINNEYLEGL

>K50PH164C1 cds 28, chaperone dependant tail spike

MAIYRTGQASMDAQGYITGYGSKWREQLTLIRSGATIIFLSNPLQFGVITEVISDTSMRAVTTNKAVVPKVDYVIFLHDSITVDGLAQDVAETLRYYQGKETEFAHFIEFLKEFDFKKLEDLTNQTKQSAAAAKVSETNAKASEGKAKTSETNAKNSENAALSSKNAAKASQDAAKASETAALASKNAAAASQTAAKTSETNAKASENAAAASKNAAKTSETNAKASEEAAEASKNAAKNSENAAKASQTAAKTSETNAKNSENAAKASQAAAKTSETNAKNSENKAKEYADIAQGISSPMIQYNWPVGTGANERFVKIAKLTDPGSSESHVTLMITNGGNYGARQGSIDFLDASARSLGTTVINASNVRQFMQIRRLGDPSLAEDNQLRYGVVKGDGFFEIWAYQRAFINNVKVAILAKAGRVDLYIPSGYVSQEGAPDGWVKSEAIRVYDEVNKPSRSDLGLANVMEIGAFGLGGNGISYSDITSNADLMQRMKEKGGHFWRASQKSGSTSNIISHGSGVFSRCGDTNSAINIDYNSGKVVILAANDSSLAAGNVKVNTLYGTANKPSKSDVGLSNVTNDAQVKKAGDTMTGDLDISKGTPSIRLKSASGNAHLWFMNADGNERGVIWTPENSDSLGEVHIRAKTKGGTTGGDFIVRHDGRIEARDAKINYKIAARTADFANDDTNTGSTNLRVSGKQHTPVVLVRDADSNLSIGFKLNNMNQKLLGIDVDGDIAFGENADQRQNSKIVTRKMMDAGFSVAGLMNFTNGFAGTWEAENINDRTLDLNSLMIKKSNPGSIYVYQCISQDGGNNITNKPSGVTGNFILYVESIRKVSDTDFTNRQILFGTESNREFTRYCSNGTWSAWRESVVSGMNQDVSVKSMSASGRLSGGELAVGGAGALNGNLGVGGGTASKVPSSDKGIVIGRGAMVREGGEGRLILSASGGTDRQVQLRPAGATASDNGIEISCTSASGGDTKFAFGQGAAIRCNASGSPIISAKAGQLMYLRPNGDTSSDGQVTIAGNGKMVVNGEIESTGDMTCARVFSKGAIQTESGGIELYHASPFIDFHFNKSASDYTARIINDAADQLTFDCRSVRTLRDFTARGSIRGCYNDAFVAWPIEDPTGGNGTILKAPSFISRFNTIGNAARCAMWLEEYRGYEHRAVIEVSKWGDSGTTQYWQFKANGKISSTTHGDVVFAGLSDINYKDNVVDYDGLQSLENIKAMNLVKFTYKDDEQKRERRGVIAQQVREIDPCYVKESDASYQDADGNVVENKRLVLDTNPLLMDALCAIKALSAQVEELKEEIKKLKGE

>K51PH129C1 cds 9, tail spike protein

MGLVKSVYSGAYENDRALWARALAEAGYILVAGSFSEGATLSSEYEAVWDSVGGGCYRWTGAFPKTVEATSSPSDDAGFVKVTTESTLRNDALTVVPASRFGVTGDGVACGEACVAAITYIMNNGGTLQFPKGEVNWGTTRATFPIYNGPEFKIIGADGGTKFTFDDTPPYASGVSWPYTEGTKIVIGGTPSTTGLYVAPFTLSGIDFDYSRQVNKGGPTLETMAAGAHPTPYSDGALILRVMYADTPILENINISNVYGSGLQVWKCTDATIRNVNCTDVSANQVLGANNNESVDHFGYGIWSGASANTLIQRCRAWNTRVFECDDSLVSPNNGAKYNGTICGYIGIYCEYAPEQGDVGRYPPRYSWTGDTSLTPIQLRGYGRVEGCTVSGYTLNYKSESALSIQFINCVSLNHYIGFSMQSSGLLSACYANALGVANNVCPQNGFEAQRADYHLSWWSTSTNTHDLLMTGCHAVTTKYQSVAVGKGSPIIESNVFDISKSARVLNSVTSFQVGILDFNKNIIRADSNLETSSGHIRVTNTHMPNVKGNKIYNASSVRFSVSLQRGNFNENLVEGPLNLYFSGPVHCGKNLLRDGATYTTPAITYYRASDGVFEDNDVYVYDAADAQAQLVLLSSTTNFKGLNNRVHVTAATGARAASIPVFKTFGQCLYTDISDNSVSGDSASAYPWNLFNGVSGAFVFTCKNNRTDNATATLLTGLYSQRAPWRLEGNDWTQTFTAEVNTEANLYSAYKAVLGEKIPYIRPVAGGAEGIVKTSSGWKTYGSVAA

>K51PH129C1 cds 56, tail depolymerase

MAQSLEGTIQSLLQGVSQQVPRERQPGQLGAQLNMLSDPVSGIRRRPPGEIVWESTIDNPGLDSLFTEYVERGTDGRHLLINTSNGNWWLLAKNGKTILNSGNDPYFITTVGQTSLQTASIAGLTYILNTEMAPSTTVDNTGRIDPSTTGFFYVKSAAFQKRWNITVTSAGVDYSGDYSAPAAGSTSGNAEEVSGAYVAQQLRDSLVANGLPAGNVSVRGAYLFFYGLSNCVVSTDAGDTYAGVSNQSRVDQEQDLPAQLPAEADGAMCRVGTASSETAWYQFDYSTRTWSEVGAYGSITKITNMPRELAADDNIIARDWEGRLAGNDDNNSNPGFVENGYITGIAAFQGRLVLLSGSVVDMSASGLYQRFYRSTVTSLLDTDRISISSASAQDSVYRTAVQFNRDLVLFANSMQAVVPGSAVLTPTNASISITSTYDCDSRVTPVMAGQTVIYPNKRNDSYAGILELIPSPYTAAQYTTQDATVHLPRYIPGRVLQMQNSSVTNMAFSRMSGERNSLLVYEFMWGGSDGAKMQAAWHKWSFPYPILSVQALEDEVFLYMQGPSPSNKLLIVSMDPREGYQLGSEYREAYSDLQKQVQVQDGVFTVPSVLRPVGWADNYKEELILTYLPSNPMGPTEVGIKEIAGENTLRVVRGVPDGTYVIGRRYRSTLTLTTPILRDQNDKLVGSGHVRLLRLDVAVRNSGHFDVQVLDTPRDVNWGGELTGILMNSKELTLGQALRMDLATITVPCRTNADTTEVSIFTEGSMELNVLDISYILRYNQRRRRI

>K52PH129C1 cds 25, chaperone dependant tail spike

MALYREGKAAMAADGTVTGTGTKWQSSLSLIRPGATIMFLSSPIQMAVVNKVVSDTEIKAITTKGAVVASSDYAILLSDSLTVDGLAQDVAETLRYYQSQETVIADAVEFFKTFDFESLQNIANQVKADSQSAGASATAAAASESAAKTSETNAKASENKAKTSETNAKASETAAKTSETNAKASETAAKTSETNAKSSENKAKTSETNAKASETAAKTSETNAKASENKANTSETNAKASETAAKTSETNAKASETAANSAKTDAQTAKGQTQNLRDQVVDLVAGVQAPDKLSGVASSSESWMKIANIKSTGSAYAFVQFIIGGGSDYGSANVPVDIFSLSGIGLPASPLTSGNIDTWFTQRTLIAARPNAKRVNLGAVKKADASYDIYLHAPGGWIPNLWLNLVNVQANNGSITGPIIDRTGYSWITTKPAGIVYNSPSDYLLANDSTIPRTNVANTFSQPQAISAPGGNATLTLNGCTVRANNSGVLVLSTPSGSEGLMFRPNGDTSVNGQVTISGNGDVLVNGAVKSKGVDVTASQNLPLKETTATTGVGVNFIGNHTTECSFGIENTAGGSAVFHNYTRGASNSVTKNNQLLGGYGSRPWLGSDYTEHSNAALHLLGAGDASGTNHGGWIRLLVTPKGKTISDRVPAFRLSDNGDLWIVPDGAMHSDLGLVRSFETLNAAVPKFNGPTNQDGRGLKIVSSDGAPEINMIAPRGTNNSSPAVRAMWCDGSLGNSDKYIGAAQAWSTFFLGASGHDGEKFDSMRGAVNIQATEGWGKNSTPTRILFETCAAGSTARTARWCVDHNGNFVPMGDGGYDIGWGSGRVNNIYAKNGAINTSDGRMKNDVRAMSDPETEAAKAIAKEIGFWTWKEQADMNDVREHCGLTVQRAIEIMESFGLEPFKYGFICYDKWDEQTVVSEYGPASEDGSENPIYKTIPAGDRYSFRIDELNMFIAKGFEARLSALEDKLGM

>K53PH164C2 cds 24, tail spike protein

MLLSALLNQPQRTKGMALYREGKAAMAADGTVTGTGTKWQSSLSLIRPGATIMFLSSPIQMAVVNKVVSDTEIKAITTNGAVVASTDYAILLSDSLTVDGLAQDVAETLRYYQSQETVIAEAVDFFKDFDLATLQALVEQIKGDAAASQDAAAESQGAAAESQGAAAASQQAAAASQEAASDAKAVRDEIQQIIDDSGEQSTLVALTQPDGFKHIGEANYSYIRSYTGNAEKIKVSGFAVAGDGGHGVFDLIDDDDHTIIDDNCVHLRDALGRLWKRRFLGNTIRMRWAGAKSMAETSAPQDAAFENCLLAAAQLSDSGYPQSIIAGVPRGVVYLAQRHYIRCGNFPESLSWKYPTTGGGDRFGFDMTCAIGEGAGFFLVQANNPSVRLRVDNSQVAFSTTNYTDEQIATLVQNNYILRMESMVNAPDLHIHAANYPGTVLYSTGKKDYSAVTAQWPDLVSVLPSIQAVGDVVLNIKTCGRDFYFTNTGAGMGHWNSVWTQNNRVPGYISNCYDLTMTYEDYVPHNETSGGLIFSECGTMSLKNILIGAGGVGHLCIWDSRNVHINRNIAICGNTTYAASNTADDLYALEVCNSEVNISGAHVQNSGRFMRVGFNSQVTFQHMTAWDVSTLVEMTNNLSRLKYRGQRVLAVLDPSRVFFNGGFAQNLNASQFGWPCAPIVKIDETVGGDFQFHWNLWTNNAHAGYADKTEAELYFIECKSTSNTGLVSFGEAAKLEGNTSNYFVYLQDKNQLGPVNTRATNFVRVRYGDTSQSSFAMREASHPLEGTTFTARSMFSYPYRRPGRHTVGFTISGGSVSVTVNGQVRHRYTSDGLHTLALDLRLQEQVLIITTGTVGTSMPQWRYILEN

>K54lambda1_1_1 cds 225, chaperone dependant tail spike

MADTTQFEQAVDQVIEDSERLHKVVNGSAIDTVIVEDGSTIPTLRKALLDNVYFKTPPQPWVAGTQATVFNQLYSFTNSAGTFWWYAPGASPSTPITLPADPSTSTAWKVYNDSVVVSEKFAPLNTPAFVGSPTAPTPAQGSNSGAIATTAFVNLAVAAAINSLAGSSPSYAALTVVGASTLNTLVVSGTSQFGGTLDASGVLGKFQKISLSGQTATLSFDYTAASNYLKTIISPNSVQTNNLTSAVIVNGTASADNTTMSLTGVGNNTFDYVYIRGNSSKASTEPRLKVTGTTELENVRVTGSLSGVNVGVDGLDILPNSIITTTTAEIGSDLTVNGITTLGSTTIQDLGVISSLTVDGNSTLTGGFTAGSASSVAGNLSITGLLAVTGEATVSTNLTVTGNANLNNGGTGTTTVNNLNVLGTLTGVSIDVNGKDINPNSVLATTTIEAYGSLKGASLVITGEATAPKVTANFVGTTPGSVTTPSAGTTWTPGGTGSDLTKMNNIYNVDVTNTLTIGPWANLGSAFTATIYLFQDATGGHAVTLDASYKIINGGAISTTANSVTILQVTYCGRGTVYDVAVYQRP

>K54lambda1_1_1 cds 230, chaperone dependant tail spike

MAIPTIPLQIWAESDVVLPNAHTANKISPIADLWDKGWDLGEKPACEELNYVLNMMTWWMSYISTEQIPGMAADYLRKDQNLSDVENKATARTNLEVYSKAEGDNRYVNVEGDTMTGPLTVPRITFPSDASDTAHITTTLGTDQTYLDFVIGDNPGVAGQPNVDIMRFRFVPVNNSATVSPFNMMELNATGTNTALLRVQGNITATGTMTTGTLASTTINNGGNIQTTSLGVSGTATLQNLVVNSNNATVGGRSIVRAVNSTAANANGDLYISIGVSDIRWSGEQNKVNVDFENYGSGGRFARGPDGSVLTGLIDANASGDLYLHDIDEIRFRFLQKALDGIWYTVGL

>K54lambda1_1_1 cds 233, chaperone dependant tail spike

MATIELPVIKIKNLSDKTLVTGSDEIIIQSSTDTEKTSINKFITDIGLLKRSEVTGITGASVIGTHSGATVQQVLDSLSNSWQNLYYFSTTGNEIGTHVDTIALSPVQRTYGVQISTPLASFSPKVDNVLSNGTTSLRWSQVYAVNSVISTSNKKKKTNLRQITPTEAKAFYEIGKLDSVWQWLSKYSSEKGAARLHSGPTVQDAIKVMLKHGLDWTKYSAFCYDSWEANGDTPAGEEFAFRKEELLFWILRATISVQEDLDKRLSALENSLSGN

>K54lambda1_1_1 cds 238, tail spike protein

VVKCTNVKKRGKPTMLQSVKISELPSADTLTEDDLIVIDQPDDTKKATLFQVLNHLEDVVEQSTLVTLAQPDGFKNIGQCANLTVLRSLQFSSVGKQVFLKEHTTGQNAGGGIWYCHSLTSDSSYVDDNGCQIINDYGQVIRRKDVKELTSSYFGLQAGDTIDTVLTNMYKASRTFNIYEAKIENPGFDKGYVLQGGLRFYCGDKPFYIHSYSIGTLRGPNIWHTGNNVGVTFSRFKEDGTSQQAWSGGGIKGFRIWGAASYLVQGNTGADSTPVRLSDMWQGEACDLWVTGYTGNTNGAVVSLYNEFAWTEGALVKNIMVRQSLRGLTFLRKHGTTATDSFFRTVADISFNAGVSGQSTQVMVVGDGTAAGECLVYGHDIKLTQWMSAGSWHDIVRLEDYSIIAETGVIKIVADGYGISKTTVPATEVVHSINVRGLNARFRSRVENWSNQAGGWGLDFLNIIFQSSMYTNAMTFYESDFDALPTINPVGMKVRFNGTFTVAERQSGKVYTLNGLIPGMTLKVKLTSRNGNDLNDAVVQEWKVFVRSTNLPCIVVPMSGSANIATTDGLAVTNTSPVQTATFLKTVTPTQARNFIGQNYGLTVKNANDDNSLSYALNSGRKIRFVLPANPTATTTTPYSVEIEVL

>K54lambda2 cds 23, tail spike protein

MALYREGKAAMAADGTVTGTGTKWQSSLSLIRPGATIMFLSSPIQMAVVNKVVSDTEIKAITTKGAVVASSDYAILLSDSLTVDGLAQDVAETLRYYQSQETVIAEAIDFFKDFNFEALQNLANQIKEDAESAESSAAAAAASENAAKNSEDNSKASEVAAETARDQVQQIINDAGEQSTLVVLAQDDGPKNIGYKNSRFDGVYPSSLLDIAYNKISLLEFIPVNLHEGIRNYTYTGDLSVYVQKAIDTANAMGGATIMCPAGQYYMNVKTKPNVILIGARTNTVRRAMPWAFTVAASPGSTRFRNYSGSDWIIKSATSNQEDVTSRSFGIIGIDFDAKDATNSTGGVRLRGPEFCVKSCSFYGFQDQGLEVSGNIGLIEDVIANECLKNRVRSDYVGTIEVSGASDCQIHRIEGNAQVKGLNSITNAEPYICGIKIAGNNHYISGLMGEISETGVYISASSVHHKVSDSRADNNVGPGYLLNGVQMVNCHSYNNSRSGDGLYPAFGAVAGSTRALLTNCLAWTDAAPVDGTGEQRRHSYGYDFSNIDYSSLRLKPWLNQCFSYGHSLGWINAPGNYGLQYTPTVGLLSVTSDATTTPNVDGVSVIAINTTSLIQITGLSGGMIGQQVDIYLNATATVTLVNSSAFLVNNFAKNSNKVMVAGRVYKFIKTAANVWREVGDTPRFYSGDTASRPSSAAVPGMQYFDTTLNKPIWRNAANNGWVDAIGTSV

>K56PH164C1 cds 43, tail depolymerase

MALVSQSIKNLKGGISQQPEILRYPEQGTLQVNGWSSETEGLQKRPPMVFIKALGQRGYLGNDPYIHLINRDEFEQYYAVFTGNDVRVFDLSGYEYQVRGDRSYVTVNNPKDNLRMVTVADYTFIVNRTRQVRESQNLTNGGTFRDNVDALINVRGGQYGRKLEVNINGVWVSHQLPPGDNAKDDPPKVDAQAIAEALATLLRTAHPTWTFNVGTGFIHCVAPANATIDLFETKDGYADQLINPVTHYVQSFSKLPLNAPDGYMVKIVGDTSKTADQYYVKYDKGQKVWKETVGWNISIGLDYTTMPWTLVRAADGNFDLGYHDWKGRRAGDEDTNPQPSFVNSTITDVFFFRNRLGFISGENIVMSRTSKYFEFYPPSVANYTDDDPLDVAVSHNRVSVLKYAVSFAEELLLWSDEAQFVLSANGVLSAKTAQLDLTTQFDVSDRARPYGIGRNIYYASPRSSFTSIMRYYAVQDVSSVKNAEDMTAHVPNYIPNGVYSINGSGTENFACVLTKGAPSKVFIYKFLYMDENIRQQSWSHWDFGDDTEVMAANCINSTMYMLMRNAYNVWIAAVDFKKNSTDFPFEPYRFHVDAKRSYRISETAYDIETNQTVVNVKDIYGASFSKGTVAICESDGKITEYEPTGSSWDSTPDIRISGDISGKDIVIGFLYDFQYVFSRFLIKQEQNDGTTSTMDSGRLQLRRAWVNYQDTGAFTVSVDNGNREFNYLVNARVGSTGLRLSQKATTTGQYRFPVTGNALYQKVSLSSFNASPVSIIGCGWEGNYTNRASGI

>K56PH164C1 cds 48, tail spike protein

MDQDIKTVIQYPVGATEFDIPFDYLSRKFVRVSLVSDDNRRLLSNITEYRYVSKTRVRLLVETVGFDRVEIRRFTSASERIVDFSDGSVLRAADLNVSQIQSAHIAEEARDAALMAMPQDDAGNLDARNRKIVRLAPGIDGTDAINKNQLDTTLGEAGGILSEINEVRGDFYEYLEKFAEDTSMVRGVVWIYNSGSAVGGETVVKVTKPTTVYSVPYLEINGSRQEIGYHYDFNPATQEITLAKPLVAGDFLMAMTTESYLPIESLLASPVGAEAIGTKSGKTIQRILDESQRYFTPEMFGAKGDGLTNDTKAIQDAVDAAMSSGGGKVILKGNTTYLFDHLLIAREKSPQSRFEARLIIEGSGGSVLKHTGSTTESASAFLVRGVLGTGSLADIYMRDVVLRDFSINGTVTSTANGLVLQRATAVRIENIYVNDFGGNGYRALDLYDSTIDSLEVQRCGIVPGATVGAYGMYITGAYDQSNANHYIACRVEMCPLIIAIDKGCRHNYFHNCKFEQGRVNPTTSNPVYINDATELAFEACQFVQNYDSDVRFLVVTSNLFPYWVTHGTEKVVNFTDCSFVCSRSVTAYWVDVAYTTFTACAFSSCNGGSLIPLSLGKNVFFTDTKVVVRTPEGNVLEMKGSHSRVTNLKVVYFQQPTSGVFIKFTNMPTLSDVVVDGFTFENFEPFAPYSGHSDFMGDVVVTRRTGYVYNGTNDRVIYGCATLSYSGATPATWNNLRNGYNGQIVVVHAKTNPVTLDVSGGLIITKTGSNVTIPTNCVSALVNISGVWRQLY

>K56PH164C1 cds 49, tail spike protein

MLKNDFNQPKGSTIGVLRDGRTIQQAFDKMRYADTLQELRTMEPVGTRDVATVRRATEDSVLVNAPVYYDKNDTTSPDDGISVFVTAGGARWKFNTFKGYCAGLAGLKEDGSNVTTVVNGIVNRIVAKMVAAGRVNNQQRTVNIPVTGDAPEWKLTGPLVWPTVISLVFHGSVLLDGTSLTAGKILNCNNVPFMDRLTVAIMAAGTNLTDRPGRVNQAVGRAALTCIGGEVTVRLPGTQMDGSNNPTIHTVGAMIGNDAACLLDARDIYFEKFNIIGAKTGIEFGCYNTFMCGVEHFNVSRCYDGFSSPTLGSNYGERMYLRNGTIGNMDRHGAYLVGGGDFTLENVSVDFLGGDLAHFGPLSPAEYKHLSGHIEGVKGSLAAKEMPASYSKALVILGKAVRRDDRVVDQNDYRGVRQLFACPQNPYGRMLKVINESYAPGREGQVPNNPYPCETGWPGNSGVELILPKDTFVDTPYVNSYSPAVRNSVNQTISFTTASTGPLGGNILSTDYAFAAEIIGDATCSYGTPAEATSDGYMPFIINLTSPSDVVYLFCTNRFRPGSGQLVLWGNCSVTLVGTTGSIILAPVIASYLGTTWTANTTTGAVTATPIRRGIQEGGTVDMSALAAAGGIPNGTYQAMPSRPVQGFYLGCDHAVAGFKITGGTGQVRLKLPVWWFR

>K57lambda1_2 cds 92, tail spike protein

MNKMFTQPTGPVAKQTNKQAIARIFSVRQADIGYINEHILVDDYTLLYDRITEATWYRGTATGTPLSWSVTGSKLTLTTTTGTYELTKAAVVTEASLKSPSAMESIYTPTGGTLASLLLRTVSPAEFGLVNDPTGVNAVKNTKAVQDAVRELNRLGGGRLLFPTGQYTFCYGIWLFSNILVDLNGSVCTFKDATFPLRSRACFVMGSSYESNREKALEFYEDGSFANNPNPTNTAYVNVPLGTFLRDNPDKVEIENCHIYNGHLKATFTSPTANGAYAVNMVNAQYCSAHDLTFDGFTEAINMGSDTNNETPSCHGCSAYDLVVLSPDQNKTYYSIGFISNSTNCAISRARQIAPCKDGTENGSGVATNVVEDCIIEDIFIPSLGITQSSEGVLLNNAKGSLVRNICVRNCKVVVAVFYVGTNYISADKWNHVTGITGKGTTALIAIRGKYTTFSDFAADSTTPYEIYFGTSNGSNNRIEQEPKTIYFTETSTIKAFWYCNNNYIKGWEVKRLYLRPAQILLNDKASVFSFNANKSVAMNDDTTIMFLYDLPPGIKALKAVKLFAYYNTNSVAKGSSITIAVRRMVAFDGNANAGPYVEPGIAATSNATTDMRDISMSAVHSDTVSPGMIRMDDTTNGLANNLDVLVTFSNTAKNSLMKEWEITYVG

>K57lambda1_2 cds 93, tail spike protein

MDMNSHNPFNTGGNGCPNDFRSGDQLVDRIIGDAYHVVKEVYLALGNLTYIYNYLQKYGLIITVDSEEAIKDIPLSIGKFARIYNKSETAGYYFTDYLYVDDDTSGIKPNEPGATGSWVSTKATGSNASFVRIWKYVGEYNGQTTIQLPTDIPIIDVQTIYVSGVRQDSGDGFEYNEGSKTITLADELEIGTPVTVVLGITDPDLGLDIFSILAKSDAASTIGAVSETNEPSTVADELVAARNKLKAFEDALAKPDGYKLVGSVASFSSMRLMTGLVPGQRVKLASYYSGGNTGGGEFIVKTGSAVDDAGHICVPASQSGIYLERRTSTVKFSDYGITTESNSSGPRVDQSDKVQAAINRAKSLKYVLDTEIASENHYIYTGIYVEKGIDITGLKTIRGCFSLLFDSTKIAGLSAPGYSDVQWALVNLNAKFDTAGIIFGTTVGNQSFDSIVVRDVSNRGASCGGQLHVTSGTMVSGALCATDVNGPGVWVLGSYDSVISDIRTVNCGNALLWAIDIAGYRGSRVDNTNCMVISRIESHDSLDRALRCNADLSDIGEIHVEATVVASDAVSSSATIADNGWGYANVLLAGLGTTYGSVRDLPVSGSIPAVFELVADDTSIGELSLPRSSLSIHYAFTTPRGTLSAGTINLSGDVIVKDGANTNIDALTVTGASSKITSSSTNFNVGILRATGANASFNGIGGRYGRLDCAVVFLTGSDVTRGSITNLTLGDRNTLTQVAAQTLLVNGTRNYLSDGFRVSGVSTLNGINNAATNAVFVGVVTLTDPWKFDKVYINANLVYTGNTTSTLYDVSFQDVFIAGDLVLSGACRIHGHNLRVINLRIGAMSEGFIVLNNCVVAGGVEGNYVGSFNVPAIGSITQNFTTGVPYVYTPTGWKLLTAAA

>K58PH129C2 cds 40, tail depolymerase

MAIALLVESSDVNQKLKEDQMSLVSQSIKNLKGGISQQPEILRYPEQGSLQVNGWSSETEGLQKRPPMVFIKSLGDRGYLGEDPYIHLINRDEYEQYYAVFTGNDVRVFDLSGYEYQVRGDRSYVSVANPKDNLRMVTVADYTFIVNRTRQVRENQNVTNGGTFRDNVDGIVNVRGGQYGRKLEVNINGVWVSHQLPPGDNAKEDPPKVDAQAIAAALADLLRVAHPTWTFNVGTGYIHCIAPAGVTLDEFQTRDGYADQLINPVTHYVQSFSKLPLNAPDGYTVKIVGDTSKTADQYYVKYDASQKVWKETVGWNISVGLEYHTMPWTLVRAADGNFDLGYHEWKDRRAGDDDTNPQPSFVNSTITDVFFFRNRLGFISGENIVLSRTSKYFEFYPPSVANYTDDDPLDVAVSHNRVSVLKYAVSFAEELLLWSDEAQFVLSANGVLSAKTAQLDLTTQFDVSDRARPYGIGRNIYYASPRSSFTSIMRYYAVQDVSSVKNAEDMTAHVPNYIPNGVYSINGSGTENFACVLTKGAPSKVFIYKFLYMDENIRQQSWSHWDFGDGVEVMAANCINSTMYLLMRNAYNVWIAAVDFKKESTDFPFEPYRFHVDAKRSYHISETAYDIETNQTVVNVKDIYGASFSKGTVAICESDGKITTYEPMGDSWNSTPDIRISGDVSGKDIVIGFLYDFRYVFSRFLIKQEQNDGTTSTEDSGRLQLRRAWVNYQDTGAFTVSVENGNREFNYLVNARVGSTGIRLGQKATTTGQYRFPVTGNALYQKVSLSSFNASPVSIIGCGWEGNYSRRANGI

>K58PH129C2 cds 47, tail spike protein

MRRGGSMLDRLNQPRGSTIGILKDGRSVQEALDATSRVVTLVNAAGDGIADDTMNVREALAESVRTGYPLSVTGGKKYLLSDAVDVASNIVGDGTCLFIQTAPSKAGLRIVADGVSVTGVKVTPSSEPSSIHHAGIVIYEANHCVVTRCNIVYRGRTDREGHGIGIFHGNDNLIALCTASGANLTEGLEHPSGGNDFFVYGNSSRNTVLYNTGIGKNVRGILQQTSISGQQCNYNTYDGNTSIGHIGYGHICYELVHDASTKMYGTVFRNGFVANISGSARDPGTGKKFFGAGVYNQEGQETRIEGYLIQNVCQDADIEETLPMGGVSSTRGDVTVRNITIVGSGRCGVKITSTGLADDARTPTVKGLRVRNAAREALYLVNTNRAQVEDLNVTASGRIIRATANSAYGNSHINISDVVAVGSKGILLSGFKIATVNGVQGVGCAGLLSVDNTGTLLASSLSSDSGTGTAFSIASTVASGFLRDSVAIDGATGFNLAAQITYERLRAEGNSSNFTGRYSSIVDGGSDAELQVKNIQYLSVGTGADITSLVGGVVNQVVTIRVTAARTFVHNTTSLVLKGGANLAATPNTVVQLWCIGNSRWIQI

>K59PH2 cds 46, tail depolymerase

MKVQGSYQSVVLGVSQQVPHERRSGQMWEQVNMVSDPVRGVARRWGSVFLDARDVRDTTMSEADMKKAVNLYREAEFTCDGRDLVVLYAKGNTVPYGLPPVMAYDITQKKTLNVQGDGPLWDLLKTNGASSRVNIGRFLYLSVNNHNTTFTVNNRIKNMLDPKAGTLWIRQGNFSRKYEVTIVKPDGTSEVFEYTTPPASYPGKLDTSDIPIPDVPTNPGTDDGWSPSNTLLQQYQKDLAAYNKLISDRTNAYNSAVTNWIGEATEKMQPAYIAQQLAEKITAAGHAEVQHVGAYIAWSSASNIADAVPSENSDGTFMRAVAYQVDAVENLTPKHWVGHIVKVAAKKQDQKDAYYMEAFRKTEDGETFTDVIWRETAGQQTVPGSMFLHCWADMNTLYVASSPEGLNALAPAAEAPGYERSSVGDALSSPAPGFFGKPINYMGVFQDRLLISSGAVVFASRPGDYLNWFRKTVLDVLDNDPMEMYALGSEDDTISWDTTFDRNLVLFGRKNQYLIPGRQTMSPKSPYIQIMSSIKDTVLAEPKASGSYVFYGKDTSVKGSVHQIQIGAVSDSSDSYEISQALDNYLQGRPVQLVTIQSPNNVVIRTTDYNYGLYIYTYLQNMNGGERVFDSWSRWEWPKALGMCIGMCDDDGNLLVFKLRNNNGKTTLQAEQFSFETELPDNGYLDCNRPMDLGVPRESGRAAHYIGGHPYYLMGSRLDKIDSNMSWWEEDMEHLRTGFITEAYVVPTSPYMRDRNDNPITTGRLTLGSFKVNVEQTAGLYGQVTTADRTYDMVDYGGRLLTRKSNQVGRVPLVDTSLSVPVYREIREFQLRLQARDWLPLTITGLEWVGQWFNRTQRV

>K5lambda5 cds 196, tail spike protein

MANKPTKPNFPLGLESQDQSVFQEGILNNGVVEHGPDAVMTVPETPDASGVPSAVRYNEDDDQFEGFYNEGGWLPLGGGNGGRWEALPHASSSLLQAGRAYLVDNTDGVSTVLFPSPKRIGDSVTVCDLYGKFSTYPLTVDGNGKSIYGSADSMTISTDNVSATFTWTGEARGWVITSGVGLGQGRVYSREIYSQILSVETSSIILSTQPSIVDVYVDGKRLKESLYTLDGYEVKFDPALASGSDVQIIQYIPIQLGAGGGGSGGTVITWEYNGGAAIGGETQIVLDVVVDSVSEIYIRGQRQQIGRGFTFDPATSTITLADELEAGDDVVVVINGDPTVYNQIDRTPWEVARANNVSNSEVILSTDKTSVLDGKTVLYDVATQTSWGIPSGIAAGVRIISVDSGVLKYKENDVEKTIQLDLPVNFSPNLELRLRSLGGAAIPVLQTGESVQDAIKFVTPLLKSGIENAEYNYSVLQNAANKNKPLDLPKGDFYVSQAFQAGKGQIIKGQGNPNFSPNCYTRLICMAEGGGCIWYTQDTSTGQVRMPQIYDMGLTGDYPVRFNNEQTAIIKDDISLSNVPFGMVPKVINCAINPRVNGVGIGISWSKMFDGIITCCEVANFDIDVLLNGCDLNWVAHNRIRNGWRYMVLELSASTFGSQNIIYHNDVLHAGSPNCIMIKSTARHARIIDNYLEQATGTDGQALIGFIDATDIDAPSYAGNTSAGRYSTILRDNRIDGFSKAKNFVYKYQPKGQTYGEIVDVSTVGSNVGLGSNALTLVDETGATVDNVPLLYNQQQPCSFVFKGPRFGKWNGYTSEGQFSDVINGLNVGAFGTSLYGNLMSNYLRARGNEIVLMAGFASTGIFSYAASAGMFEPEKSYIIEVEAYCTSGTEDFTFGGIVSGTGKVSTTFTLSTTPKRMRVEFVTGISGSSNGIYFSRSNNGADIVIKRIRFLKKYLNENTMSASTSKTMQITTQSGEIHIAANGGNQFPAYKILKFINGVLVEIFSQATSSSVIDISWSYSSGTLTVNVTGTGGSKQLAISQTSQPG

>K5lambda5 cds 198, tail spike protein

MGYFQMTRNVEEIFGGVVVAPHQIPFKYTSTNGGETFLSLPFYPITGFVTINGGVQVPLDNFEIDRNTLNLGRELEAGDVVFCLFDKIMSPQDASNSAVRIYKFLSVGGETEFTPDFTTYGVQSLYIDGKYKTPGEDYNYFKTSGKVVLDAALPTGVWVVAEMSIKQNIPALAGNNGASEIGTETGKTVQETLDDIGYNLASVEKLMTDVTDMSPVHTHGFYKSMDGGHGDWYKTGNTDLTKAGTHDVTKALVYNAKGTEYALNVAVDKISVLANGAKGYDYADCIDKTTDDFVCLGQVINGIESHLTLGVSIANNIDTYDGGKRLNIIVPTDGYRIGKEAAKLYSGVAYKLNAARIFVFAGASYQYSITGKRLNGFQHGVDEITEKWEAVNRGIYWGSVSLQNTSIHGGTLFGDHTIRSLRDDCSSGLGVLVLNPEGFSTHNLTIRETFMWAMVETTAEVEATEFNQNGHAFDNDSIDYKYIVDAWVNAGITSRFGNFNRTTHYNSKFMGGRRGTYRNGCDWSGLYNCEVTNRLAWRNAANVSGEIPEYIAVCTGTALDVSGGYWGPAAAKDYNARYGTVYSTAQNHSFKAVYTEWTYNFITISAWGFNGKASRLQGLHLDVVSVYKDNFMDYSQIRFEGGCFGTVDDGGNYTYPAGFTHYDTPNGQTQFSYGTPVRDIGAFRHTGFDFKYGPYNMYLTAGTDWDSWRDRPYAKEMFNPYGMQINSGTAFLPWQIPAIKSMVCFWIKDLTGNFDPRNIVAWITAASQDGSANTDEALYKSFAEKVVDFGNGYKMLMLAQKRLSAWDGQYTFARNANITITVPAETPIVLKAVEAYTGGIPIFPNGCGNYTPESNGASILSPVTNQVGLDGSLGGGLFFNGDIIGPWVHMRRTASGYRYVPSLTAGYTLSRKMVTGGYALDAAYKVAFNATITAVNSNNTTIVEVPTAQLPYIAVGIPLYVTGGSSTSATGTFHLIKRIANADGTSSARYLVQGVLGAVGDVLTVDQNQLSSYTFFNDMNYNALTAATLVLTGTSQATAHRSSVSAGIGYNGASGTKAMEWYFNGGTTPSHRLVATTVSGMTLEAGGNFSVVGNTFPSADGTYSLGTSSSRWTQVFATNSTIATSNRDKKTNIRQITPVEVSAFYEIAMLNSVWQWMEKYQVEGDDARLHSGPTVQDAIEIMDKYGLDWTKYSAFCYDKWDARDAVIETWDAEYETIPGTPAEYDEEGKLIVEAVPEVKVLVREAGSKVIEPALEAGEDYAFRKEELLFWITRAIVQKQKDIEIRLSDLESKFK

>K5lambda5 cds 199, tail spike protein

MNPQFSQPGGSVSKDVNKQSIARVFGVKMSEVAYLKAGLVVDSYKVLYDKTTQTCWDVQNSTGSVISWTTTPDTCVLVTSTGTFSLSLSYANPKLKEMVSHLIKLIGGVITPEEFYSEGETDHTNALKLAIQEGITTGKKVVFNPSIKYKVTSTINVTLTELQQLDIDFNNAIISFNGNVQPFIFQNTLVGGVVNTTIEEVSYNLSVTGTTNSRIHKLTAPGHSFTAVGQIGRIFSDDVVDDTDAANQYKAEYFVVAAIEGDVVYTSGLLFETYTNNIKICRPSNAKLKIGNYNFHSNWESSTNKSAMTVRGFVFANSYGVNYCEWLNGPCINVTACYMFEQTGSLTGYNLKNSSADGAFGYLFNDSGSFGTIINTIKSVNSRHPYTTSTGGAASGAIPGDGRWDLRGRTAFSDINMLIGQGDGCTLDSHATSYKININKVIALSDPRGDNVGGAAVQIRGNSLRIDSIEVQNCKTGLFVSGATKTSDSYISIGQIRVASGQGCLPLDLQGNGVNRVKIIIDNLDFNTTNDTVISVSNANVIVKYFSGDFNPYQNGGVLYNVKGTGVLDILDGDVYINTGSSHSLAVHSDSNTVIRAKLRATGMSRVSYLARSASQYDIQSEWDVHLDTWTGTPFSGIPASGAKVFAKIRSGATIKPLAYRALTNLLAGNNTIDLQNAGDPVLFLRIQTSVTGVVINGLSKGALPGQMLVVNNHTNSTDALTIASAGQMLATGVITTLSPGMGMSLIWDGSNWRIA

>K5lambda5 cds 200, tail spike protein

MKTQFNQPQGSTSRETNKEAIARIFGLKKSQVGYLSTTTPIDSYVILFDKETQTCWYRGTATGTPINWVVSGNSISLTTENGIFSLKLLDIFKNLKSTEPGMGVDLIGGSRKYFKVTDYPGGTPDSKVSRNGNGTLTIIKGTNNTSAIKAALIDAQTYNGIVYFPSPENGNAYLYDDTLYPAVTSGALWRGASILGDGKTATKLIFDGGDKPAIHVKGTSGWPTNIFLHGISLYSANDFVGEGWKLQGITGIRLSDFAAYRFGDGGLSFSNGSAAGIFTEFNIIEDGWLENNKTNTKFRKDGGDGSFHGITMRNVISNNLTGQTGLDIGTGCVIYNADWNQVTFFGASGVQWILNNGSRNGFETLYFEGDGTVTNNGSWSTAGYWRVQNSTGAIVDTSTIPFFNEGYITPTSPTDANFSAAGFTSLESLKPLISNQAYRGLMRLRGNNAEAIAVTGYDSGTFENQGLAIVSQSLGDGIKDVILRQLLHLNGITSFRPEYRFNYIGGSTQLSINSNGRHTGVMGKRVVGSITANAAAQTISLSGLLPVNDQAYDVVLVVSTPGVTSKITGVFTGMYDTTTNTTLKPVDNTRTAGTELSISASSGVKITSGGLVSITLTATVDCTYSLVVIGKGAY

>K60PH164C1 cds 94, tail spike protein

MNKMFTQPTGPVAKQVNKQAIARVNGIKQSAVGTLSTSTPIDSYTVLYDPLTQTSWFRGSALGTPTSWSITGSILTLVTSLGTYNLIQADLRSNLNSSAYPGTALVRGADGASLDEAIKFVTPLLKAGVENAEYNYSVLQNAANKNKPIELPKGEFYISQAFQAGKGQIIKGQGNPNFSPNCYTRLICMTEGGGCIWYTQDTSTGQVRMPQIYDIGLTGDYPVRFNDEQTAIIKDDVSLSNVPFGMVPKVINCAINPRVNGVGIGISWSKMFDGIITCCEVANFDIDLLLNGCDLNWVAHNRIRNGWRYMVLELSASTFGSQNIIYHNDVLHAGSPNCIMIKSTARHARIIDNYLEQATGTDGQALIGFIDASGVDAPSYAGNVSSGRYSTILRDNRIDGFSKAKNFVYKYQPKGQTYGEIVDVSTVGSNVGLGANALTLVDETGATVDSVPLLYNQQQPCSFVFKGPRFGKWNGYTSEGQFSDVINGLNVGAFGTSLYGNLMSNYLRARGNEIVLMAGFASTGIFSYAASAGMFEPEKSYIIEVEAYCASGTEDFTFGGIVSGTGKVSTTFTLSTTPKRMRVEFVTGISGSSNGIYFSRSNNGADIVIKRIRFLKKFLNENTMSASTSKTMQITTQSGEIHIAANGGNQFPVYKILKFINGALVEVFSQVASSSVIDVTWTYASGVLTVNVTGTGGSKQLAISQTI

>K60PH164C1 cds 96, tail spike protein

VDMNSHNPFNTGGSGCTNDFRSADPLVDRIIGDAYHVVKEVYLALGNLTYIYNYLQKYGLIITVDSEDAIKDIPLSIGKFARVYSKSDTAGYYFTDYLYVENDTTGIPSNDPTATGSWVSTKATGSNASFVRIWKYHAVADGETVIQLPTDIPIVSVQTIYVQGIRQDVGEGFTYNEGDATLTLADELDAGNLVTVIIGISDPDMDIDIFEVLKNTDGASNIGTMTGDTVEERLTSLDSSLNSLGSGEGLKLVGRCASLSALRLIEPDVDGQWIVLEKAISNGQIINEVLTYDAADTVSPDNGYSVFVTANGRRWKADLSKGYNPLFLLGAFGYESISSCINKIAYDLAVLWGNKKGVIDYCTTIRIPGMPHGETRYNVTGAIHIPSFVTLKMDVDTYFDYYTVTNTDGVVIDNSYFPMLLDSAYDTSSTARPRSVLLWESERDIFIGGRLTLTHPIGSARTSNAGITIGNTVTGFIDVRGCRVRNFESLGFYYGIKINPIDNYINTFDGFHLGRNNYGVAVLGDTKNNAGERFLFRDGTLADSDSDLIYVENNAFELFFHGCSLDYPTGDLVKITKNGNTYISFNQCHIEGVQGMLVNVTASTTFPKYGKKIVFSNSILDLGSGQVAPALWNKTWAFSSVINTYLLIEKSCRVWTSTSTMSHAKTAYQSLIVSGQPANNAIVIDFPQNIEDLLDASTVLLGKFNPTGEDKRVISTNRFSGTDGAAYSPSVSAADQWGWYAVNGSWTYSPADASDNDGLQSISLTSDNASNVYYLICALPYSVMSQEKFRALGAIKVASGYTGNVNVSCLIEARSLLTMNSSAVSETTLATTYSPAVDVAAIAEANGRIDKFQGFCTQAGQVNSFSNSTHPMQYVRVGLRITGFTGTISLKLPTVTSHRLLGS

>K61PH164C1 cds 9, tail spike protein

MSLQRLSTSLIRRALGGVLERTLNYVCPEDFGAVNDRTADNTAALQSAWNYARANDVKFVARGTYRIFSPLYFYGKVQADFTGSRIIVDPSVVAAAAITFDEGGMIIQNMDKAVIVRGLQVMRPLVSTEAGGRINFHADTNSNMDGVVFKQANNMKCYDWGVYGFRDALSFQGKSCYLNHFFGCNLGMLWRYGVSIWANDDSNENYTFVGGSIFNVNNPTFNGTGFYIDSVASQVDIGFRGTSFDYCDISMYLRNSYVECSACHFENNNNNAHIICEFTSAKAKPTIILKGGTIGGGPGITYWTGIPVESSQGRPGLIEVRAGGSDNVIVDRVVTGQFRVGETVTQIVKVVGNNTALLQHLSIKPLIDSGNADTSTTARGIRLSDALNFLRVTNSLDGWTQNNILNNGEADASVSSDYYDSAFPHSRLLSGTANRNFSLTQVIPIRSGENFSIQYYYKMADYVAGEYNSRVDYLDQAGGVIGSSIKVRTGNAGWTHIWHNGRAPNGCVAIRFQHNCTNFNAKVYGTKEFLWIW

>K61PH164C1 cds 10, tail spike protein

MALTHSTSLSHDRLRSVASYADLRLRVPRYVGEEVYLESHVGGRGVGGGLFVAKAGTASDDGGTRCVTPSTTMYWKRVYDGNRVHIDWFGADPTGSSYSSDAIRAALLSSSFIEMQGVYKYNGAPVDMGTFDIEGNGSTILFDDGVQFIVCTGTIGRPWMNNLYLKGGKGYIDFRGATANSFNDIRGFMDLNMSDYTGTAIRLTDMDCPWWVIKRCMFAGRTNEGTIGLWDNGSDNNIVTECKFYRNQYHISTKLGSGTYRISNNDFGQFFTTGDTVNRANIWVRVPTTTPTSTGVTGMFLVEGNKFGNENERSNDVKLLLANVGSDGMPSYTSYSGGLPAMHFDFKVNYYTGYGNYPAHWMRSVGGWMPQTFSLQDENLQYADISKGKYFCYVDNVTRTTPYLIYVKRKAHRDSQVLWRGNTSNDPLITFSLIDPSLSVSAGDPECHSPYSAGVGNGTIDITNQKMPNVTYAGENMTITASTDFLGRKEAVQGDYTTRFNNIFQNLSAVTSYQPGYIQGEAKLSDDSAFESAVYLINYSKNTGDRVVYAQFPIRLRRGQWTSYCFPVMFMPNEADHVLVLKPNTQDTTSYPNKVIWSRSRAFLGKTPGLLGVQRMGGLILTELPTAATDLPVGSLWVDTASSNTIKVVL

>K61PH164C1 cds 52, tail depolymerase

MEVQGSLGRQIQGISQQPASVRLPGQCTDAINCSMDVVEGTKSRPGTVHIARLGDLGLIQDNTNIHHYRRGDDVEEYWMITNPLGIPDIFDKQGRKCTVTETEGAASYFNSNNPRVDYKFFTVGDTTFVVNRTKIVRARADKTPAVGGTALVFSAYGQYGTNYQIIINGVKAAEYKTASGGSASDVETIRTEVIAEQLYTNLLTWAGASDYSISRMGTTIVISSLSGASFTVDTEDGSKGKDLVAIQYKVTSTDLLPSKAPVGYLVQVWPTGSKPESRYWLKAEAADGNLVTWQETLGADEVLGFNGTTMPYIIERTNIVGGIAQFTIKQGYWDDRAVGDELTNPMPSFIDQSLSDIFMVQNRLCLAAGDSCIMSRTSYFFQFFRQTVLSAVDTDPIDVFADASEVYALKHAKVLDGDTVLFSDNAQFILPGDKPLTKATALLRPTTTFEVDTNVAPVVTGEAVMFATKDGAYSNIREFYTDSYSDTKKAQPVTSHVNKLIRGGIYHMASSTNFNRLFALSEDNRSRVFVYDWLWQGTDKVQSAWHKWEFYGATIGGLYYSGETLYLIIKRNDGVFLEAMYMGDPLLSGSDQVRMDRTVTVSLTWDEATLSWKSSPLPWVPTQVEMLEAVLTNGNQAYVGGAFLFEYNPSTRVISTKYGLGDTSQILAAKVGQMYKVEFVPTDVIIRDSQDRVSYQDVPVIGLVHLNLDRYPDFTVEITNRKSGAVRVAKASNRVGGARNNVVGYVKPTSGTFSFPLRALSTDVEYRIISISPHTFQLRDIEWSGSYNPTRKRV

>K62PH164C2 cds 25, chaperone dependant tail spike

VNINQIGVCAMATYKVGKVKINGNGLMTGTGTNWTAANALVRVGATVVLATNPVRIYTVGSIISATSIQLSDWGSDAAITTDTNYSILLHDGLTVQGLAQDTAETLRYYRNFESTLGDAAKATIGEAPGNVMKVGAFGLGVNKPGGTIASTTYPTANALVKALMDAGCGWWRSHGVTESGMTTFGHGSSYFSYVSDTCSAINVAYETGRVIVLASNRAKINNGDQPARNVLYGSANPPDLNNETRGVLNMANGGTGSTNASDARLAFGLRMIDVPSNTSGATRCIKIATIKSPGAAGSFASMSIYGGSGIGSGPRVNIDTVIISGRNSGNENNPQGDVSILHRCLRAGTDTLKFGVVKTGTGQYDVYMKVMGYVQGLRIVVDTILSQSFIDGPVYTGSFGSIGYVNESEIPTPAGNIGWANTYDIVNQYNKDIAQEFSEVTIKRASTNDNEARLLFNFGKTESRGAELRSTYTNGNIVLSTGHNGESSPVFGEIYLRTAGSTNSTGQFKFDKEGSATATGGQWKNSSDIRLKRDFKPISSPLESVMSFRGATYEMKASGVRAIGVIAQDIEKRCPDAIGRMEIELDGEVIPDAMSVDTAGFAAAYSVQALKEVVKLMDLMLEDPEAASARIKALKAMINDELPE

>K63PH128 cds 22, tail spike protein

MALYREGKAAMAADGTVTGTGTKWQSSLSLIRPGATIMFLSSPIQMAVVNKVVSDTEIKAITTNGAVVASSDYAILLSDSLTVDGLAQDVAETLRYYQSQETVIADAVEFFKNFDFDSLQDLANQINADSESAQSSAAAAAASENAAKTSENNAKSSEVAAENARDQVQQIINDAGDASTLVVLANPDGFRHIGRCKDIATLRTIEPVESRQVIEVLSYYNGLAQGGGTFWYDPNDSVTEDNGGSCIVTNGGKRWKRIIDGAVDVLSFGAKPDDISFDSAPHIQAALDNHDAVSLYGRSYYIGSPIYMPSRTVFDGMGGKLTSIAPSTSGFMAGSIFAPGNYHPDFWEEVPKVAATTTLGSANITLADPNIVNVGDIIRLSSTTGVLSAGFFVSEYLQMARVLSKTGNVITIDGPVESQITLVAANANQPGYLARFNKPLFCCVDSIIQNIEIDTWDYWTADSATYNVKFSNIWGSAKAVAYGNTFCRSLFEDIRIVFSGRVSELAFGSHDTNLVRITAIASPNGLSASVVFGWAESGRRCTIDTFSIMLNANANPSTVIRVSGHRDSLIKNGSIYVHNNTNNILSVENYGTTADGVRPDCDNVTFENLSIFVTGSSAVVCDVYKSANDSVIKNVAFKNIKYFGPTPSVALYRARGTASNFVNGIQANISSDTGGSIALINSSNNDLTFIGPVSVPSLVSAAAKNNLVIRNSARSSAKGNDFTQEAPLNVTNTNTNAVIKELTYPAGSLRINDKIRLSLSGSTSGTAGKKTVQVGFVGSDSAFKYVELAALAADQVYWTMEVEISFLKTTNSQTQEIETSAIITSFLSKGVASGASVTGSRALSVVPDISLSNFVVQVRAWKENSGDGLSLSRMNLQLEDLTA

>K64PH164C4 cds 24, tail spike protein

MALYREGKAAMAADGTVTGTGTKWQSSLSLIRPGATIMFLSSPIQMAVVNKVVSDTEIKAITTNGAVVASTDYAILLSDSLTVDGLAQDVAETLRYYQSQETVIADAVEFFKDFDFESIQNLANQIKQDSETAESSAAAAAASEGAAKTSETNSKASEVAAEAARDQVQQIIDNAGEQSTLVVLAQPGGANRIGLQHGGKVQHLQTFLSFDMFGINKDGSSDVTSQIISVFNLANSLGLKVKQNDGVYLISGSDIITSNYGYDLEGATLRPASGYTGYILYTQVEQPVTYSAGSELITKINSAAISAGTSVLKGLRTDTTLNGKAIFMFGDNDPLYVSRGKTKNWWHISRISSRGKMDDACKYGVSEVTSIIALPINKKLTDIRLPNWDFVNEPANNGVMRFKNFSRCRIYGGQIFNRPINDNEKSPVIISMSYAYDVKFYDLYDEYPSWPIVGGSIAYAYTLNFNYCCRLEFNNCNSQGYGWGVVGGQLSCNITYRDCNLNRIDMHDPFSGYLKVIDCDIGFWGICATGMGDLYVERCTINLEDTAMNGYREHDGIISGRGDFGGWFDGKVYIRDLKIVGDAEAFRAANGRGVSLFSPYSFNATTGSIPSGSPVEPWGFKEIYVDGLHCDTPIVGKRFSSIVYAASVQYTTYFPRVIQIKNADFNSTEPECFDMHGFLVSPDNAAKTGIAHTLNFKPTNFIQMDDCSLAGVEIKRPYSQYDYNNLSFRARNLRQANGQDSPIEFWTDQVGRYEFDSCDIKKLSDSTETTSSVSSRQSTFAIRGGNFNSLTESPFNIQYNTGLSTQISVDGTIFIGPYSQTAVNAANLNLAEFATLSNCKVYSNETNGFVTPALWLGSTSSASTAFNVARGNTLDTVITVSNTNAGGDVVSGISTQVAKVPSGVVNGHISASFYVDATGTTGTYQLYLNARNTKAQVGKIVSNGKISGIYLQ

>K65PH164 cds 12, baseplate wedge subunit depolymerase

MTIAPFVTSLRIHKLSANQVNIRWDDVGANFYYFVELAETRNRAGEVIPADNLSWSSLGYTADNDWFEQNRIEPLTYYKMRVQTTSAGFEPSEWVETEEFQTFEENAYTFEHMQEFSLVKEFIKQKFSLNNMSYVNFNTSAMMASLMTESFQFSPEYSHLSAIENFVVGESGYHEIQGPIEAVCVDKNRTMLGEIDGILYLFERFQHMVKVSNDKGQNWQYVQLFNDRVGNPVSRVVIYQSKTTSYVLGYDKIFYGRKSSDVRWSSNEVKFSDNEVTFAKLGDQLKLGFEVELFGTYASLPADVTKYAEAFTCNDDYLYVVAKDTVRKVKLKDAPIDTDPLSPTFGEKVFEKEASHITGNPKSVCFKMDSVGGKIFALITGEVKTLGLDPTDPRNVVDSATKGVYVYQEGTNTWKRVFGNTDEEKRRIEHLWTSMSTDGKEIFFSSANFKTTEYAQDIELETKYPELISTAVKNVNPIQYHSDKHYHMMSFRADEFSRWETFVPGPMRFYAEPWFVWMAREGNRCWISTADHAVVIYNDILYQKRVDAAAQGTTERILSEVWDKGDATFYCPPVSFNGFLQYASGIMFHEPDGKLIGYYAFDYRVRDQVTLNWKPTDVMFKAFLQNQTREEDWTPEHTPGLRDPDLRPYLTKMMPDSYLLQDSNFEHFCKYYLQFLSDGNGTHYNSLVNLVKNKYPREENAWEYLWSEVYKRNIYLSKDARDAVVRFFEARKNDFYATKGIEDSYKFLFKLLYNEDVEIDIESKNTTEYDIIVESTNISDDLVGRTIYTASGRSNVTYIEREYRDGHLLWRITIHNLSGRFIEGQEIKSERTDFEGIIVQGVRGKDMLSNNIDYINRSRSYYVMKIKSQLPTSRFRDDVLRFVHPVGFGFIGITLLTMFINSGLNMKHVETIINKLKNYKWDAGLPSVYPDRVAIIASDDTIERDPITNEPRYSSRAQAGEPFPLPANYNQENNNSVIAGQNPGQRRKPLSPTFDQSAVTFANYRDLVNQRLKDDAGNPRDPENPTQVKIDE

>K65PH164 cds 197, chaperone dependant tail spike

MADQNLKQIQFKRTSTENKAPGADIVARGEIALNTHGRTLAIYTKDEADNVVQLAGKGVPFLDTSGTLSVDGTTTLKDNVTISPNKAINFETTDLSGAIVRHIVGKCATNDGWYIGAGGTSNSGILEIGTIDDGAETIQFVQRGAGNVEARKLVLLDGSGNTTLPGDLRLSTNKTVKINSGSTLVLEMGVGSNDVYIKNRRGVGVLQLTNDSNLTFRNSQVYYAMDGRGPGKSGTLLTNVENNRQAWQYTISAATASTARWVKVATIKHPGMSSSQLDLMISGGIDSGHGRHYVDFITLSGRNLTSWSTNSLDNWVEWRRVGSPSKTNVPEYYVVKNDSATDADASFDFYAKIPRYGNGLYVTVLNTAGYNGQDSGTVIIYETNQDTGDTGPSGSILVSMKQIFDSLAKPDFGDTTGTLPVNRGGTGATNVGDARNNLGLRTAAVRDVGESNGNLMEVGAFGIGGNGKSLVDITSDVDLMTRLKALGGTVFRANTASGYTGAPYYSHGTGFYGRASDTMAALNIDYATGNVRVFAINDSGLASGRVNSNVLYGTANKPSKADVGLGNLTNDTQVKKAGDTMTGDLAAPNLHASGTGTASVYVNAGSGNAHVWFRTDANERGVIWATPNTANLGQINIRAKTTGGTSAGDFSFRSDGRLGVPVAVKVGGAAMLTKDGNITSGSMFGGNLNNYLNSIKNDIVSGDNKQVSKTGDTMTGNLTINANLKVENPNGTMADFGSENSDKYSRITLARKIGSGAAVAMLKITPEGYVQFGYQDAVANPSPTKYIRVKPDGLDVEGDLVFNQTYCGTEEAVDISNKTIDLNNLVIKRTDPGTRQLYKCVSSGGGSKIANKPTSDGNFVLEVLSLRKVSDNDWTCKQTFTTKNNTTVGTYVRYCQNGSWTAWEEVVSGVQPINLGGTGATSVAAARNNLGVGEGQTVTFGNLVTTDLTANGNARLVGRLNLGSASATGVLRANETGAVVLGSASGQNIHVRAGSADTSAGETRFEPNGNVTVGGAITASGSLQVNGEAAMSRSLIVSQNIKNTNDNSFILMGKDSDLGFVKKSNSGAKLVFASGKSFIVAKSSATTIANPATETYTDVFKVDADGNQTVYGNSTVSRALTVSSTATVNGVINANGGIIVPTTKYVQIADAPTQNNQATNKKYVDDKVASAISNAGDTYLPLAGGTVTGNLDVTGSRLKTTSLWVTGDAAVNGVLTVDGKARFNQEFSVSTSVNVQNTGNSHVFFRKADGTEKGLLWADEPGNVSIRAGGASGPVWNFWTSGSCQFPGAISNYNGISSTTNYPAGQPNGTYNNTAGLVSRFSNGAYASLYFQEYVGNFHQAIINVNGFGRDDSFYFRAGGDFICTRNGSFDNVEIRSDRRAKSDIKVIENALEKVETLSGNTYELHNTSGGTTRSAGLIAQEVQEVLPEAVTQDIEADGGLLRLNYNSVIALLVESVKELSAEVKGLKAEIEELKSK

>K65PH164 cds 198, chaperone dependant tail spike

MADLKAGSTVGGNPIWHAGTFPLVPAGNSLTYRGKKVYTEIDKPQADDNDFVSKSRGGTYAGELKIVYGSSGKLSMGPLRDVGIRVGGTNGEVLVIGGNSSIQLRAGGTEDSSGIVEISKTGNLTAAGRITANVFTSRQQPTGVDDLTRKDYVDGLINTVSNVANAAVKKAGDTMTGVLRAEAGVVIVNTATSGEYAPRLDQVISRGVTIDFGTY

>K66PH128C1 cds 55, tail depolymerase

MAQSLEGTIQSLLQGVSQQVPRERQPGQLGAQLNMLSDPVSGIRRRPPGEIVWESTIDNPGLDSLFTEYVERGTDGRHLLINTSNGNWWLLAKNGKTILNSGNDPYFVTTVGQTSLQTASIAGLTYILNTEMAPNTTVDNTGRIDPSTTGFFYVKSAAFQKRWNVTVTSAGVDYSGDYTAPAAGSTSGNAEEVSGAYVAQQLRDSLVANGLPAGNVSVRGAYLFFYGLSNCVVSSDAGDTYAVVSNQSRVDQEQDLPAQLPAQADGAMCRVGTASSETAWYQFSYSTRTWSEVGAYGSITKITNMPRELAADDNIIARDWEGRLAGNDDNNSNPGFVENGYITGIAAFQGRLVLLSGSSVDMSASGLYQRFYRSTVTSLLDTDRISISSASAQDSVYRTAVQFNRDLVLFANSMQAVVPGSVVLTPTNASISITSTYECDSRVTPVMAGQTVIYPNKRNDSYAGILELIPSPYTSSQYTTQDATVHLPRYIPGRVLQMQNSSVTNMAFSRMSGERNSLLVYEFMWGGSDGAKMQAAWHKWSFPYPILSVQAMEDEVFLYMQGPSTSNKLLIVSMDPREGYQLGSEYREAYSDLQKQVQVQDSVFEVPAVLRPVGWADSYKEELILTYLPSNPMGPTEVGIKEIAGENTLRVVRGVPDGTYVVGRRYRSTFTLTTPILRDQNDKLVGSGHVRLLRLDVAVRNSGHFDVQVLDTPRDVNWGGELTGILMNSKELMLGQALRMDLATITVPCRTNADTTEVSLFTEGSMELNVLDISYILRYNQRRRRI

>K66PH128C1 cds 59, tail spike protein

MAFSWQESVKPAGTQDIQCDIEYLDKSYIHVYLDGAETTAFTWTSSTNIRLNSPLSAETAVLLIRKTERKYLYIEFASGAPFIEGNVDTQNMQLLHLAQELVEGRYIEGFYGDINMHRYRITNLGDPVDVRDATNKQYVDTEDARRDARIDAEAATRKAADDALDARVINLEQTYFNASTNSFSWWTVTTAPTSTITPGMLFTKAKVRLNGITQTAGYSYSVANGVITFAETVPAGTLVDVTIGIDTEADTSAVSTVLDLLASPQTNGVGVNVVHLTGAATVPFERFLADSGWNMDAAILAAAASGKTVLFRNDETYTISVNDIPFARYTSFIGNPGGNPPLFLITNPTGTYGTFNLYVSNGLNGGRYNKFDNLSFRYPNQVKTGSAIPYPPLFAGGCYGSEFTNLDIGNAYIGFRLGGTLNSVELGTASRVRLSDIIGAPLYRGLSLEQVRDVPTIQNIRWNYNYLDGGPYAYDSTLKQWMHDNAWAFQFGRLDWSLVSNLSAYGYLRGVVLLSTRYTGSADRLRFVGCTFDHTVNPVYLQNFSNSVDFIGCGFTGDRGSEFTRITPSNLYINNVGDTSAWVNFIDCTINNLTSDGIRTGSNLRLSGGTRLWGLGYDGTVETPRNGVYVTANNVSVDISASTIDVSAGQHTRCVFDGGMSGCMLTVSAGAKLIGASLEVFRWNGSTSNREVLSDDVYVPGTVTARGFLSFYSPRQVYRSASVPTVGTFARGERVLNMSPTVLGAAGSQYVVEGWLRLTSGDTHTMGVDWLELKQLTGS

>K69PH164C2 cds 24, chaperone dependant tail spike

MALYREGKAAMAADGTVTGTGTKWQSSLSLIRPGATIMFLSSPIQMAVVNKVVSDTEIKAITTKGAVVASTDYAILLSDSLTVDGLAQDVAETLRYYQSQETVIADAVDFFKTFDFEPLQGLADQVMADSEAADASASAAAASEGAAKTSEANSKASENAANSAKNDAQTAKSQTENLYNQTVDLVAGVQVPDKITGTIAREKWMKIANVKSAGDGYAFVQFIIGGGTDYGSANLPVDIFSLSGRGLPASPLTSDNIDIWFTQRTLISARPNDTRRINLGVVKNTDFSFDVYLHAPGGWIPELWLNRLNVQANNGSITGPIIDRAGYSWITTEPAGIVYNSPSNYLMANDSTIPRTNVANTFSQPQAISVPGGNATLTLNGAVVRANNNNAIVYSIPEGGQGMYFRPNGDMNSAKQVVFDAANFTVTGLNATFSNAVTMLSTLRVNGSSNLRGGVDVTASQKLPLKETTATTGIGVNFIGDNATECSFGIENTAGGSAVFHNYARGASNSATKNNQLLGGYGSRPWLGSAYTAHSNAALHFLGAGDASDTNHGGWIRLLVTPKGKNIGWRSPVMRISDNGDTWLVTAGSMVSPDLDEVRSFETLNAAVPKFNAPTNQDGRGLKIVADGAPEINMIAPRGSNASSPAVRAMWCDGSLGNSDKYIGATQAWSNFFFGASGHDGEKFDSMRGAVNIQASEGWGATSTPTRILFETCATGSTTRTSRWCVDHNGSFIPMGDGGYDIGWGSGRVNNIYAKNGAINTSDGRMKNDVRAMSDPETEAAKAIAKEIGFWTWKEQADMNDVREHCGLTVQRAMEIMESFGLEPFKYGFICYDKWDEQTVVSEYGPANEDGSENPIYKTIPAGDRYSFRIDELNMFIAKGFEARLSALEDKLGM

>K6PH25C3 cds 23, tail spike protein

MALYREGKAAMAADGTVTGTGTKWQSSLSLIRPGATIMFLSSPIQMAVVNKVVSDTEIKAITTNGAVVASTDYAILLSDSLTVDGLAQDVAETLRYYQSQETVIAEAVEFFKDFDFESLQNLANQVKADAETSATNAAAAAASKDAAKTSETNSKASEVAAETARDKIQQIIDDAGDQSTLVVLAQPTGGRKSGLEQGGTVQDALYKRVTVEAFAHLLTDPDDWSIAFAAASATAETIGASAVYCAAGNYKFKPTRGYDFVLPFDDGTYDSRRVGETTLPAETQHRMRVGLNWPSNISLIGDGISTTTFEFLWDNTTVDLNQTIGICLRVRNWDGTYAATVGAKNRMTSDIANIRLDGFTTKNAAIGIVADGTVGSCSWGELQFTNCGHAMAWQGGDRCTIKKLRFNNCAAGFTIGGWWLTRNDISGSNTGLGVPPYVAGTDVFCAGWSDFVIVESTEYTNSTEWESTDIFNKVDTFFDTYFYKTANSKRTADGGRCTNTGPNLTSASTLAEDRKFKGISKRAWSQIARYHRNHILNEARNVKTWGCNRKPILTSDNDDTELSVPGCIIGTAYIEHTGKRVIATRDYTVGGSNDFVTSGIDPWQDGETYNKYWCAEGNAVVMNGWPVNTLGLFASARTVARNDNGYANRMRRTIIDDGTGKPSIIEQLSPDVYILPPQKFIPNAVGSVDSRYYWKHFEKDVSSLMTLYAGVPTPDSQSVVDTTSKRVFMERVGNRVKLKVVFDASKYASGGDNPVVIGFTGIYTPVNSANPAFALNTYGPVKTVVISSKTFTINDTGGVARTFRLEPVAHESGGFVDSEGKQHYYFALQKQRSTDVAHRFLWSDVAGGRFQIEIEYDTADSITSSSVFPG

>K70PH128C1 cds 24, chaperone dependant tail spike

MATISDQLAADIQKAFNKYYTDLTNQDQIFFGVGDVKITKQDGSTATVRSWNKVIGALDTAAQRNAQNSFSSLQIFNAGLDVRIGNINCSGDGQMIYLGKNSDIGLVKKQGDGGKVVVGKSNGFRVHASTTNTVSATGESNEIMSVDGGGNLKLPGGVVAAGQGYFNSIELINATPYIDFHYGNNSAADFTHRILAEDGALSVAPGFRVKGPTGLYGICTQYGEAYGQGFISRLSNDPQNVAKGTILASPRVTVRFASRGADGNVDGGQGAMWFEEQVGTNHRLVLMVGGFGANVQYWQFLADGNIWSSANGGVQFTGTSDARFKHDINPTDGSESVKRLKALQLVTFIYNDDDQNRVRRGIVAQQARGVDPQYVKHIKTSVKGSDGQDVEIDKMQLDNNVIMMDTLAATKVLIERVERLEAEIKLLKGE

>K71PH129C1 cds 51, tail depolymerase

MAQSLEGTIQSLLQGVSQQVPRERQPGQLGAQLNMLSDPVSGIRRRPPGEIVWESTIDNPGLDSLFTEYVERGTDGRHLLINTSNGNWWLLAKNGKTILNSGNDPYFVTTVGQTSLQTASIAGLTYILNTEMAPNTTVDNTGRVDPSTTGFFYVKSAAFQKRWNVTVTSAGVDYTGDYTAPAAGSTSGNAEEVSGAYVAQQLRDSLVANGLPAGNVSVRGAYLFFYGLSNCVVSSDAGDTYAGVSNQSRVDQEQDLPAQLPAEADGAMCRVGTASSETAWYQFDYSTRTWSEVGAYGSITKITNMPRELAADDNIIARDWEGRLAGNDDNNSNPGFVENGYITGIAAFQGRLVLLSGSSVDMSASGLYQRFYRSTVTSLLDTDRISISSASAQDSVYRTAVQFNRDLVLFANSMQAVVPGSAVLTPTNASISITSTYDCDSRVTPVMAGQTVIYPNKRNDSYAGILELIPSPYTAAQYTTQDATVHLPRYIPGRVLQMQNSSVTNMAFSRMSGERNSLLVYEFMWGGSDGAKMQAAWHKWSFPYPILSVQALEDEVFLYMQGPSPSNKLLIVSMDPREGYQLGSEYREAYSDLQKQVQVQDGVFTVPAVLRPVGWADNYKEELILTYLPSNPMGPTEVGIKEIAGENTLRVVRGVPDGTYVIGRRYRSTFTLTTPILRDQNDKLVGSGHVRLLRLDVAVRNSGHFDVQVLDTPRDVNWGGELTGILMNSKELTLGQALRMDLATITVPCRTNADTTEVSLFTEGSMELNVLDISYILRYNQRRRRI

>K71PH129C1 cds 55, tail spike protein

MINMAYSWQESVKPAGTQDIQCDIEYLDKSYIHVYLDGAETTAFTWTSTTNIRLNSPLSADTVVLLIRKTEREYLYIEFASGAPFIEVNVDSQNKQFLHLAQELVEGRAIPGFYGNISMNGYRITNLEDPEDAQDAATKSYTDALHNAAVERADAQFARTLRVPEASVSLLPSAADRSWTSLGFDGSGQPVVQDPAGTGLWGYVPVVGSFEHGAQLNKRFEVLLWESTDEYWRWDGAMPKVVLPGSTPATAGGTGKGKWVDVTDATLRANLGSSEEGMGISLTALEQGGNGQDLAVFVSPQMSHSDPYSQFGTAMMTAFTTAMAMGIGEVRVPAGVYVLDSTVSVTLSMSMSLVFDPGVIIYVDSPIDAFNININGKHLDISGSNARIMSRWGSADGSQVAAWRLTDASLDKSLTANSLKVGTADNISKFGYAVYGSGLNLPVFNHNLLQGVIGIHLDSPIAGSATAHAMGAQLNGCEIYTDKEGVEVFNRGGLGAEGLLIFGGEIISQGTAIRITNSGLDSNSYLPPLVRIIGMHINAYQALYAKDVSRVDFVLNDVQGKYSSDAPINGFLELGGVQQFSHSCNKYTAIKIGTNTTGDDCQSVIYQFASTLTNAFFTSNGNIYQLDAMTRPAFDFSGTTNVTEIQVSNDRLVSAGAWVSNAYLAYVRLSPEMTIGNTGAAVGLDYSTKGAFSSGVLSLGTRPSQGFTYSIPASIVANSSVISQITFPSQMVGKEVNILLAAANVSFTHGTNMICPDQKSFVMTLPNAIKVFALNTTQCVILDVGGMANRHTDITSIPTSRTSPGYPGAEVFDSANMILYRYIAGYGWGEIEVKAIS

>K72PH164C2 cds 51, tail depolymerase

MAQSLEGTIQSLLQGVSQQVPRERQPGQLGEQLNMLSDPVSGIRRRPPAETRINPGIPSFNLDSMFVEYVERGTDGRHLLINTDTGNWWLLSKDTQVTVNSGQDDYFKASLGQTSLQTTSIAGLTYILNTEKAPSITRDNGDKIDPATTGFFYVKTSAFQKRWTVTVTTGGVDYTAFYNAPAAGTTAGNAEEVSAAYVIEQLRLELINQGFPAENVSTRGAYMFFFGMTNCVVGTDAGDTYAIVSNQSKVTLESDLPAQLPAAADGAMCQVGQASSEAAWYKYDFASRTWSETGAYGSISSIGNMPRELAADDQILVREWEGRLSGNDDNNEDPGFVVNGYITGIAAFQGRLVLLSGSTVDMSASGLFQRMYRSTVTSLLDTDRISISSASAQDSVYRTAIQFNRDLVIFANSMQAVIPGSAVLTPTNASISITSTFECDSRVMPTMAGQTVIYPSRRNSSYASILELIPSPYTSSQYTTVDSTVHLPRYIPGRVMQMQTSSVTNMAFMRYTGQRNALLVYEFMWGGGDGAKVQSAWHQWTLPYPILGVQALEDLVYVYMQGPGNALLVAAMDPREGYTLGQEYDTAYSDFPTTVTVSGGQFTVPSIFRYTNYHLSIKEELILTYPQGTAMAATEVGLNEFLGANIVSVVRGVPDGTYVLGRRYKSAMTLTTPTLRDRNDKLVGSGHVRLLRLDVALRNSGHFDVHVTDTPRDVDWSGELSGVLMNSKELVLGRTLRVDLAVVTVPCRTNADTTEIQFSTNGSQELNILDISYILRYNQRRQRV

>K72PH164C2 cds 55, Putative tail depolymerase

MINMAYSWQESVKPAGTQDIQCDIEYLDKSYIHVYLDGAETTAFTWTSTTNIRLNSALSADTAVLLIRKTEREYLYIEFASGAPFIEVNVDSQNKQFLHLAQELVEGRAIPGFYGNISMNGYRITNLEDPEDTQDAATKGYVDGLHDAVVARADAQFARTLRVPESSVNLLPPAAARSWTSLGFDGSGQPVVQDPAGTGLWGYVPAVGSFEQGSQLDQRFEVLLWESTDEYWRWDGAMPKVVLPGSTPATAGGTGKGKWIDVTDATLRRDLASSEEGRGGDLVKANEAHTVSDILRRMRTLGDVGAGRPVWGYDTTNDVKIYNAQAGEGADDIMMNGGFFLARKFRGTSRFAVASGIRGGRASVDDSGAFRAQVVSVSSSLGIANYGLHDGVSDYADALLPALESWETVASATYTANTVTISPETYSETLKNILKGDVIRTRHSSRYWGTVQSVNQQTGVITVDMWALKDNPDLTPENDGSGFYLNFIDKVWADNKNVIVQPNSAGLYATVGEFGALVQKAGMGYVNGMDMVLLSGSTQDATAAFLSRSGASGFAWLYGYNAQGNRFNYYSDEGSVTPYVGFMEKSNAIVGMKFRNKNTYSMAWTNSSDLTAVNPSALTTIIGPAGHVFRQPERILTISTSGMLNQFYPTTYVTAAGITITMPDITQMPVAGYVFKVRLFLAGTYTFNTYQGQTTINGNPQLTVTTTAGRQTIEIQFDGTFWQAFTR

>K74PH129C2 cds 46, tail depolymerase

MALVSQSIKNLKGGISQQPEILRYPEQGSLQVNGWSSETEGLQKRPPMVFIKSLGGRGYLGEDPYIHLINRDEYEQYYAVFTGNDVRVFDLSGYEYQVRGDRSYVTVNNPKDNLRMVTVADYTFIVNRTRQVRESQNLTNGGTFRDNVDALINVRGGQYGRKLEVNINGVWVSHQLPPGDNAKEDPPKVDAQAIAEALATLLRAAHPTWTFNVGTGFIHCIAPADTTLDIFETKDGYADQLINPVTHYVQSFSKLPLNAPDGYMVKIVGDTSKTADQYYVKYDKSQKVWKETVGWNISIGLDYTTMPWTLVRAADGNFDLGYHDWKDRRAGDEDTNPQPSFVNSTITDVFFFRNRLGFISGENIVMSRTSKYFEFYPPSVANYTDDDPLDVAVSHNRVSVLKYAVSFAEELLLWSDEAQFVLSANGVLSAKTAQLDLTTQFDVSDRARPYGIGRNIYYASPRSSFTSIMRYYAVQDVSSVKNAEDMTAHVPNYIPNGVYSINGSGTENFACVLTKGAPSKVFIYKFLYMDENIRQQSWSHWDFGDGVEVMTANCINSTMYMLMRNAYNVWIAAVDFKKNSTDFPFEPYRFHVDAKRSYRISETAYDIETNQTVVNVKDIYGASFSKGTMAICESDGKITEYEPTGSSWDSTPDIRISGDISGNDIVIGFLYDFQYVFSRFLIKQEQNDGTTSTMDSGRLQLRRAWVNYQDTGAFTVSVDNGNREFNYLVNARVGSTGLRLGQKATTTGQYRFPVTGNALYQKVSLSSFNASPVSIIGCGWEGNYTNRASGI

>K74PH129C2 cds 51, tail spike protein

MDQEIKTVIQYPTGSTEFDIPFDYLSRKFVRVSLVANDNRRLLSNITEYRYVSKTRVKILVDTTGFDRVEIRRFTSASERVVDFSDGSVLRANDLNVSQLQSAHIAEEARDSALMAMPEDGAGNLDARNRKIVRLAPGEIGSDAVNKDQLDTAVGDAGGILSDVKKVQQETYDYIEKFADDTALVRGVSWVYNQGSANGGETSILIDKPTRVLAVPYIEVNGLRQEVGYHFSFDIATQRITLVKPLVTGDFLVALTTESSVPVEDLLANPTGASSIGTSDGRNVQVVLDGLTADLQTVRSAVSPKEFGGVPNTDATAAIKLAIAAALVRKAPLDLRSGPWTITETIDLTDIGTVIADATGVLRVDPTTFTSKFSNKYAVTFGNPDVTFRQGRSSHVQVIGTLVVSGLNRAGALNGVYFKGSWFASNVVRVSGLNGSAIVLEAVWDSVFQSLSAELCGNESNYQIDVRGGGDTSNCLHIGRIQSERAYHKCLRISAIRSVFNTIHAERTAVLTTDDGSTNADGTKYTTLMFSLGNSVVNQLIHDATSGNAPDGRPTVGMASSRIDADYCVINAAGLSGSGLSSNSGRNTTWNGMYVRKWTFSGTATGHTIVSPRIIESLAPNNSITVKGGTAGQVSFGYNAKDVQIDSMAIDDLSFPNTIRGNINFTSCTFPETLTIGSTRAPEGYTAQSTLGETNTPVTFTDCVHLGTLAGAFQSRCVWKGGYIANISLVSRAVVELYNVSTKSFSATGDRAYITRQVRATNVGSWGVPTHVAYPIGTITERLGTDELNVGTGFRNADGTTTGFVKIY

>K74PH129C2 cds 52, tail spike protein

MLNNLNQPKGSTIGVLKDGRTIQEAFDQLDPVVSLSSTVSLATAVARLQITGGTIVVPAGTWEVPNLLNVPPSIQIIGRGNSSILKQPASSTSKGVLQFVLNNPTDSVRVDNLSIVTERPYQADNIAIRVDLRPQLSGDQIANRNVRRGSLTNLSIRGSTARTDGVSFGIGIDLVSTGWITLDNIHVVGSSSTATPYGFRGVGICQRGDGKPVETIIRGLMAVSLEYAYLCPEYTEGVYLTDSGAVNCKWGVVVSPVADYVIGKLGQVGGYQFVVDQFHPNVSQGGIFLSKCRYSFVSNVMAILDDHSVSSAVLTVHLRDSSYCNVNAVTAVSYNVNGTTGITRQVVVLNNTSDSFVGNISGLTEGTQYENLTNIVRLVNNSQRNEIEGVKGSNANEGISVGAGCGQNSIAKYRFSNVTTPVLDEAGDLNRGNTAAHYETVTPPPSSQEVTLEIVPRGYFSRRPQGFSYTITTPTDASFPIVVFYDRGSSTPTKVVLKVKSGVLGNNLPTVMIGISITLHD

>K7PH164C4 cds 20, chaperone dependant tail spike

MAITTRIIAQQVTALDGANSRVSKYPKFTVQLGYSVSSLAATELLDAATRSAASAAAAKTSETNAKASETASKNSQTAAKTSETNAAASAQLAQNVAGKASLVTPLGVMTGSAEAKIASITIASNQSSSVHLLFALYATGNGANRDDIYNMEIVSLALPGPVTSVTADNIGSFLSHRVIGPANTNGFMVGLKSTIEGSNVTYDVYLKSRSSFRDPKMAFLSGSISVTPPTGPLVDGTAPAWRTTGFDTDVIYVNRAQVIDDGISLARIKQLAITNGKTDSTILLLSYLNEIGILSTNKKSISLRPGGTSDSSIAATEFLPNGNIILPNGDTGNQTISWLGGPRIRVNSNGSFVLSTNNPSNQTSGFITFRPQGDQVTSTELQIRDDGNIKQTAPQSSAGNALIRQDAAIQHIMDKAPAAGITTNPLSDLNVIPTPEGTDPWGADGVRVFQSGVSTKNTPDGTTGRLGTILNVRHTQYRIMQFFMQSNATAPILHIRSLRADQGNTPPAWFKVYTEYSKPNIQSDIAGITIDGNGFVKKASPIAKLIAEIPSKEDSFFWTGVETVGGYVGCNAEAQGVFAVKTGLGKYTIKGSLGWNTEGWKFELPRDDNGNMLCFVESDWNEEEKELNIQVFTRKFDINTGNIIAGEPMEIPQGRWIDLRLEMPKVEIPEVELPEDPEV

>K7PH164C4 cds 151, putative tail depolymerase

MGFYAGRIGDKKVLSLTSGNNKDVNNHTNPGWDTIFHSDMPHVVVLETHERDLWDGGDWYRCTRMPDRIIQVLSADYDRVVLTEVEFEDGTRRFIYGTSLGVGAKAYNAYFSNTVGSQVSAGTMASMKTNVCASADLHMDISFYFEETPGTINEKLRDGTGCMYTWGVNSEWGDRGPGPPVGAPIRPNFETIIKAGWVLYRGAFSGNIAGSVSPPNRPLTIGVDAMRHPWMRTTGVNSICLRGETLNRNMYGHMGPRYGMGSNPVGGPYAHNIQTESYQEVQYKAGFFRGPPNNFMGWENTDNNNAGSGWGNNAIYRDNNFRVPKRVRWYITNMKYNGQGFYAENVFGSRNQEIKISPREFIVNGINLMNTGWKFINQNDINYSPGNRPDIRVIATNVARFSGNPTVGNNGYVHFNQPLTRPDNGAEFGQGNISEMHVTTVGVYNFRSDAQWYVKSNPPEIGNQWGPVWSEATRPLRLVGGTGSADIGGNLRTSGNASHHLATLWLGVNNSRNGACVVTLDWKNDEWIAAAGIGCYNPLEDLTQWSEVDSRLRIFGNHFQKRVHQIMCLPVNMCVPFHFIRGTVTQCGVIPGNNAMQMKAMWAPTTTNSATQGDYAIIYWLIARADGSVEVWVNVEMSNIMNMRVILPEVR

>K82P1 cds 40, tail depolymerase

MALVSQSIKNLKGGISQQPEILRYPEQGSLQVNGWSSETEGLQKRPPMVFIKSLGPRGYLGEDPYVHLINRDEYEQYYAVFTGNDVRVFDLSGYEYQVRGDRSYVTVNNPKDNLRMVTVADYTFIVNRTRQVRESPLQTNGGVFRDDVDGIINVRGGQYGRKLEVNINGVWVSHQLPPGDNAKDDPPKVDAQAIAEALAVLLRTAHPTWTFDVGTGYIHCVAPANTTLDVFETKDGYADQLINPVTHYVQSFSKLPLNAPDGYMVKIVGDTSKTADQYYVKYDKSQKVWKETVGWNISIGLEYHTMPWTLVRAADGNFDLGYHDWKDRRAGDEDTNPQPSFVNSTITDVFFFRNRLGFISGENIVMSRTSKYFEFYPPSVANYTDDDPLDVAVSHNRVSVLKYAVSFAEELLLWSDEAQFVLSANGVLSAKTAQLDLTTQFDVSDRARPYGIGRNIYYASPRSSFTSIMRYYAVQDVSSVKNAEDMTAHVPNYIPNGVYSINGSGTENFACVLTKGAPSKVFIYKFLYMDENIRQQSWSHWDFGDGVEVMAANCINSTMYMLMRNAYNVWIAAVDFKKNSTDFPFEPYRFHVDAKRSYRISETAYDIETNQTVVNVKDIYGASFSKGTVAICESDGKITEYEPTGSSWDSTPDIRISGDISGKDIVIGFLYDFQYVFSRFLIKQEQNDGTTSTTDSGRLQLRRAWVNYQDTGAFTVSVDNGNREFNYLVNARVGSTGLRLGQKATTTGQYRFPVTGNALYQKVSLSSFNASPVSIIGCGWEGNYTNRASGI

>K82P1 cds 45, tail spike protein

MDQEIKTVIQYPVGATEFDIPFDYLSRKFVRVSLVSDDNRRLLSNITEYRYVSKTRVKLLVATTGFDRVEIRRFTSASERIVDFSDGSVLRATDLNVSQLQSAHIAEEARDAALMAMPQDDAGNLDARNRRIVRLAPGIDGTDAINKNQLDTTLGEAGGILSEIIEVKGDFYEYLEKFAEDTSMVRGVVWVYNSGSAVGGETVVKVEKPTTVYSVPYLEINGSRQEIGYHYDFDPATQELTLAKPLVAGDFLMAMTTESYLPIESLLASPVGAEAIGTKSGKTIQRILDESPRYFTPEMFGAKGDGLTNDTKALQDAVDAAMSSGGGKVILKGNTTYLFDHLLVAREKSPQSRFEARLIIEGSGGSVLKHTGSITEAAHAFWVRGVLGTGSLADIYMRDVVLRDFSINGTVTSTANGLVLQRATAVRIENVYVNDFGGNGYRALDLYDSTIDSLEVQRCGIVPGATVGSYGMYITGASDQSNANHYIACRVEMCPLIIAIDKGCRHNYFHNCKFEQGRVNPTTSNPVYINDATELAFEACQFVQNYDSAIRFLVVTDALFPYWVTHGTEKVVNFTDCSFVCSRSVTAYWIDVAYTTFTACAFSSCNGGTQFPLSLGKNSFLTDAKVVIRTAEGNVLELKGAHSRVTNLKVTYFQQPTSGVFIKFTGLPGLADVVVDGFTFENFEPFAPYSGHTDFMGDVVVMRRAGYVYNGTNDRVIYGCSTLSYSGASPATWNNLRNGYNGQVVIVHAKSNPVTLDVSGGLIITKTGSNVTIPTNGVSALVNISGVWRQLY

>K82P1 cds 46, tail spike protein

MLKTDFNQPKGSTIGVLKDGSTVQQAFDTLGYTGGFLGEASVRAVRPTTNGQRIMANVQGVLSQWVYDAEDTVTADNGWFCLVTPEGHRWIREDGMFGIDISKGFVQGTDLSTKWQQAVDYSVYMAVTRKKTLRSATPIQLPTGECKMTKTVRCPSWIGNFVDGALSVDLSELPDNEVAFWCKGVQVNGLSTQGATTDVLSASNGIKFNGKGRDFGQIAIKVGNTATEWTNQGFNNGGSRFYGVVTWNCHTSVFLTNYNMYLAKFANFDFGIGQYGVVFGTYGEVSMPERDSGERIEFNGGVIYGMRYGIRFGMNAAFVDISNVSFDYTTYDVLSFANNTIYNMITLSKVHVEATGGWMIAGGNANSNNVINLDRVDNLTTLVGGAQVTETESNSLCRQMFNLNGGFTINCHMFGQLSPAQQPHNPKIWLNAGTAARIRQSNPSPMAAVPRPGSPADFLAAYTFNDSPLGTTIDNITTFTLTESVNVVKADSIVVDVGNGQRGIKIVTNTVGASGAYAVALTTPKFPVVGNSNYAAMLAYQPLLTGTAGSYIYTWTWYDLNGNVISTENYTGSVTAQRSDKAIIGYVEPGVVEDKRFLPVYAATRMAPANAATATITYRMAQTVGVFNIVNLYAWRTY

>K8PH128 cds 41, tail depolymerase

MALVSQSIKNLKGGISQQPEILRYPEQGSLQVNGWSSETEGLQKRPPMVFIKSLGPRGYLGEDPYIHLINRDEYEQYYAVFTGNDVRVFDLSGYEYQVRGDRSYVTVNNPKDNLRMVTVADYTFIVNRTRQVRENQNMTNGGTFRDNVDALINVRGGQYGRKLEVNINGVWVSHQLPPGDNAKEDPPKVDAQAIAEALATLLRAAHPTWTFNVGTGFIHCIAPADTTIDILETKDGYADQLINPVTHYVQSFSKLPLNAPDGYMVKIVGDTSKTADQYYVKYDKSQKVWKETVGWNISVGLEYHTMPWTLVRAADGNFDLGYHDWKDRRAGDDDTNPQPSFVNSTITDVFFFRNRLGFISGENIVMSRTSKYFEFYPPSVANYTDDDPLDVAVSHNRVSVLKYAVSFAEELLLWSDEAQFVLSANGVLSAKTAQLDLTTQFDVSDRARPYGIGRNIYYASPRSSFTSIMRYYAVQDVSSVKNAEDMTAHVPNYIPNGVYSINGSGTENFACVLTKGAPSKVFIYKFLYMDENIRQQSWSHWDFGDGVEVMAANCINSTMYLLMRNAYNVWIAAVDFKKESTDFPFEPYRFHVDAKRSYHISKTAYDIETNQTVVNVKDIYGASFSKGTVAICESDGKITEYEPTGSSWDSTPDIRISGDISGKDIVIGFLYDFQYVFSRFLIKQEQNDGTTSTEDSGRLQLRRAWVNYQDTGAFTVSVDNGSREFNYLVNARVGSTGLRLGQKATTTGQYRFPVTGNALYQKVSLSSFNASPVSIIGCGWEGNYMRRANGI

>K8PH128 cds 46, tail spike protein

MDQDIKTVIQYPVGATEFDIPFDYLSRKFVRVSLVADDNRRLLSNITEYRYVSKTRVKLLVETTGFDRVEIRRFTSASERIVDFSDGSVLRAADLNVSQIQSAHIAEEARDAALMAMPEDDAGNLDARNRKIVRLAPGEAGTDAINKNQLDETLGEAGGILSDFEDVRDEIIQYISKFTDDTGAVRGVSYVYNNGRALGGETGFHIDITPPPLGVPYLSINGSKQYRGYHFTYDPITGNVQGLAKPLEKDDFVVATTTESVTPIEDLYASPQGASMIGTLSGSTVEERIAEVEQSVIDSIDGVRVDSFIGMTDSGAIDAAITEARRVNSYVKFSPRVYTVDRPVILPSKTVLVGTQGLTKIVASASWNGPVVMSKDAPAGDYLTINVPSAMVYGVYIFGIIIESGWKGTTDDSRYHTECLRIYGAGTILKGVRVGKCRGDGANLGGRGLTAIDYGAPSLYSDVRADLIGKNGITIGGSSDNHTQNIVVRNAGLLEHDVYYCIGIGPGGGTRGNEYHTWHSGDAQITPLYQNRPKYGLYLAGWDTYITNGHFEGAASAQIANFGGRNQVTNVRAYSTWDPDKCTVLVAGPAFKFVGNIGAPMNNVPANRCHAFQLGTADIQVNQVEITAQVNGQKFVNYVNAGGSNSIAVRGTLGIAGVSGIGTLVTGTVPDANELTIIAEPYKGKRLGLNLVLTDDKGLTCRDVNSTGQISGVNALLTGKVMLTGLSSDIPTTPGTVYVDSNGNLKVKL

>K9PH25C2 cds 76, chaperone dependant tail spike

MTVKISGILKDALARPLANVAIRFLSLKTSSNIVIGVDTDFRTANDGSYDIDVVSGTYGVLMNFGSYEKIGEINVYNDSLPGTLEDFLTIPGIEEITPEILAQVIQARNNAVNAANNAASDATTIINEQLQNQKNEFDQFLLSSGYVFLGDYEDGPFQFSVRNQYIRYSNQYYRLDAATDVGFTTTGTDATSFASDVTHFVLMDGDTLRQNLGSSDGTSWVAKLGNKPLVAISYYKNQGLSDQDAVQAAFNESSNILIDHDIALTDYITFDRSEECYVYRKPGVTITGHGYLPKLRTNPAHVVETAIRHSKTSDRGGSYDRTYSHQSLAAEMVVHDVLSTDPGQENFVALYSGIESFNCQKQRMWAFNTVTSAHNLKTGDEIYGCEIDMNVDGTLDGGGQFVGVYIAGIGDVRTCANADGIRVQRLRDGVYKWQYGLRIFDSMTGINITDASTYSIFASGSAPIVRRKTTQDGGWSYTHSLSASSVKWGVDDYGDTYSRRLYLGTGDGKSKNRVNLDGGVSYYTTNAAVAWGSIAANAYVDKDITTLVGVSIADWTNYTIDVTPIGYAGAMPVVAVQAYINSTKTQAYVRIINISGAPLSSCNVGLNIKVSGHSATN
